# Phytochemical Characterization of Water Avens (*Geum rivale* L.) Extracts: Structure Assignment and Biological Activity of the Major Phenolic Constituents

**DOI:** 10.3390/plants11212859

**Published:** 2022-10-26

**Authors:** Anastasia Orlova, Elana Kysil, Elena Tsvetkova, Darya Meshalkina, Andrei Whaley, Anastasiia O. Whaley, Annegret Laub, Antonio Francioso, Olga Babich, Ludger A. Wessjohann, Luciana Mosca, Andrej Frolov, Maria Povydysh

**Affiliations:** 1 Laboratory of Cell Regulation, K.A. Timiryazev Institute of Plant Physiology RAS, 127276 Moscow, Russia; 2 Department of Bioorganic Chemistry, Leibniz Institute of Plant Biochemistry, Weinberg 3, 06120 Halle (Saale), Germany; 3 Department of Biochemistry, St. Petersburg State University, 199034 Saint-Petersburg, Russia; 4 Department of General Pathology and Pathological Physiology, Institute of Experimental Medicine, 197022 Saint-Petersburg, Russia; 5 Department of Pharmacognosy, St. Petersburg State Chemical and Pharmaceutical University, 197022 Saint-Petersburg, Russia; 6 Department of Biochemical Sciences, Sapienza University, 00185 Rome, Italy; 7 Institute of Living Systems, Immanuel Kant Baltic Federal University, 236041 Kaliningrad, Russia; 8 Department of Biochemistry, St. Petersburg State Chemical and Pharmaceutical University, 197022 Saint-Petersburg, Russia

**Keywords:** antioxidant, antibacterial, anti-neurodegenerative, *Geum rivale* L., high-resolution mass spectrometry (HR-MS), metabolomics, neuroprotective, phenolics, phytochemical characterization, structure assignment, tandem mass spectrometry (MS/MS), water avens

## Abstract

Water avens (*Geum rivale* L.) is a common *Rosaceae* plant widely spread in Europe and North America. It is rich in biologically active natural products, some of which are promising as prospective pharmaceuticals. The extracts of water avens are well known for their triterpenoid metabolites and associated anti-inflammatory, antimicrobial and antioxidant activities. However, the polyphenolic profiles of *G. rivale* L. are still awaiting complete characterization. Accordingly, the contribution of its individual components to the antioxidant, antibacterial and neuroprotective activity of the extracts is still unknown. As this plant can be available on an industrial scale, a better knowledge of its properly-relevant constituents might give access to new highly-efficient pharmaceutical substances and functional products. Therefore, herein we comprehensively characterize the secondary metabolome of *G. rivale* by ESI-HR-MS, ESI-HR-MS^n^ and NMR spectroscopy with a special emphasis on the polyphenolic composition of its aerial parts. Furthermore, a multilateral evaluation of the antioxidant, neuroprotective and antibacterial properties of the aqueous and ethyl acetate fractions of the total aqueous alcoholic extract as well as individual isolated polyphenols was accomplished. Altogether four phenolic acid derivatives (trigalloyl hexose, caffeoyl-hexoside malate, ellagic acid and ellagic acid pentoside), six flavonoids (three quercetin derivatives, kaempferol and three its derivatives and two isorhamnetin derivatives) and four tannins (HHDP-hexoside, proantocyanidin dimer, pedunculagin I and galloyl-bis-HHDP-hexose) were identified in this plant for the first time. The obtained aqueous and ethyl acetate fractions of the total extract as well as the isolated individual compounds showed pronounced antioxidant activity. In addition, a pronounced antibacterial activity against several strains was proved for the studied fractions (for ethyl acetate fraction the highest activity against *E. coli* АТСС 25922 and *S. aureus* strains ATCC 27853 and SG-511 (MIC 15.6 μg/mL) was observed; for aqueous fraction—against *Staphylococcus aureus* SG-511 (MIC 31.2 μg/mL)). However, the anti-neurodegenerative (neuroprotective) properties could not be found with the employed methods. However, the antibacterial activity of the fractions could not be associated with any of the isolated individual major phenolics (excepting 3-*O*-methylellagic acid). Thus, the aerial parts of water avens represent a promising source of polyphenolic compounds with antioxidant activity and therefrom derived human health benefits, although the single constituents isolated so far lack a dominant selectively bioactive constituent in the bioassays performed.

## 1. Introduction

Plants represent an important source of natural compounds promising of the development of new highly efficient drugs of high acceptance with patients [1]. The recent comprehensive analysis of global trends in development of pharmaceutical industry accomplished by Newman and Cragg indicated (again) the essential contribution of herbal medicines, which accounted 33% of all newly registered drugs in the period from 1981 to 2019 [2]. Moreover, an essential number of the registered pharmaceuticals designated as synthetics represented the analogues or/and derivatives of pharmacologically active natural compounds [3]. Historically, the plants, which were recognized as efficient preventive and therapeutic agents by traditional and official medicine, were regarded as rich sources of bioactive compounds. Moreover, plant-derived pharmaceutical preparations are not only highly efficient, but also, in comparison to synthetic drugs, are often featured with outstanding bioavailability and safety, strongly benefiting from the exceptional variety of natural products [4]. In this regard, the biological effects of plant extracts and their potential use as medicines have been intensively studied over the past decades [5,6,7]. Bioactive components of plant extracts are represented by an impressive diversity of secondary metabolites, which are typically involved in stress response [8,9], herbivore resistance [10] and protection against oxidative damage associated with vital physiological functions—photosynthesis, lipid/fatty acid metabolism and oxidative phosphorylation [11]. Not surprisingly, the secondary metabolites involved in these functions were repeatedly shown to exhibit a broad spectrum of pharmacological effects [12]. 

Polyphenols constitute one of the most represented groups of bioactive plant secondary metabolites. Their ability to suppress oxidative stress and to detoxify and scavenge reactive oxygen species (ROS) makes them efficient in prevention or therapy of the oxidation-related pathologies of the cardiovascular, nervous, urinary systems, as well as cancer and age-related diseases [13,14,15,16]. Moreover, polyphenols exhibit pronounced antimicrobial activity [17]. Hepatoprotective, cytotoxic, and anti-inflammatory properties of some groups of plant extracts and individual natural products were demonstrated as well [18,19].

Water avens (*Geum rivale* L.) is a widely-spread plant from the *Rosaceae* family. It can be found almost all over Europe, with the exception of areas of western France and Spain, and also grows in western Siberia, Central Asia and in some regions of North America [20]. Due to its wide distribution and relative ease of introduction into cultivation, water avens can be an economically profitable medicinal plant. Infusions and decoctions prepared from different parts of this plant are used in traditional medicine as an anti-inflammatory, antiseptic and astringent medicine since ages, but water avens is still not employed in official medicine [21]. Earlier, Pazzini et al. demonstrated the high antimicrobial potential of individual fractions of *G. rivale* L. aerial and underground parts [22], while Tunon reported the pronounced anti-inflammatory activity of the total water extract and demonstrated the antioxidant activity of extracts from the underground parts [23]. 

However, despite this progress, the polyphenol spectrum of *G. rivale* L. is still awaiting comprehensive characterization, with the contribution of its individual components for the antioxidant, antibacterial and neuroprotective activity of different *G. rivale* L. extracts is still unknown. As water avens is ubiquitously spread in Eurasia, can be easily cultivated and is available for isolation of individual polyphenols on an industrial scale, these questions are worth special attention in the context of a prospective therapeutic application. Indeed, this knowledge could give access to new highly-efficient pharmaceutical substances and functional foods. Therefore, here we comprehensively characterize the secondary metabolome of water avens with a special emphasis on the polyphenolic composition of its aerial parts. Further, we evaluated the antioxidant, anti-neurodegenerative (neuroprotective), antibacterial properties of the aqueous and ethyl acetate fractions of the total *G. rivale* L. extract, as well as the activities of some of its individual, isolated polyphenols with unambiguously assigned structure.

## 2. Results

### 2.1. RP-UHPLC-ESI-MS and MS^n^

As phenolic compounds were the main focus of this study, we employed LC-MS and LC-MS/MS for comprehensive analysis of the plant isolates rich in these compounds. Based on the available literature data [24], aqueous and ethyl acetate fractions, obtained by the re-extraction of the dried aq. alcoholic extract, were considered here. In a series of preliminary experiments, we determined the optimal load of the samples on the LC column. For this, both fractions of the total extract (8 mg/mL) were serially diluted with a twofold increment and the highest concentration of the isolates within the linear dynamic range (LDR) was selected for further experiments. Based on the dilution curves (data not shown) and the above mentioned criterion, the 1:8 dilution corresponding to the concentration of 1 mg/mL for the both fractions was selected for annotation of their individual constituents. 

**Figure 1:**
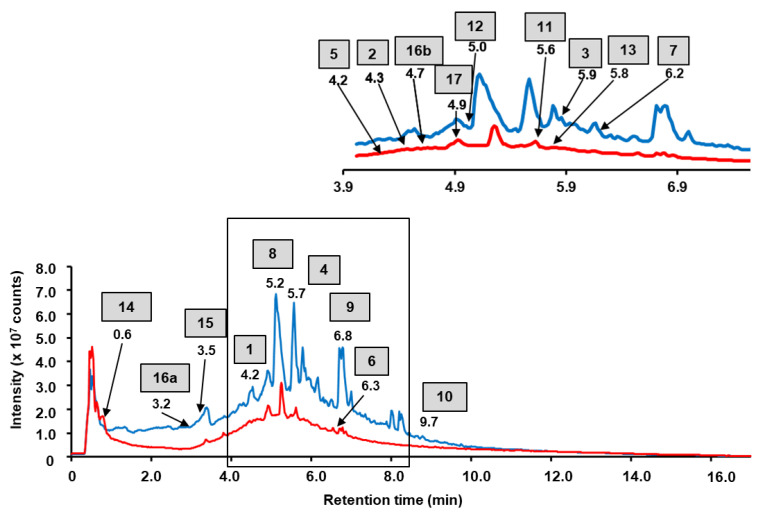
Total ion current chromatograms (TICs) obtained for aqueous (red line) and ethyl acetate (blue line) fractions of the total aq. ethanolic extract of water avens. The analysis relied on the RP-UHPLC-QqTOF-MS, accomplished with a Waters ACQUITY I-Class UPLC System coupled online to a Sciex TripleTOF 6600 mass spectrometer. The assignments could be confirmed by SWATH-MS/MS data (Supplementary information, Figure S3) and targeted MS/MS (Table 1, Figure S4).

The constituents of the fractions of the total extract were annotated by their elemental composition with a mass tolerance within 10 ppm (that corresponded to the specified mass accuracy of the instrument). As our study was activity oriented, for further structure characterization we considered only intense signals i.e., those exceeding 4.5 × 10^6^ counts in the MS1 spectra, which probably represented the compounds prospectively able to underlie pharmacological activities. In total, 146 signals were found in the total ion chromatograms (TICs) of the aqueous and ethyl acetate fraction of the total extract. Based on the above-mentioned criteria, 8 and 10 major phenolic constituents could be annotated in aqueous and ethyl acetate fractions of the total extract, respectively (Figure 1). For four and one features in the aqueous and ethyl acetate fractions of total extract, respectively, these assignments could be confirmed by the SWATH-MS/MS data (Supplementary information, Figure S3), whereas for the further four and nine annotations targeted MS/MS experiments were required. Finally, the structure of 13 compounds could be confirmed by the interpretation of their fragmentation patterns acquired in further targeted MS/MS experiments (Table 1, Figure S4).

#### 2.1.1. Phenolic Acid Derivatives 

The elemental composition of the trigalloyl-hexoside (compound **1**) was determined as C_27_H_25_O_18_—as can be deduced from the signal of the [M−H]^−^ ion at *m*/*z* 635.0908 (calculated for C_27_H_25_O_18_^−^ *m*/*z* 635.0890, see Table 1). During the MS/MS fragmentation based on the mechanism of collision induced dissociation (CID) in the linear ion trap, the loss of two galloyl units [M−H−152]^−^ resulting in the fragment ions at *m*/*z* 483.0735 and *m*/*z* 313.0553 could be observed. Further, the loss of dehydrated hexoside residue [M−H−144]^−^ yielded the fragment ion at *m*/*z* 169.0125 (Table 1, Figure S4-2). 

The elemental composition of the compound **2** was determined as C_19_H_21_O_13_^−^ ([M−H]^−^ at *m*/*z* 457.0988, calculated for C_19_H_21_O_13_^−^ 457.0988, see Table 1). Due to the presence of the fragment signal at *m*/*z* 341.0882 (loss of the malic acid moiety [M−H−116]^−^) and characteristic fragments at *m*/*z* 179.0333 the compound was assigned as caffeoyl-hexoside malate (Figure S4-3). 

The elemental composition of the compound **3** was determined as C_14_H_6_O_8_^−^. This assignment was based on the signal in the MS1 spectrum at *m*/*z* 300.9959, which corresponded to *m*/*z* 300.9990 predicted for C_14_H_6_O_8_^−^ (Table 1). Due to the presence of characteristic fragments in the MS/MS spectra at *m*/z 299.9874, 283.9932, 245.0060 and 173.0226, the compound **3** was assigned as ellagic acid (Figures S4-4 and S4-5). 

The ESI-MS^2^ spectra of the compound **4** assigned as ellagic acid pentoside ([M−H]^−^ at *m*/*z* 433.0366, corresponding to the elemental composition C_19_H_14_O_12_^−^) represented the CID fragmentation patterns characteristic for ellagic acid derivatives (Table 1, Figure S4-6).

#### 2.1.2. Flavonoids and Their Derivatives 

The ESI-MS/MS spectra of compounds **5** (*m*/*z* 563.0976, t_R_ 4.2 min, C_27_H_26_O_19_^−^), **6** (*m*/*z* 477.0638, t_R_ 6.3 min, C_21_H_18_O_13_^−^) and **7** (*m*/*z* 953.1265, t_R_ 6.2 min, C_42_H_34_O_26_^−^) shared the common characteristic fragmentation pattern of quercetin derivatives, which dominated with the fragment signals at *m*/*z* 301.0336 and *m*/*z* 477.0638 (Table 1, Figures S4-7–S4-9). Compounds **5** and **6** were found in the aqueous fraction of the total extract, and compound **7** was found in the ethyl acetate fraction of the total aqueous alcoholic extract of water avens. During the CID fragmentation of all these compounds, the loss of hexuronic moiety [M−H−176]^−^ accompanied with formation of the characteristic fragment at *m*/*z* 301.0336 ([M–H]^−^ of quercetin) was observed. Based on its fragmentation pattern, compound **5** was annotated as quercetin bis-hexuronide, whereas the compound **6** was assigned to quercetin-hexuronide and compound **7**—to quercetin-hexuronide dimer. 

Unfortunately, in many cases, mass spectrometry fails to distinguish structural isomers with identical elemental composition. Thus, unambiguous identification of the compounds demonstrating an intense signal at *m*/*z* 285.0384 in their MS/MS spectra appeared to be challenging. Indeed, this fragment is characteristic for both kaempferol and luteolin derivatives. Therefore, distinguishing these compounds by the acquired data was not possible.

The elemental composition of compound **8** (t_R_ 5.2 min, *m*/*z* 637.1046, C_27_H_27_O_18_^−^), compound **9** (t_R_ 6.8 min, *m*/*z* 923.1519, C_42_H_35_O_24_^−^), compound **10** (t_R_ 9.7 min, *m*/*z* 285.0394, C_15_H_10_O_6_^−^) and compound **11** (t_R_ 5.6 min, *m*/*z* 461.0692, C_21_H_18_O_22_^−^) were assigned based on the ESI-HRMS data (Table 1). Although **10** showed poor mass accuracy in the MS1 spectrum (Table 1), for all four compounds the acquired fragment spectra displayed typical signals characteristic for kaempferol or its derivatives (*m*/*z* 461.0708 and 285.0394). Based on this knowledge, **8** could be annotated as kaempferol-bis-hexuronide, **9**—as kaempferol-hexuronide, **10**—as kaempferol (Figures S4-10–S4-13) and **11**—as kaempferol-hexuronide (Figure S3-2). 

The elemental composition of **12** (t_R_ 5.0 min, *m*/*z* 667.1152, C_28_H_28_O_19_^−^) and **13** (t_R_ 5.8 min, *m*/*z* 491.0818, C_22_H_20_O_13_^−^) were also derived from the ESI-HRMS data (Table 1). Due to the presence of the characteristic loss of the hexuronide moiety [M−H−176] resulting in the fragment ion at *m*/*z* 315.0501 (isorhamnetin aglycone), the compound **12** was assigned as isorhamnetin-bis-hexuronide (Figure S4-14) and **13**—as isorhamnetin-hexuronide (Figure S3-3). 

#### 2.1.3. Tannins 

The key fragmentation reaction of ellagitannins is the release of bislactone and the formation of a hexahydroxydiphenoyl-ester group, which, due to its instability, undergoes lactonization with the formation of ellagic acid [39]. Therefore, even under relatively low collision energies, the base peak in the MS/MS spectra of ellagitannins can be always detected at *m*/*z* 300.99 as the characteristic ellagic acid fragment (Figures S3-4–S3-6 and S4-15–S4-16). The major neutral losses accompanying the fragmentation of ellagitannins during MS/MS analysis include galloyl (152 u), hexahydroxydiphenoyl (HHDP) (302 u), galloylglucose (332 u), and galloyl-hexahydroxydiphenoylglucose (634 u) [40]. 

The elemental composition of compound **14** was determined as C_20_H_19_O_14_^−^ at *m*/*z* 481.0615, calculated for C_20_H_19_O_14_^−^ at *m*/*z* 481.0624 (Table 1). Due to the presence of characteristic fragments in the SWATH-MS/MS spectra at *m*/*z* 300.9988, 275.0195 and 133.0143, this compound was assigned as hexahydroxydiphenoyl-hexoside (HHDP-hexoside). (Figure S3-4). The same fragmentation patterns could be observed for the compounds **16a** and **16b** obtained from the different fractions of the total aq. ethanolic extract. The elemental composition of compounds **16a** and **16b** was determined as C_34_H_25_O_22_ at *m*/*z* 783.0634 (calculated for C_34_H_25_O_22_ at *m*/*z* 783.0686). Based on their fragmentation patterns, compounds **16a** and **16b** were annotated as pedunculagin I (bis-HHDP-hexose) (Figures S3-5 and S4-16). For the compound **17,** the elemental composition C_41_H_28_O_26_ could be assigned at *m*/*z* 935.0780 (calculated for C_41_H_28_O_26_ at *m*/*z* 935.0796). Under CID conditions, the loss of a gallic acid residue [M−H−168]^−^ resulting in the characteristic fragment at *m*/*z* 300.9988 was clearly observable. Based on this pattern, compound **17** could be annotated as galloyl-bis-HHDP-glucose (Figure S3-6). The elemental composition of compound **15** C_30_H_26_O_12_ at *m*/*z* 577.1263 (calculated for C_30_H_26_O_12_ at *m*/*z* 577.1315) was determined based on the ESI-HRMS (Table 1). Due to the presence of the characteristic fragment ion signals at *m*/*z* 425.0803, *m*/*z* 407.0704 and *m*/*z* 289.0673 in its MS/MS spectra, **15** was assigned as proanthocyanidin (Figure S4-15).

### 2.2. Biological Activity of the Aqueous and Ethyl Acetate Fractions of the Total Extract

#### 2.2.1. Antioxidant Effects 

The aqueous and ethyl acetate fractions obtained from the total aq. ethanolic extract of *G. rivale* L. aerial parts differed in the patterns of their antioxidant activity. Thus, as can be seen from Table 2, in terms of free radical scavenging in the TEAC tests, the aqueous fraction appeared to be more active than the organic one (3.7 μmol/L vs. 0.03 μmol/L Trolox equivalents/μg of sample). In addition, the aqueous extract was more efficient in neutralization of the superoxide anion in the NBT assay in comparison to the ethyl acetate fraction (3.22 vs. 6.01 nmol of O_2_•^−^/min). Both extracts demonstrated high normalized activity in the DPPH test (98% and 94% respectively, Table 2).

#### 2.2.2. Antibacterial Activity

The antibacterial activity of the aqueous and ethyl acetate fractions of the total extract obtained from the aerial parts of water avens was tested against several strains of Gram-positive and Gram-negative microorganisms. The assays revealed essentially higher efficiency of the ethyl acetate isolate. Thereby, the highest activity was observed against the Gram-negative *E. coli* АТСС 25922 (MIC 15.6 μg/mL vs. 62.5 μg/mL for the aqueous extract) and Gram-positive *S. aureus* strains ATCC 27853 and SG-511 (MIC 15.6 μg/mL for both strains vs. 31.2 µg/mL and 62.5 µg/mL for the aqueous extract respectively). The lowest activities of both extract fractions were observed against the Gram-positive strains of *M. luteus* CIP A270 and *L. monocytogenes* EGD (MIC 62.5 µg/mL for ethyl acetate extract vs. 250 µg/mL for aqueous extract, Table 3). 

#### 2.2.3. Cytotoxicity Assay

**Figure 2:**
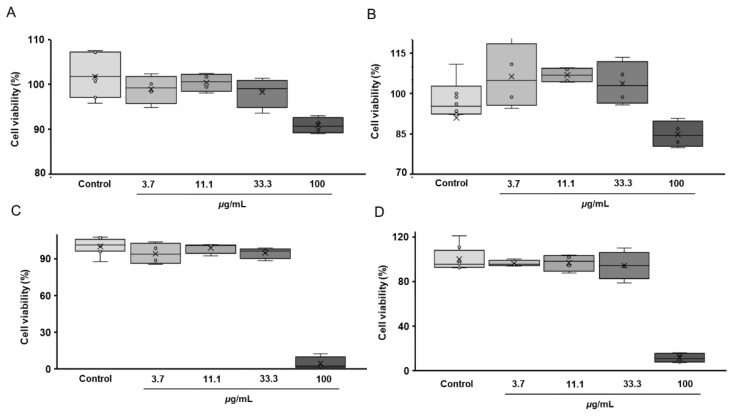
Results of the MTT assay (*n* = 4) addressing cytotoxicity of the aqueous (**A**,**B**) and ethyl acetate (**C**,**D**) fractions of the total *G. rivale* L. aqueous ethanolic extract, and performed 24 (**A**,**C**) and 48 (**B**,**D**) hours after supplementation of the plant isolates to the cell culture. The data are represented as median, interquartile range, minimal and maximal values.

Assessment of cytotoxicity is a pre-requisite for adequate activity screening. Therefore, prior to analysis of the anti-neurodegenerative (neuroprotective) effects of the aqueous and ethyl acetate fractions of the total *G. rivale* L. extracts, the toxicity of these fractions was evaluated with an MTT assay using the SH-SY5Y human neuroblastoma cell culture. For this, the aqueous and ethyl acetate fractions were dissolved in water or DMSO respectively and serially diluted in culture medium to obtain following dilution range: 100, 33.3, 11.1 and 3.7 µg/mL. As can be seen from Figure 2, independently from the treatment duration (both 24 and 48 h) and extract fraction (aqueous or ethyl acetate), 33.3 µg/mL was the maximal non-toxic concentration. As this result was confirmed in the second replicate experiment (Figure S5), this extract concentration was used in further experiments. 

#### 2.2.4. Neuroprotective (Antineurodegenerative) Activity of *G. rivale* L. Fractions of Total Extracts in a Cellular Model of Alzheimer’s Disease

**Figure 3:**
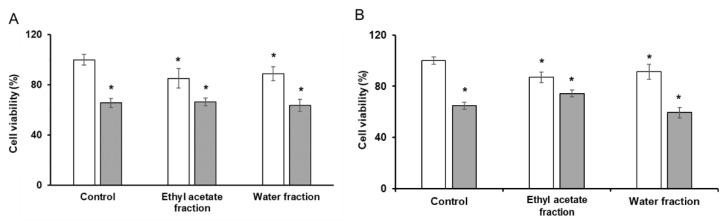
Results of the MTT cell viability test accomplished with SH-SY5Y cells with aqueous and ethyl acetate fractions of the total ethanolic extract of *G. rivale* L. Cells were treated for 24 h (**A**) and 48 h (**B**) in the absence (control) and presence of the *G. rivale* L. fractions of total extract with (grey) and without (white) supplementation of 25 µmol/L Aβ25–35. The assay was performed in quadruplicates. Mann–Whitney test and Bonferroni correction for multiple comparison were applied to address statistically significant differences observed in treated cells: *—*p* ≤ 0.05 vs. untreated with Aβ25–35 negative control. The controls were supplemented with fresh medium without addition of the fractions of total extract.

The analysis of the anti-neurodegenerative (neuroprotective) activity of the extract fractions upon their incubation with the SH-SY5Y cells for 24 and 48 h showed no anti-Alzheimer activity. Thus, although some tendencies for increasing cell viability upon supplementation of the studied fractions could be observed, these alterations were insignificant, i.e., the fractions of the total extract could not restore the Aβ25–35-induced suppression of cell viability (Figure 3). 

#### 2.2.5. Neuroprotective Activity of Aqueous and Ethyl Acetate Fractions of the Total *G. rivale* Ethanolic Extract in a Cellular Model of Parkinson Disease

**Figure 4:**
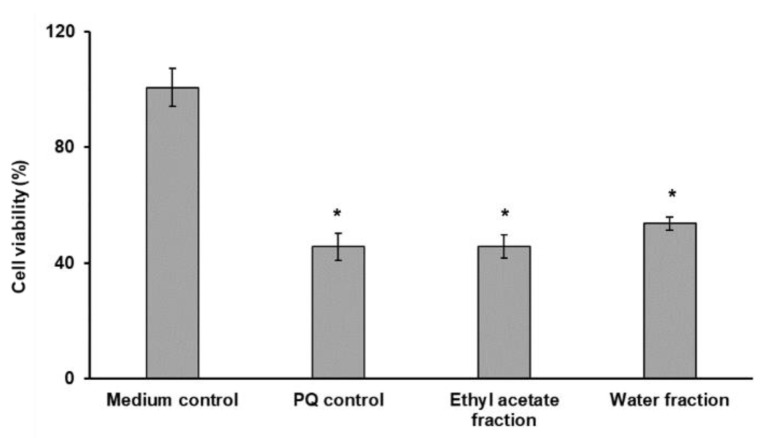
The results of the cell viability assay (*n* = 3) obtained in the paraquat model of neurodegeneration established with differentiated SH-SY5Y cells with aqueous and ethyl acetate fractions of the total extract of *G. rivale* L. Mann–Whitney test with Bonferroni correction for multiple comparisons were applied to address the confidence of the differences observed in the treated cells: *—*p* ≤ 0.05 vs. untreated with Aβ25–35 negative control. The negative controls were supplemented with fresh medium without addition of the fractions of total extract.

In agreement with the results of the cytotoxicity assessment, the anti-Parkinson effects were also addressed with 33.3 µg/mL of plant isolates. However, both fractions showed the absence of neuroprotective (anti-Parkinson) properties of both *G. rivale* L. isolates in the cellular model of Parkinson disease. Thus, these fractions of the total extract failed to restore the cell viability after application of strong pro-oxidant paraquat (Figure 4).

### 2.3. Isolation of the Major Phenolic Constituents of the G. rivale L. Fractions of Total Extract in Their Individual Form

As the observed antioxidant and antibacterial activities of the aqueous and ethyl acetate fractions of the total *G. rivale* L. extract could be underlied by the properties of their major components, the specific activities of the major individual extract constituents were addressed. For this, the major seven individual compounds, recently reported as the constituents of *G. rivale* L. extracts [20], were isolated in sufficient yields and purities (Table S6). Based on the HPLC data, the purity of all isolated compounds was at least 90%, whereas the structure of the analytes was assigned by 1D and 2D NMR spectroscopy (Table S8–S13). The structure identity of all isolated compounds was additionally confirmed by multistaged LIT-Orbitrap-MS^n^ (Figures S6–S12). The MS^n^ fragmentation patterns (Schemes S1–S7) were in agreement with the NMR data.

### 2.4. Quantification of the Major Phenolic Constituents in the Aqueous and Ethyl Acetate Fractions of the Total G. rivale Extract

The absolute quantification strategy relied on ultra-high-performance liquid chromatography coupled on-line to tandem quadrupole-time-of-flight mass spectrometry (UHPLC-QqTOF-MS/MS) and the standard addition approach. The resulting chromatograms were successfully integrated using the settings listed in Table S7. The overall contents of the seven major analytes (Table 4) were higher in the ethyl acetate fraction in comparison to the aqueous one. Two compounds, namely 6″-(4-hydroxycinnamoyl)-astragalin and 3-*O*-methylellagic acid were barely present in the aqueous extract: below the limit of quantification (b.l.q.) and 0.0043 ± 0.0007 μg/mg, respectively; whereas in the ethyl acetate isolate 9.8 ± 1.75 and 39.2 ± 1.45 μg/mg were found, respectively. However, as can be seen from Table 4, bis-glucuronides of kaempferol and isorhamnetin were 4–5-fold more abundant in the aqueous extract in comparison to the ethyl acetate one (1.22 ± 0.16 vs. 0.23 ± 0.03, and 1.88 ± 0.07 vs. 0.48 ± 0.04 μg/mg of the dry extract, respectively).

### 2.5. Biological Activities of Individual Metabolites Isolated from the Aqueous and Ethyl Acetate Fractions of the Total G. rivale L. Extract

Establishing the profile of biological activity of individual compounds obtained from the studied fractions of total extract of water avens is an important step in the understanding of the activity profiles of individual constituents of plant extracts and their fractions that might be employed for evidence-based medicine. 

In our study, aqueous and ethyl acetate fractions of the total ethanolic extract isolated from the *G. rivale* L. aerial parts showed pronounced antioxidant and antibacterial activities. Therefore, to dissect their activity profiles, we addressed the antioxidant and antibacterial effects of the major characterized compounds isolated from the aerial parts of water avens. Moreover, as no neuroprotective activity could be revealed in the models of Parkinson and Alzheimer disease with both fractions of *G. rivale* L. studied (i.e., these extracts could not restore cell viability after application of paraquat or truncated Aβ peptide), the study of these activities at the level of individual isolated natural products was not followed further.

#### 2.5.1. Antioxidant Activity of *G. rivale* L. Fractions of Total Extract and Isolated Compounds 

As in the first set of the experiments, the comparative analysis of the antioxidant activities of individual natural products isolated from the *G. rivale* L. fractions relied on three different tests (DPPH assay, NBT assay and TEAC assay) (Table 5). It was found that isorhamnetin-3-*O*-β-D-glucuronide, kaempferol-bis-3,7-*O*-β-D-glucuronide and caffeoyl malate obtained from the aqueous extract, as well as ellagic acid derivative 3-*O*-methylellagic acid and 6″-(4-hydroxycinnamoyl)-astragalin from the ethyl acetate extract, exhibited the most pronounced activity in the free radical removal in DPPH assays. On the other hand, 3-*O*-methylellagic acid and caffeoyl malate demonstrated a pronounced ability to scavenge superoxide anion free radicals (which are relatively stable) in the TEAC assay (2.93 and 3.07 μmol/L Trolox/μg of sample respectively). In this study, only three compounds, namely 6″-(4-hydroxycinnamoyl)-astragalin (1.8133 nmol of O_2_**^•^**^−^/min), as well as natural compound isorhamnetin-bis-3,7-*O*-β-D-glucuronide (3.4767 nmol of O_2_**^•^**^−^/min) and kaempferol-3-*O*-β-D-glucuronide (4.6489 nmol of O_2_**^•^**^−^/min), were active in suppressing superoxide-anion-mediated NBT transformation in the corresponding test. Remarkably, 6″-(4-hydroxycinnamoyl)-astragalin had the highest capacity for scavenging of superoxide radical anions among all the compounds tested (Table 5).

#### 2.5.2. Antibacterial Activity of the Compounds Isolated from *G. rivale* L. 

It was shown that the antibacterial activity of the individual compounds against selected Gram-positive and Gram-negative strains appeared to be essentially lower in comparison to the values obtained for the aqueous and ethyl acetate fractions of the total extract of *G. rivale* L. aerial parts. Among the natural products studied, only a derivative of ellagic acid, 3-*O*-methyl-ellagic acid, demonstrated sufficiently high activity against Gram-negative *E. coli* АТСС 25922 (MIC 62.5 μg/mL) as well as against both Gram-positive *S. aureus* strains ATCC 27853 and SG-511 (MIC 62.5 μg/mL and 31.2 μg/mL, respectively, Table 6). 

## 3. Discussion

The integrated approach assuming simultaneous analysis of plant secondary metabolome (i.e., qualitative and quantitative characterization of multiple biologically active natural products) and the patterns of biological activity characteristic for the same plant isolates represents a new milestone in the development of pharmacognosy and ethnopharmacology giving a direct access to the structure-activity relationships (SAR) [28].

Water avens (*Geum rivale* L.) is well known in ethnopharmacology, although it is still not considered by the evidence-based medicine in plant therapy protocols. Phytochemical characterization of this plant and identification of individual natural products as its constituents was started in the fifties of the last century by Blinova and co-workers [41,42]. To date, the composition of essential oils isolated from the aerial and underground parts of water avens has been characterized. It has been shown that the main components of the essential oil were pinene derivatives and eugenol [43,44]. Thereby, the composition of aliphatic alcohols, mono- and sesquiterpenoids as the principal components of these oils was revealed, but the pharmacological properties of the essential oil of water avens compounds were not shown [22,45]. Moreover, polyphenols which, along with triterpenoids, represent the major class of secondary metabolites in this plant species remained less studied, although they are also a promising group in terms of their biological activity.

In general, most of the work on chemical characterization of the water avens extracts report confirmation of already known (e.g., from other *Rosacea* species) metabolites by plane chromatographic methods and well-defined authentic standards [46,47,48,49,50]. 

In this work, we are making a step forward and address the composition of polyphenols in plant tissues. For this, we employed untargeted metabolite profiling which relied, in the first line, on high-resolution mass spectrometry (HR-MS) in combination with RP-UHPLC—a powerful technique to address diversity in bioactive molecules in plant extracts. Thus, our work is, to the best of our knowledge, the first implementation of this approach in the study of the aerial part of water avens. 

Several secondary metabolites, annotated in this study, were reported earlier as the components of water avens extracts, although their specific contents therein mostly remained unknown. Among such compounds were ellagic acid and its derivatives, kaempferol, kaempferol-glucuronide, and quercetin-glucuronide–which were isolated from the shoot [22]–and pedunculagin, which had been identified earlier by mass spectrometry [48]. In addition to those previously reported ones, several other compounds were identified here as the constituents of *G. rivale* L. aerial parts for the first time. Among them were five phenolic acid derivatives (three of which were caffeic acid derivatives), six flavonoid derivatives and three tannins. Importantly, besides the earlier reported data for ellagic acid [51], the absolute contents of the major constituents of the *G. rivale* extracts (Table 4) were determined here, to the best of our knowledge, also for the first time. 

Comprehensive characterization of the polyphenol secondary metabolite profiles made it possible to make a decision about the optimal strategy for the isolation of the major components from the aqueous and organic fractions of the total extract obtained from the aerial parts of water avens. Based on the intensities of the chromatographic signals in the corresponding TICs (Figure 1) and in the UV chromatogram acquired at 280 nm (Figures S1-1–S1-2), seven major components of the *G. rivale* L. preparations were selected for isolation and purification at semi-preparative scale. Among them, one compound, namely kaempferol-3-*O*-β-D-glucuronide (**12**), was earlier reported for water avens, whereas the further six natural products were not previously described for this species. Moreover, two of these six natural products were only recently described in our previous work, so far exclusively from water avens [20]. Thus, here we continue characterization of these compounds and provide further information—MS^n^ data, absolute contents and characteristic biological activities.

The structure identity of all isolated natural products was unambiguously confirmed by 1D and 2D NMR spectroscopy. Thus, the isolated compounds were structurally identical to those described earlier as the major secondary compounds described in the extracts of water avens [20], although their absolute contents in individual fractions of the total ethanolic extract were obtained here for the first time. Analysis of this data clearly indicated the ethyl acetate fraction as the more enriched one with the major metabolites (both in terms of variety and absolute amounts), than the aqueous one. Thus, our work delivers both qualitative and quantitative information, which is absolutely mandatory as a basis for interpretation of the biological activity data. 

Oxidative stress plays a critical role in the pathogenesis of age-related diseases, which are predominantly manifested by dramatic metabolic shifts, systemic inflammation, cardiovascular and nervous systems disorders [52,53,54]. That is why the knowledge about the antioxidant properties of plant extracts and their individual constituents might give a new insight in the action mechanisms of corresponding plant-based pharmaceuticals (both existing and prospective) and give access to new efficient tools for prevention and treatment of a broad array of pathologies. 

Currently, three major mechanisms of polyphenol-associated antioxidant activity are known: (i) transfer of a hydrogen atom from a functional group to a free radical, (ii) electron transfer from the polyphenol to the free radical under formation of a radical cation and subsequent rapid and reversible deprotonation in solution, followed by a second round of the same (e.g., from a hydroquinone to a quinone), and (iii) metal chelation [55,56]. 

To get a deeper insight in the antioxidant properties of the *G. rivale* L. fractions of the total aq. alcoholic extract (both aqueous and ethyl acetate) and individual compounds listed in Table 4, three assays with different mechanisms of antioxidant activity (DPPH, TEAC and NBT assays) were employed. Earlier, it was noticed that isorhamnetin-3-*O*-β-D-glucuronide and kaempferol-3-*O*-β-D-glucuronide at concentrations of 1 and 10 μmol/L significantly inhibited the production of ROS by f-MLP stimulated neutrophils [57]. Significant antioxidant activity of extracts containing uronide derivatives of flavonols, including kaempferol and isorhamnetin, has been repeatedly reported [57,58,59,60,61,62]. The same was the case with the extracts containing 6″-(4-hydroxycinnamoyl)-astragalin, although no information on the activities of isolated compounds is available. The antioxidant properties of bis-glucuronide derivatives are also still uncharacterized [57,58,59,60,61,62]. A decade ago, Cho et al. reported high radical scavenging activity of 25 µmol/L caffeoyl malate in a DPPH assay. The DPPH radical-scavenging activity of caffeoyl malate was very similar to that of caffeic acid, which was used as a positive control [63]. The mechanism of the antioxidant activity of ellagic acid derivatives, including 3-*O*-methylellagic acid, was comprehensively studied. The ability of this compound to exhibit free radical scavenging activity due to the presence of catechol moiety and a guaiacyl moiety was shown [64]. 

After analysis of the acquired data, we concluded that the antioxidant activity of both extract fractions did not essentially exceed the antioxidant activity of individual compounds in the concentrations selected for the analysis based on preliminary optimization. Expectedly, a relatively high capacity of both aqueous and ethyl acetate fractions of the total *G. rivale* extract for scavenging of stable free radicals appeared to be comparable to the activity of each of the isolated compounds. Thus, our results do not contradict the literature data, as 3-*O*-methylellagic acid and other catechol-containing compounds (caffeic acid and isorhamnetin derivatives) were shown to be potent free radical scavengers in the DPPH test. However, considering the low tissue contents of each of the considered individual compounds in the fractions, we cannot claim that the antioxidant activity of the fractions is mediated by specific compound, even if its content is relatively high. Based on this, we hypothesize that the high activity of the fractions in antioxidant tests is a possible consequence of the synergistic activity of multiple components, also those not isolated and characterized in terms of this study. 

Formation of amyloid structures is underlaid by oxidation of the corresponding proteins [65]. The pathophysiology of amyloidogenesis is strongly implicated in oxidative stress through a variety of mechanisms, including induction of protein oxidation, as well as nucleic acids and lipids, glycation end products formation, mitochondrial dysfunction, β-amyloid deposition, and plaque formation. Therefore, it is logical to assume that natural products with pronounced antioxidant effects might possess anti-amyloid and generally anti-neurodegenerative (neuroprotective) activity [66,67,68]. However, despite rather high antioxidant activity of both the fractions and isolated compounds, the aqueous and ethyl acetate fractions did not show any neuroprotective properties in the cell models of the Parkinson and Alzheimer diseases. 

The etiology of these diseases is multifactorial and complex and is not completely clarified. Several studies show that the neuroprotective and anti-neurodegenerative effects of plant extracts–including those rich in polyphenolic compounds–are mediated not only by a decrease in intracellular oxidative stress, but also by a decrease in neuroinflammation, initiation of autophagy and protection of neurons from apoptotic cell death [69]. To date, the efficiency of water avens extracts as neuroprotective preparations is not confirmed. Here, we confirmed its absence at the level of the extract fractions. As no effect could be observed at this level, we did not address the anti-neurodegenerative properties of individual compounds.

Evaluation of the antibacterial activity of the aqueous and ethyl acetate fractions of the total *G. rivale* extract showed promising results. Significant antibacterial activity of both fractions of the total extract was found with both Gram-positive and Gram-negative strains employed herein. Thereby, the activity of the ethyl acetate fraction appeared to be significantly higher than the activity of the aqueous one. This fact might be explained by the higher total content of the above mentioned major polyphenolic metabolites but also the better membrane permeability of more lipophilic compounds [70]. The antibacterial assays accomplished with the isolated individual components, however, revealed much less pronounced antibacterial activity or even its complete absence. However, one of the analyzed compounds, namely 3-*O*-methylallagic acid isolated from the ethyl acetate fraction, showed some activity. The acquired data on its antibacterial activity were in agreement with the previously published data [71]. Apparently, in the case of ethyl acetate and aqueous fractions of the total extract of water avens, we can either talk about the manifestation of the synergistic effect of the individually weak constituents in the extracts, or there might be a minor compound of pronounced activity yet not isolated. Any of these possibilities might result in well-pronounced antibacterial activities of the total extract fractions and explains the lack of the antibacterial activity of individual isolated major natural products. In addition, the superior antibacterial activity of the ethyl acetate fraction (in comparison to the aqueous one) can probably be explained by the four-fold higher contents of the ellagic acid derivative, which has significant antibacterial activity in its individual form. 

## 4. Materials and Methods

### 4.1. Plant Material 

This study relied on the aerial parts of water avens (*Geum rivale* L.) cultivated in the medicinal plant growing facility of the St. Petersburg State Chemical and Pharmaceutical University (SPCPU). The plants were harvested in July 2019 at the stage of completely formed flowers. An herbarium specimen (SPCPU19-GR) was deposited at the Pharmacognosy Department of St/ Petersburg State Chemical and Pharmaceutical University. The collected material was air-dried at room temperature (RT, 25 °C) without access of direct sun light. The dried material was grinded with a cutting mill (DM-6, HT Machinery, Japan-Taiwan) and the grinded dry mass was passed through a sieve with 1 сm pore diameter.

### 4.2. Materials 

Unless stated otherwise, materials were obtained from the following manufacturers: Duchefa Biochemie (Haarlem, The Netherlands): dimethylsulfoxide (DMSO, >99.9 atom % D); Ecos-1 (Moscow, Russia): *n*-hexane (analytical grade), ethyl acetate (analytical grade), methylene chloride (analytical grade); Honeywell (Seelze, Germany): acetonitrile (>99.9%, LC-MS grade), methanol (LC-MS grade). All other chemicals were purchased from Merck KGaA (Darmstadt, Germany). Water was purified in house with a water conditioning and purification system GenPure Pro UV-TOC system (resistance 18 mΩ/cm, Thermo Fisher Scientific, Langenselbold, Germany).

### 4.3. Extraction, Fractionation, Isolation and Structure Elucidation

Air-dried aerial parts of *Geum rivale* L. (1000 g) were extracted three times for 24 h with 7 L of aqueous 70% (*v/v*) ethanol at RT. The obtained extracts were combined, and the solvent was partly evacuated to 500 mL under reduced pressure in a rotary evaporator (Heidolph, Schwabach, Germany). The crude extract was exhaustively re-extracted by liquid-liquid partition via sequential treatment with *n*-hexane, dichloromethane and ethyl acetate, (500 mL each) using a glass separation funnel. The resulting polyphenol-enriched ethyl acetate fraction was evaporated to the volume of 50 mL. The aqueous residues after liquid-liquid partitioning were concentrated to a volume of 50 mL. The individual compounds were isolated from the aqueous and ethyl acetate fractions of the total extract using a procedure described in our pervious study [40] with some changes. In detail, the extracts were separated by chromatography on a HP-20 column using a six-step gradient from 0% to 100% aq. EtOH in 20% increments (300 mL for each step). For the second isolation step, Sephadex LH-20 column and isocratic elution with 100% EtOH were used. Seven resulting fractions were further separated by preparative reversed-phase high performance liquid chromatography (RP-HPLC) with UV-detection at 254 nm. The concentrated samples (15 mL) were injected in a Knauer Smartline system (Knauer Wissenschaftliche Geräte GmbH, Berlin, Germany) and separated at 40.0 mL/min on a Kromasil C18 preparative HPLC column (250 × 30 mm, 5 μm, Sigma Aldrich, Steinheim, Germany) thermostated at 40 °C. The separation relied on the linear gradient elution mode with 0.1% (*v/v*) aq. trifluoroacetic acid (TFA) and acetonitrile as eluents A and B, respectively. After a five-min wash-out the unbound fraction at 5% eluent B, the samples were separated in a linear gradient to 70% eluent B in 46 min. Afterwards, the column was washed with 95% eluent B for 5 min and re-equilibrated at 5% eluent B for 5 min. 

The ^1^H-, ^13^C NMR and 2D-NMR spectra of the isolated natural compounds were recorded in DMSO-*d*_6_ (Merck KGaA, Darmstadt, Germany) on a Bruker Avance III 400 NMR Spectrometer (Bruker, Bremen, Germany) equipped with a 3 mm broadband probe (^1^H, ^31^P, ^13^C, ^15^N) and with cooled ^1^H and ^13^C pre-amplifiers (1H at 400 MHz and ^13^C at 100 MHz). Acquisition parameters included a 45 hard pulse angle, a sweep width of 14 ppm, 3.27 s acquisition time, 1 s relaxation pulse delay. Up to 2000 scans were collected per sample, corresponding to ~1 h of collection time. 

### 4.4. Metabolite Profiling 

The profiling of the secondary semi-polar metabolites relied on the procedure of Leonova et al. [72] with minor changes. In detail, the lyophilized aqueous and ethyl acetate fractions of the total aq. alcoholic extract were reconstituted in methanol at 0.5, 1, 2, 4 and 8 mg/mL and analyzed by reversed phase-ultra-high performance liquid chromatography coupled online to quadrupole time-of-flight mass spectrometry (RP-UHPLC-QqTOF-MS). The samples (2 µL) were injected (partial injection mode) in a Waters ACQUITY I-Class UPLC System consisting of Binary Solvent Manager, FL Sample Manager, UPLC eLambda 800 nm (operated in the wavelength range of 190–650 nm at a resolution of 1.2 nm) and separated at 300 µL/min on a Waters ACQUITY UPLC BEH C18 column (2.1 × 50 mm, particle size 1.7 µm, Waters GmbH, Eichborn, Germany) at 40 °C. The separation relied on the linear gradient elution mode with 0.3 mmol/L aq. ammonium formate and acetonitrile as eluents A and B, respectively. After a two-min wash-out of the unbound fraction with 5% eluent B, the samples were separated in a linear gradient to 95% eluent B within 17 min. Afterwards, the column was washed with 95% eluent B for 2 min and re-equilibrated at 5% eluent B for 9 min. The column effluents were infused on-line in a hybrid QqTOF mass spectrometer (Sciex TripleTOF 6600, AB Sciex, Darmstadt, Germany) operated in negative ion mode using a sequential window acquisition of all theoretical mass spectra (SWATH) algorithm. The nebulizer (GS1), drying (GS2) and curtain (CUR) gases were set to 60, 70 and 55 psig, respectively, while the ion spray voltage was set to −4500 V. The MS experiments were accomplished in the TOF-scan mode (accumulation time 100 ms) in the *m*/*z* range of 65–1250. The tandem mass spectrometric (MS/MS, MS^2^) experiments were accomplished in the SWATH mode. Thereby, the overall *m*/*z* range (65–1250 *m*/*z*) was split in 48 windows (mass ranges) of 26 *m*/*z* each with an overlap of 1 *m*/*z*. Each *m*/*z* window was acquired with 20 ms accumulation time at the collision potential (CE) of −35 V with a collision energy spread (CES) of 15 V and declustering potential (DP) of −35 V. Nitrogen was used as collision activated dissociation (CAD) gas. Annotation of the individual analytes relied on the Reaxys database, literature data, and manual interpretation of the fragmentation patterns obtained in the MS/MS experiments.

### 4.5. Targeted Tandem Mass Spectrometry (MS^n^) Experiments

For all features which were annotated based on their [M−H]^−^ ions observed in the full MS spectra to polyphenolic structures with mass accuracy better than 10 ppm, but did not yield unambiguously interpretable fragmentation patterns in SWATH mode (typically due to simultaneous fragmentation of two or more intense *m*/*z*), additional targeted RP-UHPLC-MS/MS experiments were accomplished with a TripleTOF 6600 mass spectrometer (AB Sciex, Darmstadt, Germany) using the LC conditions summarized in Table S1 and source settings described in chapter 2.4. The MS/MS conditions were set as follows: each analysis was performed with 100 ms accumulation time at the range of collision potential from −10 V to −60 V with collision energy spread (CES) of 0 V and declustering potential (DP) of −35 V. Nitrogen was employed as collisional activation dissociation (CAD) gas. 

The high-resolution MS^n^ analysis of the isolated compounds was performed with a Dionex UltiMate 3000 UHPLC system coupled online to a hybrid linear ion trap-orbital trap mass spectrometer LTQ-Orbitrap Elite (both Thermo Fisher Scientific, Bremen, Germany) equipped with a heated electrospray ionization (HESI) ion source operated in negative ion mode. The stocks of the authentic standards in DMSO (10 mmol/L) were diluted with methanol, and 100 pmol of each standard were injected individually using the chromatographic conditions provided in Section 2.4 (for instrument-specific LC-settings see Supplementary Materials Table S1). The MS analysis relied on LIT-Orbitrap HR-MS scans using the settings summarized in Table S2. For the multistage LIT-Orbitrap-MS^n^ analysis the collision energy was optimized, and the spectra were acquired under the collision settings summarized in Table S3. The acquired raw data were evaluated with Xcalibur software version 2.2 (Thermo Fisher Scientific, Bremen, Germany).

### 4.6. Absolute Quantification of Phenolic Metabolites with Identified Structures 

Quantitative experiments were accomplished for the major constituents: kaempferol-3-*O*-β-D-glucuronide, kaempferol-bis-3,7-*O*-β-D-glucuronide, isorhamnetin-3-*O*-β-D-glucuronide, isorhamnetin-bis-3,7-*O*-β-D-glucuronide, 6”-(4-hydroxycinnamoyl)-astragalin, caffeoyl malate, and 3-*O*-methylellagic acid (Figures S1-1 and S1-2). For these, authentic standards were available in-house or purified from aerial parts of water avens. Their structural identity and purity were confirmed by NMR spectroscopy according to the scheme established previously [20]. The analyses relied on RP-UHPLC-QqTOF-MS under the instrument settings summarized in Section 2.4, whereas the absolute quantification strategy relied on the standard addition approach.

For this, the lyophilized ethyl acetate and aqueous fractions of total *G. rivale* L. aq. alcoholic extract were reconstituted in methanol and water-methanol 2:1 (*v/v*) mixture, respectively to obtain 5 mg/mL solutions. Individual authentic standards (2–20 mmol/L in 1.8 µL of DMSO, *n* = 3) were spiked to the fractions of total extracts in the concentrations of 9–90 μmol/L, centrifuged (14,000× *g* for 10 min at 20 °C) and the supernatants were serially diluted in 5–6 steps to obtain the minimal concentrations of 0.0625–0.625 μmol/L, based on the intensities of characteristic signals and with consideration of the linear dynamic ranges of individual analytes (Table S4). The data processing (annotation of the signals and integration of the corresponding extracted ion chromatograms at specified t_R_) relied on PeakView™ (version 2.2) and MultiQuant™ (version 3.0.2) tools (both AB Sciex, Darmstadt, Germany). Contents of individual analytes were obtained from the intercept on the x-axis of the curve based on one sample without addition of the standard and 5–6 spiked samples (Table S5).

### 4.7. Antioxidant Assays 

The antioxidant effects of the plant isolates (fractions of total extract and individual compounds) were addressed by 2,2-diphenyl-1-picrylhydrazyl (DPPH) free radical scavenging, Trolox equivalent antioxidant capacity (TEAC) and nitroblue tetrazolium (NBT) assays. They were done according to Masci et al. [73] with minor modifications as follows. 

#### 4.7.1. DPPH Free Radical Scavenging Effect 

The aqueous and ethyl acetate fractions of the total aq. alcoholic extract and isolated individual compounds were solubilized in DMSO at the concentration of 10 mg/mL. The aliquots of each sample were 10-fold diluted (final concentration 1 mg/mL) and 10 µL of these solutions (10 µg in total) were supplemented to 1 mL portions of 40 μmol/L methanolic solution of stable nitrogen centered free radical DPPH•. The absorbance was monitored photometrically at 517 nm after 1 h incubation at RT. The capacity of the samples for scavenging of the DPPH• radical was estimated from the difference in the absorbance acquired in presence and in absence of plant isolates. The corresponding values were expressed as the percentage of DPPH• consumption as a function of the sample concentration [74].

#### 4.7.2. Trolox Equivalent Antioxidant Capacity (TEAC) Assay

The 2,2′-azinobis(3-ethylbenzothiazoline-6-sulfonic acid) diammonium salt (ABTS) was dissolved in water to obtain a 7 mmol/L solution which was further oxidized to corresponding radical cation (ABTS^+∙^) in the presence of 2.45 mmol/L potassium persulfate (K_2_S_2_O_8_) and incubation for 16 h at RT in the dark. The radical cation reagent (ABTS^+∙^) was diluted with ethanol to achieve the absorbance of 0.70 (±0.02) at 734 nm. Aliquots of samples diluted as described in the previous section were supplemented to 1 mL of the ABTS^+^• solution. Absorbance was measured at 734 nm after six minutes of incubation in the dark at room temperature. Antioxidant capacities of the samples were reported as Trolox equivalents.

#### 4.7.3. Assessment of Extract Capacity to Scavenge Superoxide Anion Radicals (NBT Assay) 

The stock solutions (1 mmol/L) of phenazine methosulfate (PM) in ethanol, nitro blue tetrazolium chloride (NBT) in water, and *β*-NADH in 0.05 mol/L phosphate buffer (pH 7.4) were freshly prepared daily. The reaction mixtures contained 73 μmol/L β-NADH, 15 μmol/L PM, 50 μmol/L NBT, and 10 μg of samples in 1 mL of 0.02 mol/L Tris-HCl buffer, pH 8.0. The absorbance was determined at 560 nm immediately after mixing the reagents and after 15 sec of reaction. The change of absorbance in time (ΔAbs/min) and absorption coefficient of 1 μmol/L formazan solution 0.03 were used to calculate the rate of production of superoxide anion radical.

### 4.8. Antibacterial Assays 

The minimum inhibitory concentrations of *G. rivale* L. aqueous and ethyl acetate fractions of the total extract and individual isolated compounds were determined by microdilution broth method, as recommended by Clinical Laboratory Standards Institute, USA [75]. The following bacteria strains were cultured under aerobic conditions according to the approved standard protocol: *Escherichia coli* ATCC 25922, *Pseudomonas aeruginosa* ATCC 27853, *Listeria monocytogenes* EGD, *Staphylococcus aureus* ATCC 25923, *Staphylococcus aureus* SG-511, MRSA ATCC 33591, *Micrococcus luteus* CIP A270. The bacteria were cultured on a solid medium containing 3% (*w*/*v*) tryptic soy hydrolysate (HiMedia, India). Strains MRSA ATCC 33591 and *Listeria monocytogenes* EGD were provided by Prof. R. Lehrer (University of California Los Angeles, USA); Escherichia coli ATCC 25922, *Pseudomonas aeruginosa* ATCC 27853, *Micrococcus luteus* CIP A270, *Staphylococcus aureus* ATCC 25923—Department of Molecular Microbiology, IEM; strain *Staphylococcus aureus* SG 511—by Professor H.G. Sahl (University of Bonn, Germany). The microorganisms were cultured for 2–6 h in 2.1% (*w*/*v*) Mueller–Hinton broth (HiMedia, India) at 37 °C on an orbital shaker. When the turbidity of the bacterial suspensions reached 0.5 McFarland (1.5 × 10^8^ CFU/mL), all cultures were diluted with sterile 2.1% (*w*/*v*) Mueller–Hinton broth to obtain the concentration of 1.0 × 10^6^ CFU/mL.

The stock fractions of the total extract of *G. rivale* L. (1 mg/mL, 0.5 mL) were serially diluted (seven steps with a two-fold increment) with sterile 2.1% (*w*/*v*) Mueller–Hinton broth and 50 μL aliquots were added to the wells of a 96-well sterile U-Bottom shape plate (Greiner Bio-One, Kremsmünster, Austria). Afterwards, the wells were supplemented with the same volumes of bacterial suspension. The controls were established by supplementation of (i) 100 μL of 2.1% (*w*/*v*) Müeller-Hinton broth medium (*n* = 3), (ii) 50 μL of bacterial suspension in 2.1% (*w*/*v*) Müeller -Hinton broth medium (*n* = 3), (iii) 3) 50 μL of bacterial suspension in 2.1% (*w*/*v*) Müeller -Hinton broth medium containing 2% (*v/v*) DMSO (*n* = 3) as a positive control.

The microtiter plates were incubated in a thermostat for 18 h at 37 °C. The minimum inhibitory concentrations (MICs) were calculated as the lowest extract concentrations at which the growth of microorganisms in the corresponding wells could not be visually detected (that was interpreted as completely inhibited). The final results were calculated as the medians based on the data from three independent experiments, each accompanied with the complete set of the controls.

### 4.9. Assessment of Anti-Neurodegenerative Effects

Evaluation of the anti-neurodegenerative (neuroprotective) effects relied on cellular models of Alzheimer and Parkinson diseases established with SH-SY5Y human neuroblastoma cells and amyloid peptide Aβ25–35. 

#### 4.9.1. Cell Culture

Human neuroblastoma SH-SY5Y cells were obtained from ICLC (Genova, Italy). Cells were cultured in DMEM/F12 with 10% fetal bovine serum (FBS), 100 U/mL penicillin and 100 μg/mL streptomycin, 2 mmol/L *L*-glutamine in the atmosphere of 95% air and 5% CO_2_ at 37 °C and humidity ≥ 95%. Cells were passed twice per week by trypsin detaching (2–3 min followed with dilution with full medium) with 0.25% solution of 1:250 trypsin in phosphate buffered saline (PBS), pH 7.4 (this ratio corresponds to the trypsin activity: 1 part of trypsin digests 250 parts of casein). Before the trypsin treatment, the cells were washed with 1–3 mL of PBS, pH 7.4. The cells were not split in ratios lower than 1:3–1:5. Cell number counting relied on an automatic cell counter (TC20, BioRad, Hercules, CA, USA) or manual calculation with a Neubauer chamber.

#### 4.9.2. Synthesis and Aggregation of Aβ25–35 Amyloid Peptide

The amyloid peptide Aβ25–35 was synthesized by conventional solid phase chemistry (Atherton and Sheppard 1990), and stored at −20 °C. The peptide was resuspended in 1,1,1,3,3,3-Hexafluoro-2-propanol (Sigma^®^, catalog number #105228), incubated for 1 h with gentle shaking at 4 °C, then aliquoted and dried in speedVac for 20 min. The aliquots were stored under a vacuum glass bell. The day before being used, aliquots were resuspended in phosphate buffered saline (PBS) at a final concentration of 1 mM, incubated in an ultrasonic bath on ice for 30 min to induce aggregation, followed by gentle shaking at 4 °C overnight. Aβ25–35 was diluted in culture medium at a final concentration of 30 µM for cell treatments. 

#### 4.9.3. Anti-Alzheimer Assay 

Cells were seeded at the density of 1.5 × 10^4^ cells per each well of 96-well cell culture microtiter plates. Next morning, the medium was discarded and 30 µL of fresh medium supplemented with highest non-toxic concentration of fractions of total extract (33.3 mg/L) (*n* = 4) were added to each well. Two hours later, 20 µL of 25 μmol/L aggregated Aβ25–35 peptide was added. The control cells were supplemented with 50 μL of fresh medium. After the incubation for 24 and 48 h at 37 °C, the methylthiazolyldiphenyl-tetrazolium bromide (MTT) test was accomplished in quadruplicates. For this, 5 mg/mL MTT solution in PBS (pH 7.4) was added to obtain the final concentration in culture medium of 0.5 mg/mL. After a 2 h incubation at 37 °C in a CO_2_ incubator (Heraeus, Thermo Scientific, Milan, Italy), the medium was removed, and formazan crystals were dissolved in 100 µL/well of DMSO. Absorbance was measured in an Appliskan^®^ plate reader (Thermo Scientific, Milan, Italy) at 560 nm with a reference wavelength at 690 nm. Cell viability was calculated for each well as a ratio of specific absorbance to mean absorbance obtained for untreated cells (with blanks subtracted). 

#### 4.9.4. Cell Viability Assay of Paraquat Treated SH-SY5Y Cells

For the assessment of anti-Parkinsonian activity of the total extract fractions we used the paraquat model of neurodegeneration established on differentiated SH-SY5Y cells. The day before differentiation, the cells were seeded into the wells of a 96-well plate at the concentration of 50,000 cells per well. SH-SY5Y cell differentiation was induced by culture growth in DMEM/F-12 medium with 3% FBS containing 10 μmol/L retinoic acid and 10 μmol/L phorbol myristate acetate. Differentiation proceeded for 10 days with medium exchanged every two days and was routinely controlled visually by phase contrast microscopy (Figure S2). Neurodegeneration was modeled by the toxicity of 800 μmol/L paraquat incubated during 24 h with differentiated cells resulting in 50% culture viability.

To assess the viability, SH-SY5Y cell culture was seeded in the wells of 96-well microtiter culture plates at the density of 5 × 10^4^ cells per well. To determine the neuroprotective effect of fractions of total extract, SH-SY5Y cells were incubated with paraquat in the presence or in the absence of the potentially protective isolates for 24 h. 

### 4.10. Statistical Analysis 

The numerical results were expressed as the mean (mean ± standard deviation). Statistical significance of inter-group differences was assessed by the Mann–Whitney test (*p* ≤ 0.05) with Bonferroni correction for multiple comparisons applied at the confidence level of *p* ≤ 0.05. 

## 5. Conclusions

In this study we addressed the antioxidant, neuroprotective and antibacterial properties of the aqueous and ethyl acetate fractions of the total *G. rivale* L. extract along with the major constituents purified from these isolates. Although the both fractions showed no neuroprotective (at least anti-Alzheimer or anti-Parkinson) effects in cell-based models, they demonstrated pronounced antioxidant and antibacterial activity. Thereby, antioxidant effects of the fractions appeared to be the result of the synergetic activity of the whole complex of natural compounds. Moreover, the isolated compounds in the selected amounts showed promising antioxidant potential. The antibacterial properties of the aqueous and ethyl acetate fractions of the total extract were more pronounced in comparison to the isolated compounds, i.e., were manifested only for corresponding biologically active complexes, which included also minor constituents. Thus, both aqueous and ethyl acetate fractions of the water avens extracts represented complex mixtures of biologically active compounds with promising antioxidant and antibacterial activities. 

That is why the biologically active complexes of water avens can be recommended as potential sources and components of new functional foods promising for the prevention and initial or long-term therapy of various mild infectious diseases. The antioxidant effects of the secondary metabolites isolated from the aerial parts of *G. rivale* L. can be efficiently implemented in diverse therapy schemes targeted against a broad range of pathologies connected to oxidative stress and systemic inflammation.

Further work on identification of such complexes and their selective enrichment might result in new efficient and safe drugs and drug supplements. Importantly, *G. rivale* L. is widely spread and abundant on the European and North American continents and can be easily introduced into cultivation. Therefore, this plant can be considered as a potential source for isolation of biologically active complexes even on industrial scale. Thus, it is strongly mandatory to assess other types of pharmacological activities characteristic for the extracts and individual metabolites of water avens and research the possibility of their application in medical practice.

## Figures and Tables

**Table 1:** Metabolites annotated in aerial part of *Geum rivale* L. by reversed phase ultra-high-performance liquid chromatography—tandem mass spectrometry (RP-UHPLC-QqTOF-MS/MS).

#	t_R_^a^ (min)	[M−H]^−^_obs_^b^ (*m*/*z*)	[M−H]^−^_calc_^c^ (*m*/*z*)	EC ^d^	Fragmentation Patterns ^e^	Δm (ppm)	Assignment	Extract Fraction	Ref. ^f^
Phenolic acids derivatives									
1	4.2	635.0908	635.0890	C_27_H_25_O_18_	169.0125 (5%), 313.0553 (7%), 465.0676 (20%), 483.0735 (4%), 635.0908 (100%)	−2.6	Trigalloyl hexose	EA	[25,26,27]
2	4.3	457.0907	457.0988	C_19_H_21_O_13_	179.0333 (100%), 341.0802 (70%), 457.0907 (30%)	17.7	Caffeoyl-hexoside malate	A	[25]
3	5.9	300.9959	300.9999	C_14_H_6_O_8_	117.0333 (9%), 145.0282 (8%), 173.0226 (15%), 185.0230 (20%), 201.0170 (20%), 229.0110 (23%), 245.0060 (18%), 283.9932 (40%), 299.9874 (42%), 300.9959 (100%)	10	Ellagic acid	EA	[28]
4	5.7	433.0366	433.0412	C_19_H_14_O_12_	299.9888 (40%), 300.9965 (100%), 313.0684 (25%), 343.0769 (10%), 433.0359 (50%)	10.6	Ellagic acid pentoside	EA	[25]
Flavonoids and their derivatives									
5	4.2	653.0976	653.0996	C_27_H_26_O_19_	301.0343 (10%), 477.0649 (13%), 653.0976 (100%)	2.6	Quercetin-bis-hexuronide	A	[27]
6	6.3	477.0638	477.0675	C_21_H_18_O_13_	301.0376 (100%), 477.0638 (5%)	5.6	Quercetin-hexuronide	A	[25,29]
7	6.2	953.1265	953.1266	C_42_H_34_O_26_	301.0336 (5%), 477.0647 (100%), 953.1265 (70%)	−6.8	Quercetin-hexuronide dimer	EA	[30]
8	5.2	637.1003	637.1046	C_27_H_27_O_18_	285.0384 (17%), 461.0686 (45%), 637.1003 (100%)	−1.0	Kaempferol-bis-hexuronide	EA	[20]
9	6.8	923.1519	923.1523	C_42_H_35_O_24_	285.0394 (10%), 461.0708 (100%), 923.1519 (70%)	4.2	Dihydrokaempferol-kaempferol-hexuronide dimer	EA	[30]
10	9.7	285.0344	285.0405	C_15_H_10_O_6_	93.0333 (22%), 108.0190 (10%), 117.0321 (8%), 154.0374 (9%), 159.0433 (15%), 169.0626 (10%), 185.0576 (25%), 187.0360 (17%), 211.0351 (10%), 227.0295 (13%), 239.0295 (15%), 285.0344 (100%)	21.7	Kaempferol	EA	[31]
11	5.6	461.0692	461.0725	C_21_H_18_O_22_	285.0385 (100%), 461.0692 (15%)	3.2	Kaempferol-hexuronide	A	[32,33]
12	5.0	667.1106	667.1152	C_28_H_28_O_19_	315.0487 (15%), 491.0797 (30%), 667.1106 (100%)	6.8	Isorhamnetin-bis-hexuronide	A	[20]
13	5.8	491.0818	491.0831	C_22_H_20_O_13_	300.0261 (10%), 315.0501 (100%), 491.0818 (6%),	2.6	Isorhamnetin-hexuronide	A	[34,35]
Tannins									
14	0.6	481.0615	481.0624	C_20_H_19_O_14_	300.9988 (70%), 481.0606 (100%), 275.0195 (40%), 133.0143 (20%)	1.9	HHDP-hexoside	EA	[25,36,37]
15	3.5	577.1263	577.1351	C_30_H_26_O_12_	289.0673 (16%), 407.0704 (18%), 425.0803 (30%), 577.1263 (100%)	15.3	Proantocyanidin dimer	EA	[25,38]
16a	3.2	783.0634	783.0686	C_34_H_25_O_22_	300.9967 (4%), 783.0634 (100%)	6.6	Pedunculagin I	EA	[25,36,37]
16b	4.7	783.0680	783.0686	C_34_H_25_O_22_	300.9982 (100%), 633.0714 (75%), 783.0680 (100%)	0.7	Pedunculagin I	A	[25,36,37]
17	4.9	935.0780	935.0796	C_41_H_28_O_26_	300.9988 (30%), 767.0745 (2%), 935.0780 (100%)	1.7	Galloyl-bis-HHDP-hexose	A	[25,36,37]

^a^ the analytes are listed in the order of their elution; ^b^ the *m*/*z* values were derived from the acquired spectra; ^c^ the *m*/*z* values were calculated based on the predicted elemental composition (EC); ^d^ the elemental compositions were predicted with mass tolerance of 10 ppm; ^e^ MS/MS fragmentation patterns are provided as *m*/*z* values of the fragment ions (their relative intensities); ^f^ literature references providing information on the elemental compositions and MS/MS fragmentation patterns of the annotated compounds; EA- compounds were found in the ethyl acetate extract of water avens; A—compounds were found in the aqueous extract of water avens; HHDP—hexahydroxydiphenoyl group.

**Table 2:** Antioxidant activities of the aqueous and ethyl acetate fractions of the total aq. ethanolic extract prepared from the aerial part of *Geum rivale* L. assessed by DPPH, TEAC and NBT assays.

Plant Isolate	DPPH Normalized Activity, %	TEAC, μmol/L Trolox eq./μg	NBT Assay, nmol of O_2_•^−^/min
Aqueous fraction	98.23 ± 1.11	3.70 ± 0.01	3.22 ± 0.48
Ethyl acetate fraction	94.65 ± 0.29	0.03 ± 0.02	6.01 ± 0.46

DPPH—2,2-diphenyl-1-picrylhydrazyl free radical scavenging assay; TEAC—Trolox equivalent antioxidant capacity; NBT (nitroblue tetrazolium) assays—assessment of capacity to scavenge superoxide anion radicals. 10 μg of each lyophilized fraction were used.

**Table 3:** Antibacterial activities of the aqueous and ethyl acetate fractions of the total aq. ethanolic extract obtained from aerial parts of *G. rivale* L.

Microorganism Strain	Activity (MICs, µg/mL)	
	Aqueous Fraction	Ethyl Acetate Fraction
*Escherichia coli* ATCC 25922	62.5	15.6
*Pseudomonas aeruginosa* ATCC 27853	62.5	31.2
*Staphylococcus aureus* SG-511	31.2	15.6
*Staphylococcus aureus* ATCC 25923	62.5	15.6
MRSA ATCC 33591	62.5	31.2
*Micrococcus luteus* CIP A270	>250	62.5
*Listeria monocytogenes* EGD	>250	62.5

Antibacterial activities were expressed as minimal inhibitory concentrations, MICs.

**Table 4:** The contents of the individual major components in ethyl acetate and aqueous fractions of the total aqueous ethanolic extract prepared from *Geum rivale* L. aerial parts.

Major Phenolic Constituents	Contents (µg/mg d.w.)	
	Ethyl Acetate Fraction	Aqueous Fraction
Isorhamnetin-3-*O*-β-*D*-glucuronide	13.74 ± 1.22	1.40 ± 0.22
Kaempferol-3-*O*-β-*D*-glucuronide	6.33 ± 0.37	0.80 ± 0.16
Isorhamnetin-bis-3,7-*O*-β-*D*-glucuronide	0.48 ± 0.04	1.88 ± 0.07
Kaempferol-bis-3,7-*O*-β-*D*-glucuronide	0.23 ± 0.03	1.22 ± 0.16
6″-(4-Hydroxycinnamoyl)-astragalin	9.76 ± 1.75	b.l.q.
Caffeoyl malate	16.44 ± 0.38	8.25 ± 0.19
3-*O*-Methylellagic acid	39.23 ± 1.45	0.0043 ± 0.0007

Values are presented in μg/mg of the dry extract, b.l.q.—below the limit of quantification.

**Table 5:** Antioxidant activities assessed by DPPH, TEAC and NBT assays for major individual compounds isolated from the aerial parts of *G. rivale* L.

Compound	DPPH Normalized Activity, %	TEAC, μmol/L Trolox eq./μg	NBT Assay, nmol of O_2_^•−^/min
Kaempferol-3-*O*-β-*D*-glucuronide	76.452 ± 0.890	0.519 ± 0.057	7.667 ± 0.96
Isorhamnetin-bis-3,7-*O*-β-*D*-glucuronide	75.557 ± 1.784	0.611 ± 0.039	3.477 ± 0.576
3-*O*-Methylellagic acid	93.675 ± 0.890	2.963 ± 0.024	6.027 ± 0.870
6″-(4-hydroxycinnamoyl)-astragalin	80.827 ± 1.250	0.796 ± 0.024	1.813 ± 0.540
Kaempferol-bis-3,7-*O*-β-*D*-glucuronide	99.483 ± 2.512	0.815 ± 0.029	4.649 ± 0.598
Isorhamnetin-3-*O*-β-*D*-glucuronide	86.993 ± 1.867	0.019 ± 0.014	7.387 ± 0.563
Caffeoyl malate	80.469 ± 4.012	3.074 ± 0.115	7.076 ± 0.890
DMSO (negative control)	-	-	8.373 ± 0.076

DPPH—2,2-diphenyl-1-picrylhydrazyl free radical scavenging assay; TEAC—Trolox equivalent antioxidant capacity; NBT (nitroblue tetrazolium) assays—assessment of capacity to scavenge superoxide anion radicals.

**Table 6:** Antibacterial activity of individual natural products isolated from the aqueous and ethyl acetate fractions of *G. rivale* L. aerial parts.

Microorganism Strain	Minimal Inhibitory Concentrations (MICs, µg/mL)					
	11	12	3-*O*-Methylellagic Acid	8	13	Caffeoyl Malate
*Escherichia coli* ATCC 25922	500	>500	125	500	>125	500
*Pseudomonas aeruginosa* ATCC 27853	250	250	62.5	250	125	125
*Staphylococcus aureus* SG-511	500	250	31.2	250	>125	125
*Staphylococcus aureus* ATCC 25923	500	>500	62.5	>500	>125	250
*Staphylococcus aureus* MRSA ATCC 33591	500	500	250	500	>125	500
*Micrococcus luteus* CIP A270	>500	>500	250	>500	>125	>500
*Listeria monocytogenes* EGD	>500	>500	125	>500	>125	>500

The individual metabolites are labeled as in Table 1.

## Supplementary Materials

The following supporting information can be downloaded at: https://www.mdpi.com/article/10.3390/plants11212859/s1. Table S1: Chromatographic conditions used for UHPLC separation of extracts and standard compounds; Table S2: Instrument settings applied for LIT-Orbitrap-MSn experiments; Table S3: Collision energy settings applied for LIT-Orbitrap-MSn experiments; Table S4: Instrumental limits of detection (LOD), limits of quantification (LOQ) and linear dynamic ranges (LDRs) determined for the pure compounds used as calibration standards for the standard addition approach; Table S5: Amounts of the standard compounds spiked to the solutions of extracts and their concentrations used for quantification with the standard addition approach; Table S6: Purity and sufficient yields of isolated compounds from aqueous and ethyl acetate fractions of total extract from aerial part of *Geum rivale* L.; Table S7: Parameters for peak integration applied for absolute quantification of selected analytes in the ethyl acetate extract; Table S8: ^1^H NMR spectroscopic data for isorhamnetin-3-O-b-D-glucuronide (400 MHz, DMSO-*d*_6_, temperature 25 °C); Table S9: ^1^H NMR spectroscopic data for kaempferol-3-O-b-D-glucuronide (400 MHz, DMSO-*d*_6_, temperature 25 °C); Table S10: ^1^H and ^13^C NMR spectroscopic data (400 and 100 MHz, DMSO-*d*_6_, temperature 25 °C) for kaempferol-bis-3,7-O-β-D-glucuronide and isorhamnetin-bis-3,7-O-β-D-glucuronide; Table S11: ^1^H NMR spectroscopic data for 6-(4-hydroxycinnamoyl)-astragalin (400 MHz, DMSO-*d*_6_, temperature 25 °C); Table S12: ^1^H NMR spectroscopic data for caffeoyl malic acid (400 MHz, DMSO-*d*_6_, temperature 25 °C); Table S13: ^1^H NMR spectroscopic data for 3-O-methylellagic acid (400 MHz, DMSO-*d*_6_, temperature 25 °C); Figure S1: Major secondary metabolites isolated from the aqueous (S1-1) and ethyl acetate (S1-2) fractions of the total aq. ethanolic extract from the aerial parts of *Geum rivale* L.; Figure S2: Cell culture differentiation control; Figure S3: Secondary metabolites annotated by exact *m*/*z* values and SWATH-MS/MS fragmentation patterns in the total aq. ethanolic extract of G. rivale aerial parts; Figure S4: Secondary metabolites annotated by exact *m*/*z* values and targeted MS/MS fragmentation patterns in the total aq. ethanolic extract of *G. rivale* L. aerial parts; Figure S5: Results of the MTT assay addressing toxicity of the fractions of total extracts of *Geum rivale* L.; Figure S6: High-resolution electrospray ionization mass spectrum (HR-ESI-MS) with a signal of the [M−H]^−^ ion at *m*/*z* 491 corresponding to isorhamnetin-3-O-β-D-glucuronide; Figure S7: High-resolution electrospray ionization mass spectrum (HR-ESI-MS) with a signal of the [M−H]^−^ ion at *m*/*z* 461 corresponding to kaempferol-3-O-β-D-glucuronide; Figure S8: High-resolution electrospray ionization mass spectrum (HR-ESI-MS) with a signal of the [M−H]^−^ ion at *m*/*z* 667 corresponding to isorhamnetin-bis-3,7-O-β-D-glucuronide; Figure S9: High-resolution electrospray ionization mass spectrum (HR-ESI-MS) with a signal of the [M−H]^−^ ion at *m*/*z* 637 corresponding to kaempferol-bis-3,7-O-β-D-glucuronide; Figure S10: High-resolution electrospray ionization mass spectrum (HR-ESI-MS) with a signal of the [M−H]^−^ ion at *m*/*z* 593 corresponding to 6″-(4-hydroxycinnamoyl)-astragalin; Figure S11: High-resolution electrospray ionization mass spectrum (HR-ESI-MS) with a signal of the [M−H]^−^ ion at *m*/*z* 295 corresponding to caffeoyl malate; Figure S12: High-resolution electrospray ionization mass spectrum (HR-ESI-MS) of the [M−H]^−^ ion at *m*/*z* 315 corresponding to 3-O-methylellagic acid; Scheme S1: Proposed tandem mass spectrometric fragmentation patterns for the ion at *m*/*z* 491 ([M−H]^−^) corresponding to isorhamnetin-3-O-β-D-glucuronide; Scheme S2: Proposed tandem mass spectrometric fragmentation patterns for *m*/*z* 461 ([M−H]^−^) corresponding to kaempferol-3-O-β-D-glucuronide; Scheme S3: Proposed tandem mass spectrometric fragmentation patterns for the ion at *m*/*z* 667 ([M−H]^−^, MS2, red) corresponding to isorhamnetin-bis-3,7-O-β-D-glucuronide; Scheme S4: Proposed tandem mass spectrometric fragmentation patterns for the ion at *m*/*z* 637 ([M−H]^−^) corresponding to kaempferol-bis-3,7-O-β-D-glucuronide; Scheme S5: Proposed tandem mass spectrometric fragmentation patterns for the ion at *m*/*z* 593 ([M−H]^−^, MS2, red) corresponding to 6″-(4-hydroxycinnamoyl)-astragalin; Scheme S6: Proposed tandem mass spectrometric fragmentation patterns for the ion at *m*/*z* 295 ([M−H]^−^) corresponding to caffeoyl malate; Scheme S7: roposed tandem mass spectrometric fragmentation patterns for the ion at *m*/*z* 315 ([M−H]^−^, MS2, red) corresponding to 3-O-methylellagic acid.
