# Supplementary material for: Phytochemical Characterization of Water Avens (Geum rivale L.) Extracts: Structure Assignment and Biological Activity of the Major Phenolic Constituents
Source: Plants (Basel). 2022 Oct 26;11(21):2859. doi: 10.3390/plants11212859 (PMC9658556; doi:10.3390/plants11212859)
Supplement: Supplementary file 1 [file plants-11-02859-s001.zip › plants-1972313-supplementary.pdf]

Phytochemical characterization of water avens (*Geum rivale* L.) extracts: structure assignment and biological activity of major phenolics constituents

Supplementary information

Directory

Tables

Table S1 Chromatographic conditions used for UHPLC separation of extracts and standard compounds 5

Table S2 Instrument settings applied for LIT-Orbitrap-MS^n experiments 7

Table S3 Collision energy settings applied for LIT-Orbitrap-MS^n experiments 9

Table S4 Instrumental limits of detection (LOD), limits of quantification (LOQ) and linear dynamic ranges (LDRs) determined for the pure compounds used as calibration standards for the standard addition approach 10

Table S5 Amounts of the standard compounds spiked to the solutions of extracts and their concentrations used for quantification with the standard addition approach 11

Table S6 Purity and sufficient yields of isolated compounds from aqueous and ethyl acetate fractions of total extract from aerial part of *Geum rivale* L. 12

Table S7 Parameters for peak integration applied for absolute quantification of selected analytes in the ethyl acetate extract 13

Table S8 1H NMR spectroscopic data for isorhamnetin-3-*O*-b-*D*-glucuronide (400 MHz, DMSO-*d6*, temperature 25°C) 14

Table S9 1H NMR spectroscopic data for kaempferol-3-*O*-b-*D*-glucuronide (400 MHz, DMSO-*d6*, temperature 25°C) 15

Table S10 1H and 13C NMR spectroscopic data (400 and 100 MHz, DMSO-*d6*, temperature 25°C) for kaempferol-bis-3,7-*O*-β-D-glucuronide and isorhamnetin-bis-3,7-*O*-β-D-glucuronide 16

Table S11 1H NMR spectroscopic data for 6-(4-hydroxycinnamoyl)-astragalin (400 MHz, DMSO-*d6*, temperature 25°C) 18

Table S12 1H NMR spectroscopic data for caffeoyl malic acid (400 MHz, DMSO-*d6*, temperature 25°C).. 19

Table S13 1H NMR spectroscopic data for 3-*O*-methylellagic acid (400 MHz, DMSO-*d6*, temperature 25°C) 20

Figures

Figure S1. Major secondary metabolites isolated from the aqueous (S1-1) and ethyl acetate (S1-2) fractions of the total aq. ethanolic extract from the aerial parts of *Geum rivale* L........................... 21

Figure S2. Cell culture differentiation control... 23

Figure S3. Secondary metabolites annotated by exact *m/z* values and SWATH-MS/MS fragmentation patterns in the total aq. ethanolic extract of *G. rivale* aerial parts. 24

Figure S4 Secondary metabolites annotated by exact *m/z* values and targeted MS/MS fragmentation patterns in the total aq. ethanolic extract of *G. rivale* L. aerial parts. 30

Figure S5 Results of the MTT assay addressing toxicity of the fractions of total extracts of *Geum rivale* L.. 46

Figure S6. High-resolution electrospray ionization mass spectrum (HR-ESI-MS) with a signal of the $[M-H]^-$ ion at *m/z* 491 corresponding to isorhamnetin-3-*O*-β-D-glucuronide................................. 47

Figure S7. High-resolution electrospray ionization mass spectrum (HR-ESI-MS) with a signal of the $[M-H]^-$ ion at *m/z* 461 corresponding to kaempferol-3-*O*-β-D-glucuronide. 51

Figure S8. High-resolution electrospray ionization mass spectrum (HR-ESI-MS) with a signal of the $[M-H]^-$ ion at *m/z* 667 corresponding to isorhamnetin-bis-3,7-*O*-β-D-glucuronide......................... 54

Figure S9. High-resolution electrospray ionization mass spectrum (HR-ESI-MS) with a signal of the $[M-H]^-$ ion at *m/z* 637 corresponding to kaempferol-bis-3,7-*O*-β-D-glucuronide. 58

Figure S10. High-resolution electrospray ionization mass spectrum (HR-ESI-MS) with a signal of the $[M-H]^-$ ion at *m/z* 593 corresponding to 6''-(4-hydroxycinnamoyl)-astragalin........................... 62

Figure S11. High-resolution electrospray ionization mass spectrum (HR-ESI-MS) with a signal of the [M-H]$^-$ ion at *m/z* 295 corresponding to caffeoyl malate.. 66

Figure S12. High-resolution electrospray ionization mass spectrum (HR-ESI-MS) of the [M-H]$^-$ ion at *m/z* 315 corresponding to 3-*O*-methylellagic acid... 70

Schemes

Scheme S1. Proposed tandem mass spectrometric fragmentation patterns for the ion at *m/z* 491 ([M-H]$^-$) corresponding to isorhamnetin-3-*O*-β-D-glucuronide 73

Scheme S2. Proposed tandem mass spectrometric fragmentation patterns for *m/z* 461([M-H]$^-$) corresponding to kaempferol-3-*O*-β-D-glucuronide 74

Scheme S3. Proposed tandem mass spectrometric fragmentation patterns for the ion at *m/z* 667 ([M-H]$^-$, MS^2, red) corresponding to isorhamnetin-bis-3,7-*O*-β-D-glucuronide 75

Scheme S4. Proposed tandem mass spectrometric fragmentation patterns for the ion at *m/z* 637 ([M-H]$^-$) corresponding to kaempferol-bis-3,7-*O*-β-D-glucuronide 76

Scheme S5. Proposed tandem mass spectrometric fragmentation patterns for the ion at *m/z* 593 ([M-H]$^-$, MS^2, red) corresponding to 6''-(4-hydroxycinnamoyl)-astragalin... 77

Scheme S6. Proposed tandem mass spectrometric fragmentation patterns for the ion at *m/z* 295 ([M-H]$^-$) corresponding to caffeoyl malate 78

Scheme S7. Proposed tandem mass spectrometric fragmentation patterns for the ion at *m/z* 315 ([M-H]$^-$, MS^2, red) corresponding to 3-*O*-methylellagic acid... 79

References 80

Tables

Table S1 Chromatographic conditions used for UHPLC separation of extracts and standard compounds

Parameter	Settings
**Column**	
Column	Nucleoshell C18, 150 mm x 2 mm, particle size 2.7 µm
Column temperature	40°C
**Eluents**	
Eluent A	0.3 mmol/L aq. ammonium formate
Eluent B	acetonitrile
Flow rate	0.4 mL/min
**Injection**	
Injection mode	Partial Loop
Injection volume	5 µL
Wash solvent (Dionex)	methanol
Weak wash solvent (Acquity)	0.3 mmol/L aq. ammonium formate
Strong wash solvent (Acquity)	acetonitrile

Wash volume (Dionex)	2000 µL
Weak wash volume (Acquity)	600 µL
Strong wash volume (Acquity)	200 µL
Sample temperature	5°C

Separation gradient

**Time (min)**	**% eluent B**
Initial	5
2.00	Isocratic at 5
19.00	linear gradient from 5 to 95
21.00	isocratic at 95
21.01	linear gradient from 95 to 5
28.00	isocratic at 5 (re-equilibration)

Table S2 Instrument settings applied for LIT-Orbitrap-MS^n experiments

Parameter	Setting
**MS conditions**	
Mass analyzer type	hybrid linear ion trap-orbital trap mass spectrometer (LIT-Orbitrap-MS)
Ionization mode	Negative
Scan type	Full scan
Data type	Profile
Resolution	30000
Ion spray voltage	3800 V
Source current	100 µA
Auxiliary gas flow	5 arbitrary units
Sheath gas flow	18 arbitrary units
Sweep gas flow	0 arbitrary units
Capillary temperature	275°C
Source heater temperature	300°C
S-lens RF level	65.7%
Mass to charge ratio (*m/z*) range	50 – 1250
**MS^n conditions**	
Fragmentation	CID /HCD
Normalized collision energy	Compound dependent (more details in Table S2)
Resolution	15000
Isolation width	2 Da

Activation frequency	0.25
Activation time	10 ms / 0.1 ms
HCD charge state	1

CID, collision-induced dissociation via excitation in the linear ion trap (LIT); HCD, higher-energy C-trap dissociation – collision-activated dissociation in a multipole

Table S3 Collision energy settings applied for LIT-Orbitrap-MS^n experiments

Compound	Normalized collision energy
3-*O*-Methylellagic acid	MS^2[315.0]$^-$, CID, 30%;<br>MS^3[299.0]$^-$, CID, 35%
Caffeoyl malate	MS^2[295.0]$^-$, CID, 25%;<br>MS^3[133.0]$^-$, CID, 35%;<br>MS^3[179.0]$^-$, CID, 35%
6”-(4-Hydroxycinnamoyl)-astragalin	MS^2[593.0]$^-$, CID, 30%;<br>MS^3[285.0]$^-$, CID, 35%;<br>MS^3[307.0]$^-$, CID, 30%
Kaempferol-3-*O*-β-D-glucuronide	MS^2[461.0]$^-$, CID, 35%;<br>MS^3[285.0]$^-$, CID, 35%
Kaempferol-bis-3,7-*O*-β-D-glucuronide	MS^2[637.0]$^-$, CID, 30%;<br>MS^3[461.0]$^-$, CID, 35%;<br>MS^4[285.0]$^-$, CID, 45%
Isorhamnetin-3-*O*-β-D-glucuronide	MS^2[491.0]$^-$, CID, 35%;<br>MS^3[315.0]$^-$, CID, 35%,<br>MS^4[300.0]$^-$, HCD, 85%
Isorhamnetin-bis-3,7-*O*-β-D-glucuronide	MS^2[667.0]$^-$, CID, 30%;<br>MS^3[315.0]$^-$, CID, 35%,<br>MS^5[300.0]$^-$, HCD, 85%

Table S4 Instrumental limits of detection (LOD), limits of quantification (LOQ) and linear dynamic ranges (LDRs) determined for the pure compounds used as calibration standards for the standard addition approach

**Compound**	**LOD (fmol)**	**LOQ (fmol)**	**LDR**	**slope**	**intercept**	**R^2**
Isorhamnetin-3-*O*-β-D-glucuronide	10	10	1.00E+03	55447	16175	0.9983
Kaempferol-3-*O*-β-D-glucuronide	2.5	5	2.00E+04	164139	66666	0.9966
Isorhamnetin-bis-3,7-*O*-β-D-glucuronide	10	25	4.00E+03	44091	29586	0.9948
Kaempferol-bis-3,7-*O*-β-D-glucuronide	2.5	5	2.00E+03	397641	3725	0.9922
6"-(4-Hydroxycinnamoyl)astragalin	2.5	5	1.00E+04	660610	489904	0.9918
Caffeoyl malate	5	100	1.00E+03	21031	-978.26	0.9957
3-*O*-Methylellagic acid	250	500	1.00E+03	10863	14128	0.9973

Table S5 Amounts of the standard compounds spiked to the solutions of extracts and their concentrations used for quantification with the standard addition approach

	**Ethyl acetate**		**Water**	
	**pmol on column**	**mg/mL of the extract**	**pmol on column**	**mg/mL of the extract**
Isorhamnetin-3-*O*-β-D-glucuronide	1.875, 3.75, 11.25, 22.5, 45	0.05	0.3125, 0.625, 1.875, 3.75, 11.25	0.05
Kaempferol-3-*O*-β-D-glucuronide	0.3125, 0.625, 1.875, 3.75, 11.25	0.05	0.3125, 0.625, 1.875, 3.75, 11.25	0.05
Isorhamnetin-bis-3,7-*O*-β-D-glucuronide	0.3125, 0.625, 1.875, 3.75, 11.25	0.25	0.3125, 0.625, 1.875, 3.75, 11.25	0.05
Kaempferol-bis-3,7-*O*-β-D-glucuronide	0.3125, 0.625, 1.875, 3.75, 11.25, 22.5	0.25	0.3125, 0.625, 1.875, 3.75, 11.25	0.05
6"-(4-Hydroxycinnamoyl)astragalin	0.3125, 0.625, 1.875, 3.75, 11.25	0.005	0.3125, 0.625, 1.875, 3.75, 11.25	5
Caffeoyl malate	3.125, 6.25, 18.75, 37.5, 112.5	0.05	3.125, 6.25, 18.75, 37.5, 112.5	0.05
3-*O*-Methylellagic acid	3.125, 6.25, 18.75, 37.5, 112.5	0.05	3.125, 6.25, 18.75, 37.5, 112.5	5

The defined on-column amounts were injected the UHPLC in the volume of 5 µL.

Table S6 Purity and sufficient yields of isolated compounds from aqueous and ethyl acetate fractions of total extract from aerial part of *Geum rivale* L.

**Compound**	**Purity, %**	**Sufficient yields, %**
3-*O*-Methylellagic acid	96.54	0.082
Caffeoyl malate	91.23	0.036
6”-(4-Hydroxycinnamoyl)-astragalin	93.14	0.060
Kaempferol-3-*O*-β-D-glucuronide	92.76	0.158
Kaempferol-bis-3,7-*O*-β-D-glucuronide	92.35	0.123
Isorhamnetin-3-*O*-β-D-glucuronide	90.44	0.011
Isorhamnetin-bis-3,7-*O*-β-D-glucuronide	95.29	0.069

Table S7 Parameters for peak integration applied for absolute quantification of selected analytes in the ethyl acetate extract

**Authentic standard**	**[M-H]⁻ *m/z* (calculated)**	**Integration *m/z* range**	**t_R (min, ethyl acetate fraction)**
Isorhamnetin-3-*O*-b-D-glucuronide	491.0831	491.06 - 491.10	6.4
Kaempferol-3-*O*-b-D-glucuronide	461.0725	461.05 - 461.09	6.2
Isorhamnetin-bis-3,7-*O*-β-D-glucuronide	667.1152	667.09 - 667.13	4.5
Kaempferol-bis-3,7-*O*-β-D-glucuronide	637.1046	637.08 - 637.12	4.3
6-(4-Hydroxycinnamoyl)astragalin	593.1301	593.12-593.15	7. 9
Caffeoyl malate	295.0459	295.02 - 295.06	4.2
3-*O*-Methylellagic acid	315.0146	314.99 - 315.03	7.0

t_R (min) are given for the ethyl acetate fraction

Table S8 1H NMR spectroscopic data for isorhamnetin-3-*O*-β-*D*-glucuronide (400 MHz, DMSO-*d6*, temperature 25°C)

**Position**	**δH, (J in Hz)**
6-CH	6.55 (1H, d 1.9 Hz)
8-CH	6.32 (1H, d 1.9 Hz)
2’,6’-CH	8.00 (2H, d 8.8 Hz)
5’-CH	6.94 (2H, d 8.8 Hz)
1’’-CH	5.48 (1H, d 7.5 Hz)
2’’-CH	3.37 (1H, t 9.7, 8.8 Hz)
3’’-CH	3.28 (1H, t 9.2, 8.8 Hz)
4’’-CH	3.22 (2H, m)
5’’-CH	3.59 (2H, d 9.7 Hz)
5-OH	12.49 (1H, s)
7-OH	11.38 (1H, s)
4’-OH	10.57 (1H, s)
3’-OMe	3.70 (3H, s)

Table S9 1H NMR spectroscopic data for kaempferol-3-*O*-β-*D*-glucuronide (400 MHz, DMSO-*d6*, temperature 25°C)

**Position**	***δ*H, (J in Hz)**
6-CH	6.55 (1H, d 1.9 Hz)
8-CH	6.32 (1H, d 1.9 Hz)
2’,6’-CH	8.00 (2H, d 8.8 Hz)
3’,5’-CH	6.94 (2H, d 8.8 Hz)
1’’-CH	5.48 (1H, d 7.5 Hz)
2’’-CH	3.37 (1H, t 9.7, 8.8 Hz)
3’’-CH	3.28 (1H, t 9.2, 8.8 Hz)
4’’-CH	3.22 (2H, m)
5’’-CH	3.59 (2H, d 9.7 Hz)
5-OH	12.49 (1H, s)
7-OH	11.38 (1H, s)
4’-OH	10.57 (1H, s)

Table S10 1H and 13C NMR spectroscopic data (400 and 100 MHz, DMSO-*d6*, temperature 25°C) for kaempferol-bis-3,7-*O*-β-D-glucuronide and isorhamnetin-bis-3,7-*O*-β-D-glucuronide

	**Kaempferol-bis-3,7-*O*-β-D-glucuronide**		**Isorhamnetin-bis-3,7-*O*-β-D-glucuronide**	
**Position**	***δ*H, (J in Hz)**	***δ*C**	***δ*H, (J in Hz)**	***δ*C**
2		157.4		157.4
3		133.7		133.4
4		177.8		177.7
4a		106.3		106.3
5		161.4		161.3
6	6.48 (d, 2.0)	99.7	6.48 (d, 2.2)	99.7
7		163.0		163.0
8	6.84 (d, 2.0)	95.0	6.85 (d, 2.2)	95.2
8a		156.4		156.4
1'	-	115.9		120.8
2'	8.06 (d, 8.9)	120.8	7.95 (d, 2.0)	113.8
3'	6.94 (d, 8.9)	131.3		147.5
4'	-	160.9		150.6
5'	6.94 (d, 8.9)	131.3	7.03 (d, 8.3)	113.8
6'	8.06 (d, 8.9)	120.8	7.53 (dd, 8.3, 2.0)	122.7
1''	5.49 (d, 7.3)	101.6	5.60 (d, 7.3)	101.3
2''	3.26 (m)	76.5	3.29 (m)	76.5
3''	3.30 (m)	71.8	3.32 (m)	72.0

4''	3.39 (m)	74.4	3.36 (m)	74.7
5''	3.61 (d, 9.5)	76.0	3.65 (d, 9.2)	76.2
6''	-	170.3	-	170.3
1'''	5.27 (d, 7.6)	99.7	5.27 (d, 7.6)	99.7
2'''	3.30 (m)	75.8	3.29 (m)	76.5
3'''	3.37 (m)	71.8	3.37 (m)	71.8
4'''	3.41 (m)	74.4	3.40 (m)	74.6
5'''	4.06 (d, 9.3)	76.0	4.05 (d, 9.2)	76.0
6'''	-	170.3	-	170.5
OH-5	12.54 (brs)	-	12.53 (brs)	-
OMe-3'	-	-	3.86 (s)	55.9

Table S11 1H NMR spectroscopic data for 6-(4-hydroxycinnamoyl)-astragalin (400 MHz, DMSO-*d6*, temperature 25°C)

Position	δH, (J in Hz)
6-CH	6.49 (1H, d 2.0 Hz)
8-CH	6.23 (1H, d 2.0 Hz)
2’,6’-CH	7.96 (2H, d 8.9 Hz)
3’,5’-CH	6.91 (2H, d 8.9 Hz)
1’’-CH	5.45 (1H, d 7.7 Hz)
2’’- 5’’ -CH	3.24-3.40 (4H, m)
6’’a-CH	4.27 (1H, dd 10.3, 4.2 Hz)
6’’b-CH	4.02 (1H, dd 10.3, 6.4 Hz)
2’’’,6’’’-CH	7.36 (2H, d 8.6 Hz)
3’’’,5’’’-CH	6.85 (2H, d 8.6 Hz)
7’’’-H	6.09 (1H, d 15.8 Hz)
8’’’-H	7.32 (1H, d 15.8 Hz)
5-OH	12.55 (1H, s)
7-OH	11.25 (1H, s)
4’-OH	10.47 (1H, brs)
4’’’-OH	10.31 (1H, brs)

Table S12 1H NMR spectroscopic data for caffeoyl malic acid (400 MHz, DMSO-*d6*, temperature 25°C)

**Position**	***δ*H, (J in Hz)**
1-CH	5.29 (1H, dd 8.8, 4.0 Hz)
2a-CH	2.87 (1H, dd 16.7, 4.0 Hz)
2b-CH	2.78 (1H, dd 16.7, 8.8 Hz)
2’-CH	7.14 (1H, d 2.0 Hz)
5’-CH	6.84 (1H, d 8.2 Hz)
6’-CH	7.00 (1H, dd 8.2, 2.0 Hz)
7’-CH	7.48 (1H, d 15.8 Hz)
8’-CH	6.27 (1H, d 15.8 Hz)
3’-OH	-
4’-OH	-

Table S13 1H NMR spectroscopic data for 3-*O*-methylellagic acid (400 MHz, DMSO-*d6*, temperature 25°C)

**Position**	**δH, (J в Гц)**
1-CH	7.78 (1H, s)
6-CH	7.68 (1H, s)
8-OMe	4.0 (3H, s)
2-OH	11.26 (1H, brs)
3-OH	10.72 (1H, brs)
7-OH	11.22 (1H, brs)

Figures

Figure S1. Major secondary metabolites isolated from the aqueous (S1-1) and ethyl acetate (S1-2) fractions of the total aq. ethanolic extract from the aerial parts of *Geum rivale* L. The signals of the corresponding metabolites are marked in the UV-chromatograms (280 nm). The analysis relied on RP-UHPLC-QqTOF-MS accomplished with a Waters ACQUITY I-Class UPLC System (Waters GmbH, Eschborn, Germany) coupled on-line to a Triple-TOF6600 hybrid mass spectrometer (Sciex, Darmstadt, Germany) in the negative SWATH mode.

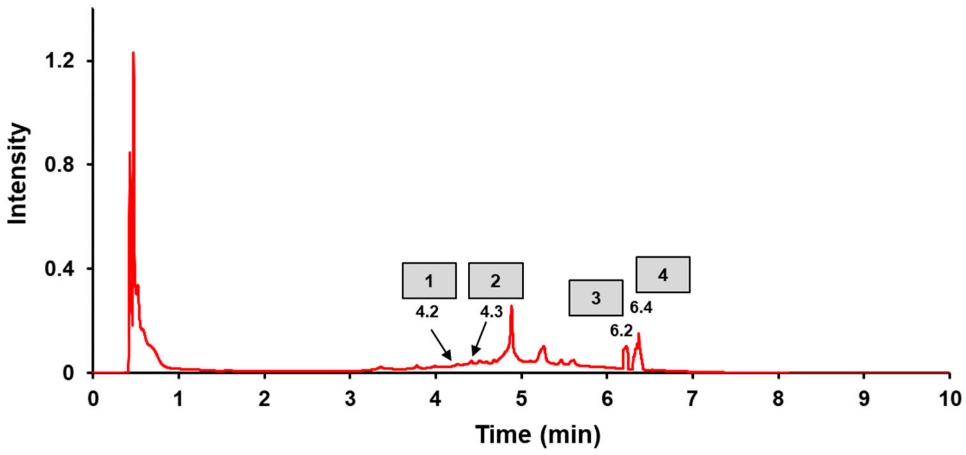

Figure S1-1. UV-chromatogram (280 nm) of the aqueous fraction of the total aq. ethanolic extract from the aerial parts of *Geum rivale* L.: **1** – caffeoyl malate; **2** - kaempferol-bis-3,7-*O*-β-D-glucuronide; **3** - kaempferol-3-*O*-β-D-glucuronide; **4** - isorhamnetin-3-*O*-β-D-glucuronide.

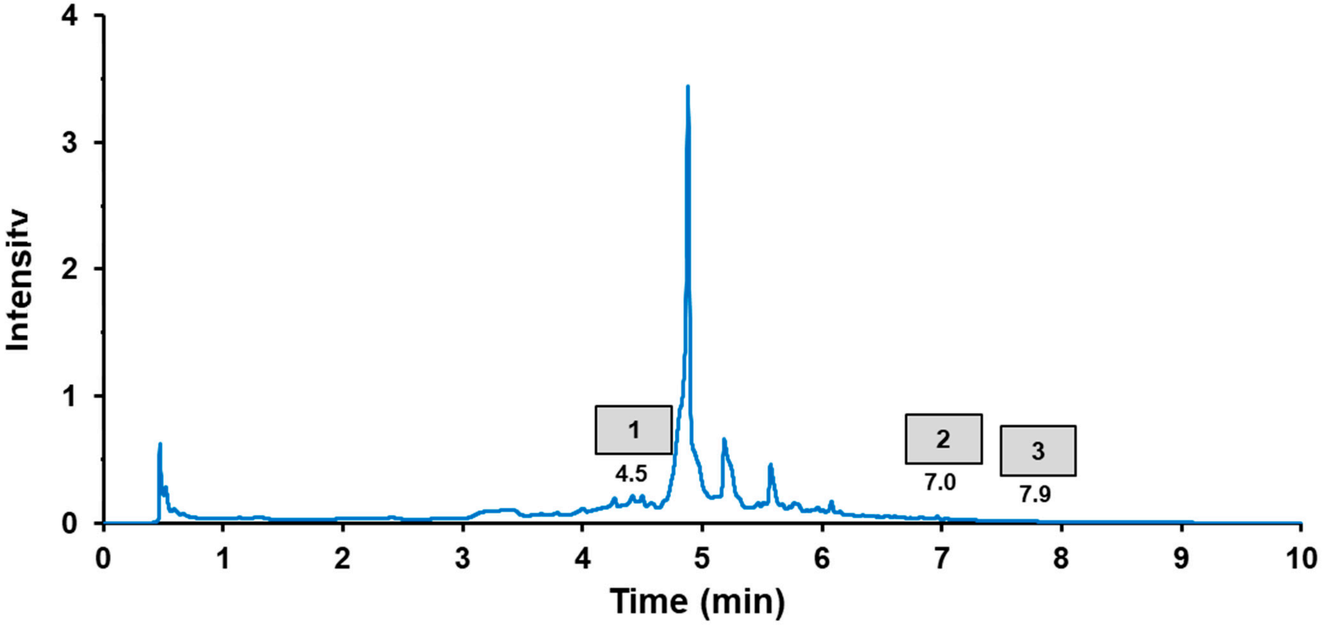

Figure S1-2. UV-chromatogram (280 nm) of the ethyl acetate fraction of the total aq. ethanolic extract from the aerial parts of *Geum rivale* L.: **1** - isorhamnetin-bis-3,7-*O*-β-D-glucuronide; **2** - 3-*O*-methylellagic acid; **3** - 6''-(4-hydroxycinnamoyl)-astragalin.

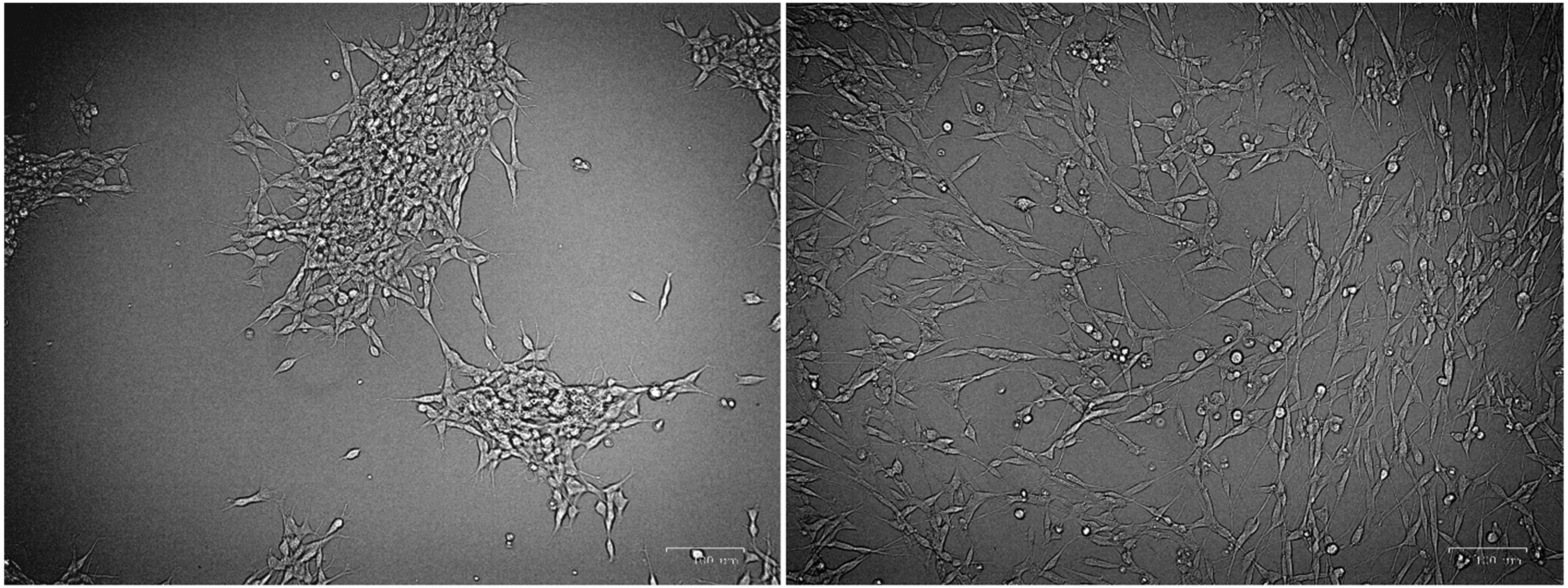

a b

Figure S2. Cell culture differentiation control: a) Undifferentiated culture of SH-SY5Y: cells with short neurites form clusters and clumps; b) Differentiated culture of SH-SY5Y: cells spindle and protrude the neuritis, resulting in interconnected monolayer.

Figures S3. Secondary metabolites annotated by exact *m/z* values and SWATH-MS/MS fragmentation patterns in the total aq. ethanolic extract of *G. rivale* aerial parts. The signals of the corresponding metabolites are marked in the total ion chromatograms (TICs) acquired for aqueous (red) and ethyl acetate (blue) fractions of the total extract of water avens (Figure S3-1). The analysis relied on RP-UHPLC-QqTOF-MS accomplished with a Waters ACQUITY I-Class UPLC System (Waters GmbH, Eschborn, Germany) coupled on-line to a Triple-TOF6600 hybrid mass spectrometer (Sciex, Darmstadt, Germany) in the negative SWATH mode. The MS/MS spectra of individual metabolites are listed as Figure S3-2 – S3-6.

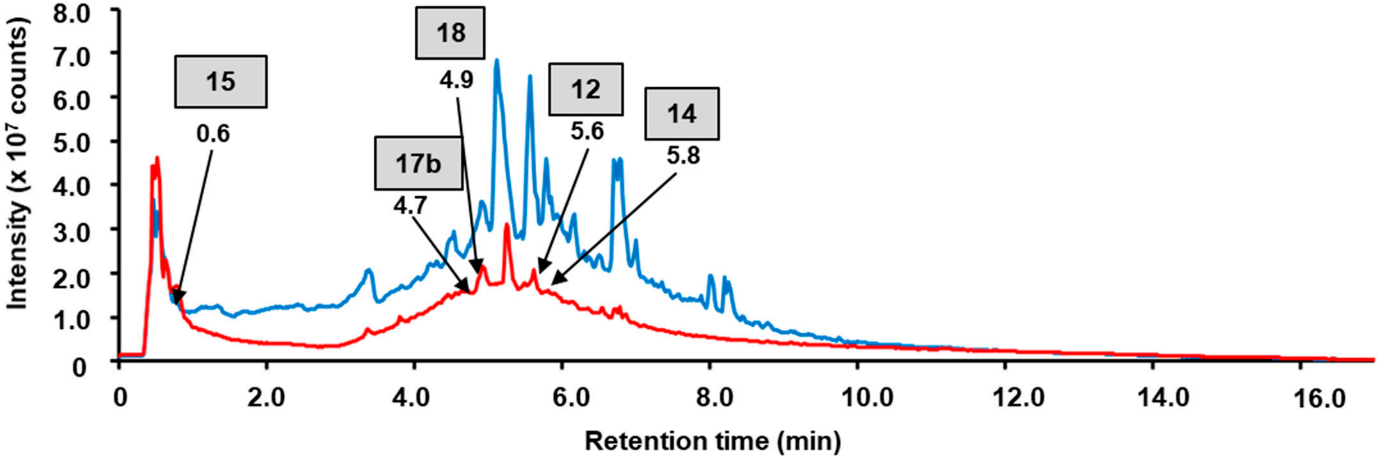

Figure S3-1 Total ion chromatograms (TICs) acquired for aqueous (red) and ethyl acetate (blue) fractions of the total extract of water avens. Signals of the metabolites annotated by the exact *m/z* and SWATH-MS/MS are marked.

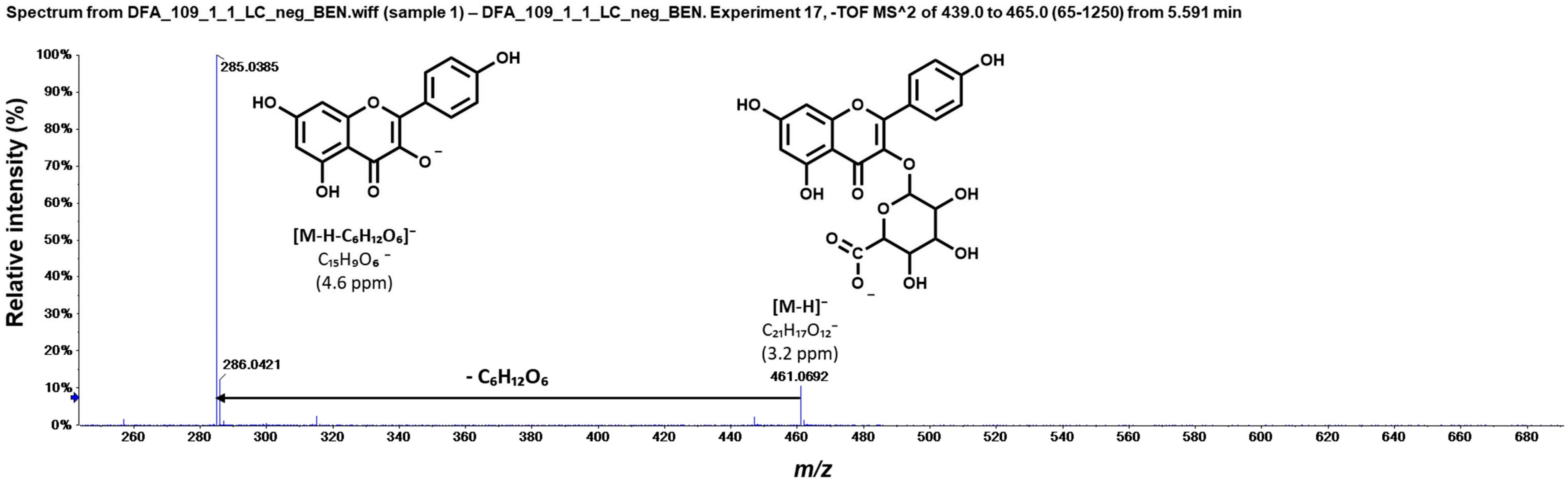

Figure S3-2. Tandem mass spectrum of *m/z* 461.1 at t_R 5.6 min corresponding to kaempferol-hexuronide (**11**), annotated in the aqueous fraction of the total aq. ethanolic extract of aerial parts of *G. rivale* L. The spectrum was acquired with a hybrid QqTOF mass spectrometer operated in the negative SWATH mode (*m/z* window 439.0-465.0) [1]. Structures shown are suggestions.

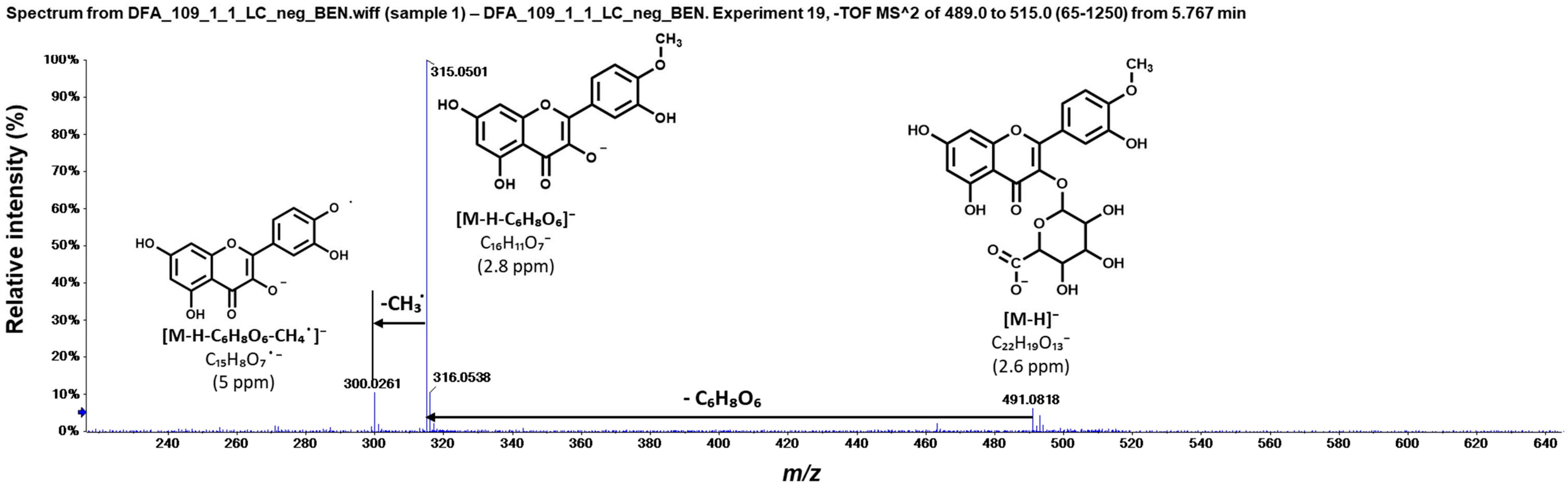

Figure S3-3. Tandem mass spectrum of *m/z* 491.1 at t_R 5.8 min corresponding to isorhamnetin-hexuronide (**13**), annotated in the aqueous fraction of the aq. ethanolic total extract of aerial parts of *G. rivale* L. The spectrum was acquired with a hybrid QqTOF mass spectrometer operated in the negative SWATH mode (*m/z* window 489.0-515.0) [2]. Structures shown are suggestions.

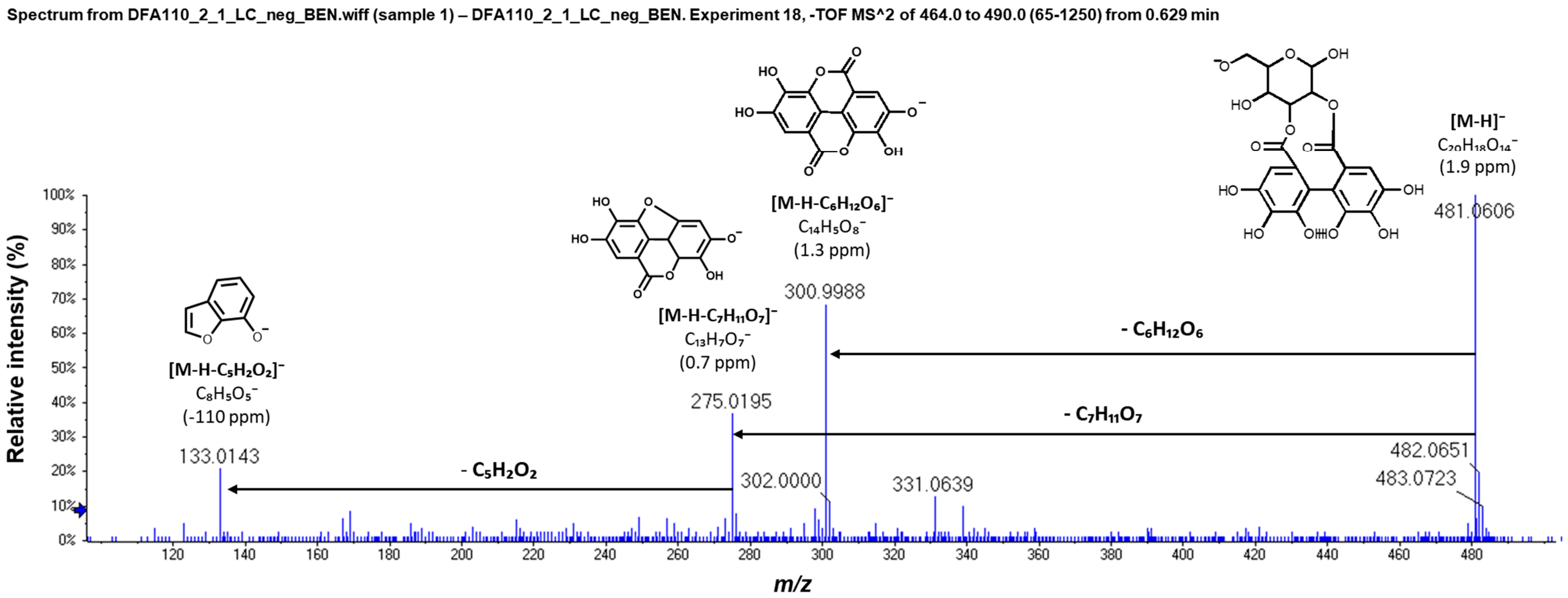

Figure S3-4 Tandem mass spectrum of *m/z* 481.1 at t_R 0.6 min corresponding to HHDP-hexoside (**14**), annotated in the ethyl acetate fraction of the total aq. ethanolic extract of aerial parts of *G. rivale* L. The spectrum was acquired with a hybrid QqTOF mass spectrometer operated in the negative SWATH mode (*m/z* window 464.0-490.0) [3]. Structures shown are suggestions.

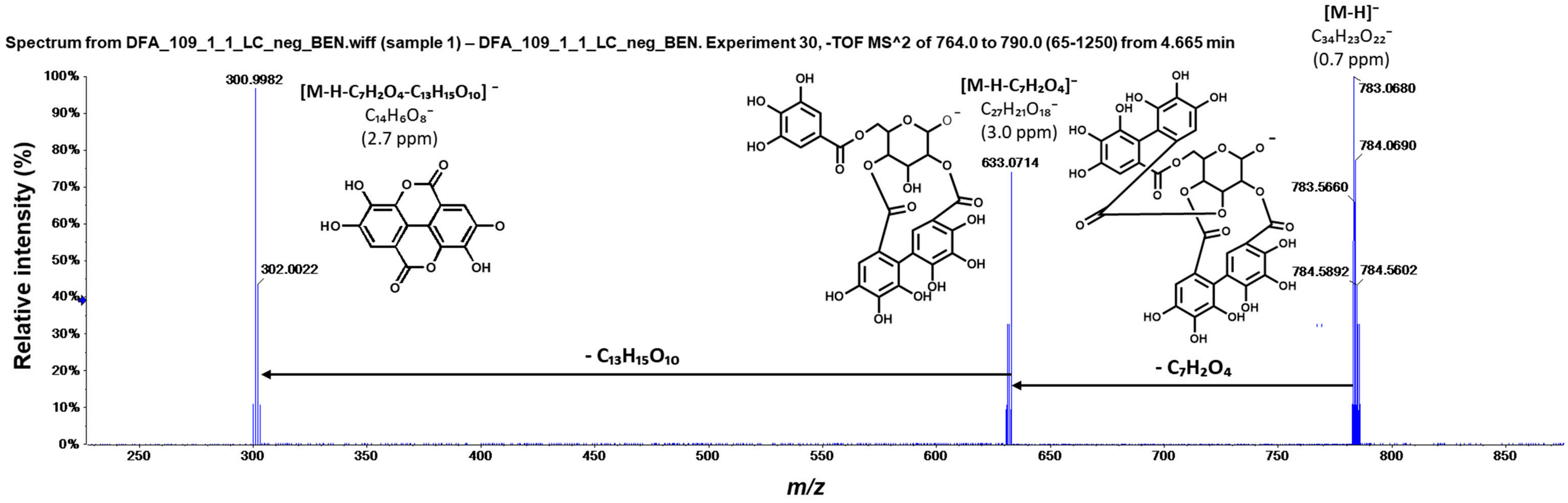

Figure S3-5. Tandem mass spectrum of *m/z* 783.1 at t_R 4.7 min corresponding to bis-HHDP-hexose (**16b**), annotated in the aqueous fraction of the total aq. ethanolic extract of the aerial parts of *G. rivale* L. The spectrum was acquired with a hybrid QqTOF mass spectrometer operated in the negative SWATH mode (*m/z* window 764.0-790.0) [3]. Structures shown are suggestions.

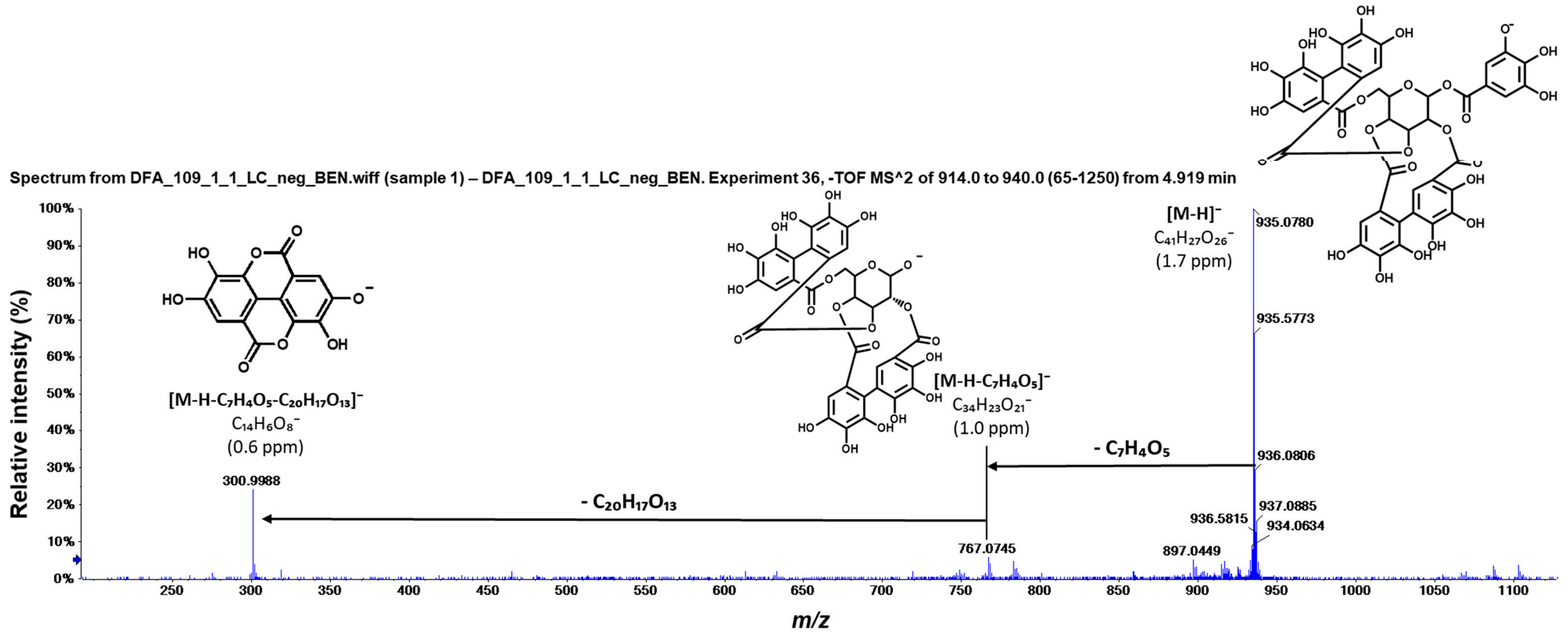

Figure S3-6. Tandem mass spectrum of *m/z* 935.1 at t_R 4.9 min corresponding to galloyl-bis-HHDP-hexose (**17**) annotated in the aqueous fraction of the total aq. ethanolic extract of the aerial parts of *G. rivale* L. The spectrum was acquired with a hybrid QqTOF mass spectrometer operated in the negative SWATH mode (*m/z* window 914.0-940.0) [3]. Structures shown are suggestions.

Figures S4 Secondary metabolites annotated by exact *m/z* values and targeted MS/MS fragmentation patterns in the total aq. ethanolic extract of *G. rivale* L. aerial parts. The signals of the corresponding metabolites are marked in the total ionchromatograms (TICs) acquired for aqueous (red) and ethyl acetate (blue) fractions of the total extract of water avens (Figure S4-1). The analysis relied on RP-UHPLC-QqTOF-MS accomplished with a Waters ACQUITY I-Class UPLC System (Waters GmbH, Eschborn, Germany) coupled on-line to a Triple-TOF6600 hybrid mass spectrometer (Sciex, Darmstadt, Germany) in the negative ion mode. The MS/MS spectra of individual metabolites are listed as Figure S4-2 – S4-16.

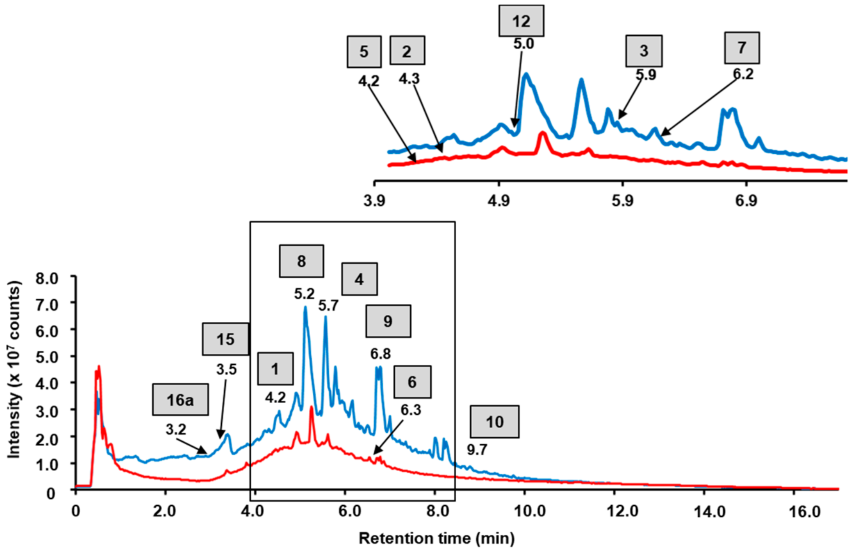

Figure S4-1 Total ion chromatograms (TICs) acquired for aqueous (red) and ethyl acetate (blue) fractions of the total extract of water avens. Signals of the metabolites annotated by the exact *m/z* and targeted MS/MS are marked

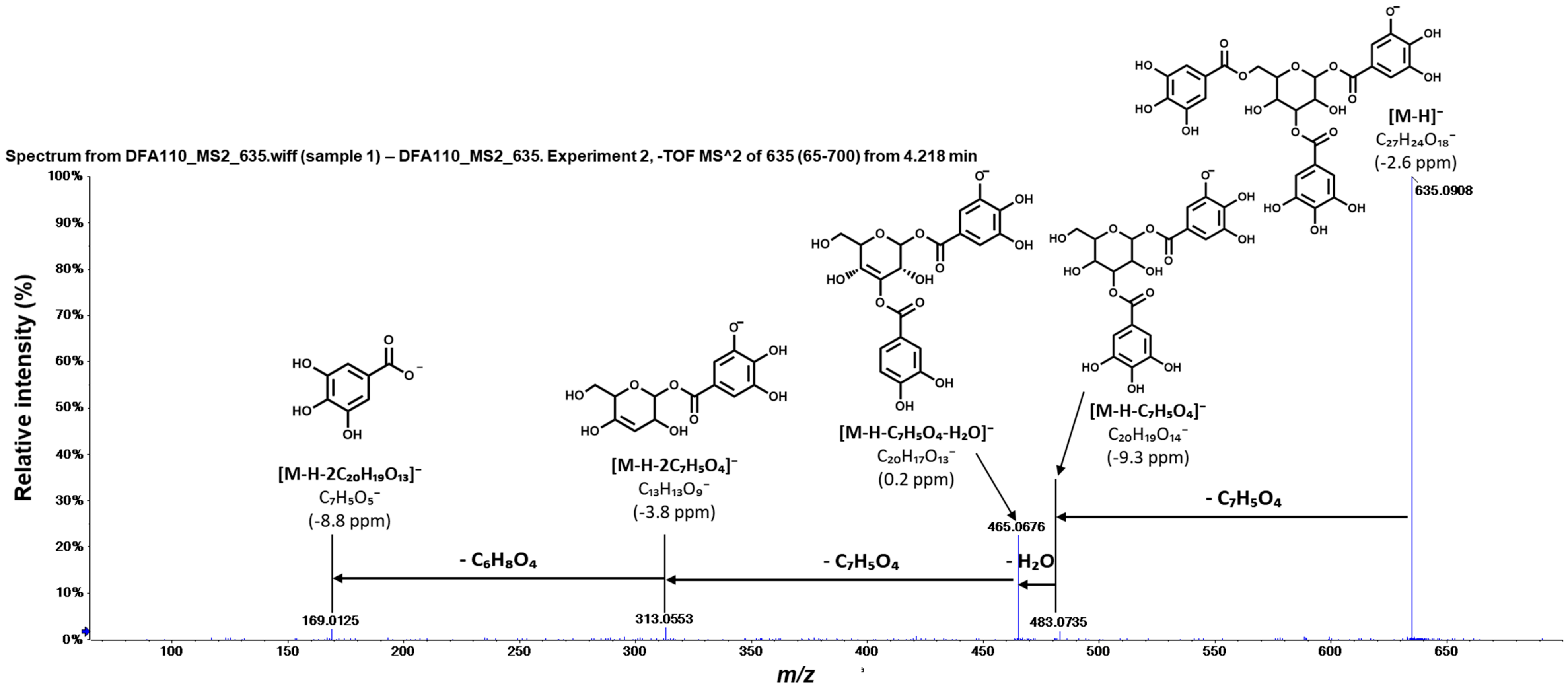

Figure S4-2. Tandem mass spectrum of *m/z* 635.1 at t_R 4.2 min corresponding to trigalloyl hexose (**1**), annotated in the ethyl acetate fraction of the total aq. ethanolic extract of aerial parts of *G. rivale* L. The spectrum was acquired with a hybrid QqTOF mass spectrometer operated in the negative product ion mode with unit Q1 resolution (collision energy 20 eV) [3]. Structures shown are suggestions.

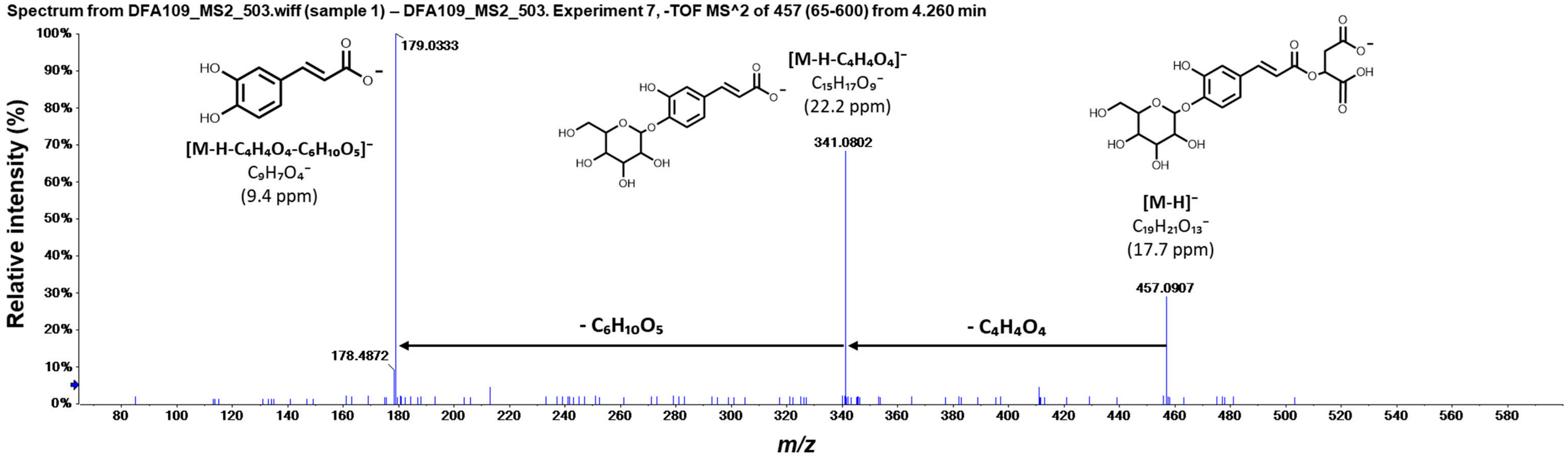

Figure S4-3. Tandem mass spectrum of m/z 457.1 at tR 4.3 min corresponding to caffeoyl malate hexoside (**2**), annotated in the aqueous fraction of the total aq. ethanolic extract of aerial part of *G. rivale* L. The spectrum was acquired with a hybrid QqTOF mass spectrometer operated in the negative product ion mode with unit Q1 resolution (collision energy 20 eV) [3]. Structures shown are suggestions.

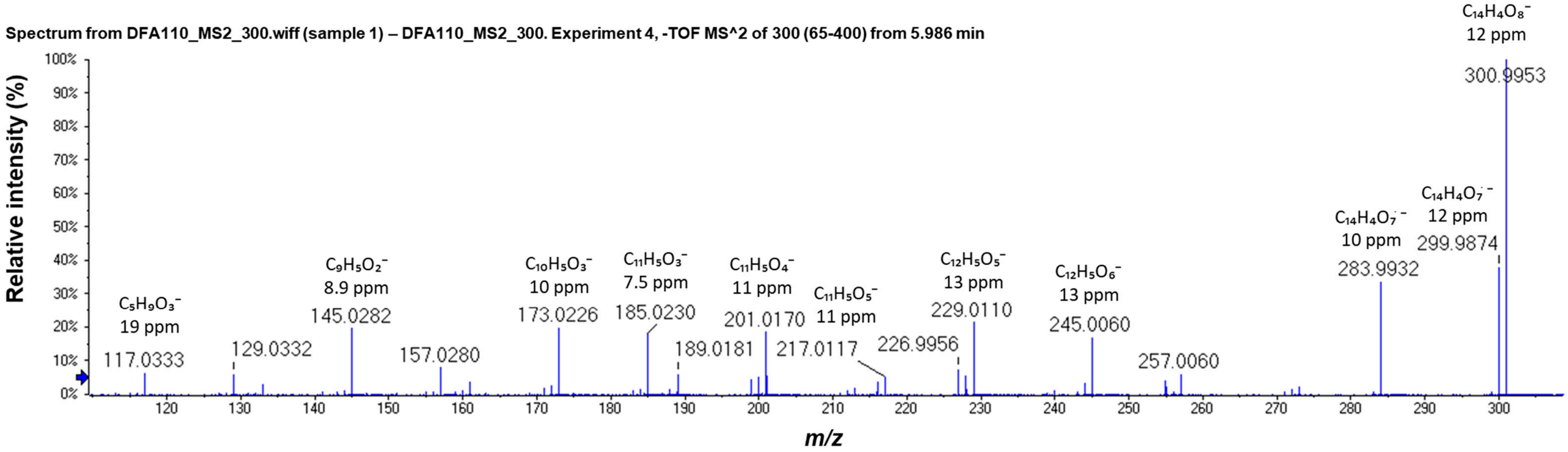

Figure S4-4 Tandem mass spectrum of *m/z* 300.99 at t_R 5.9 min corresponding to ellagic acid (**3**), annotated in the ethyl acetate fraction of total aq. ethanolic extracts of aerial part of *G. rivale* L. The spectrum was acquired with a hybrid QqTOF mass spectrometer operated in the negative product ion mode with unit Q1 resolution (collision energy 40 eV) [4].

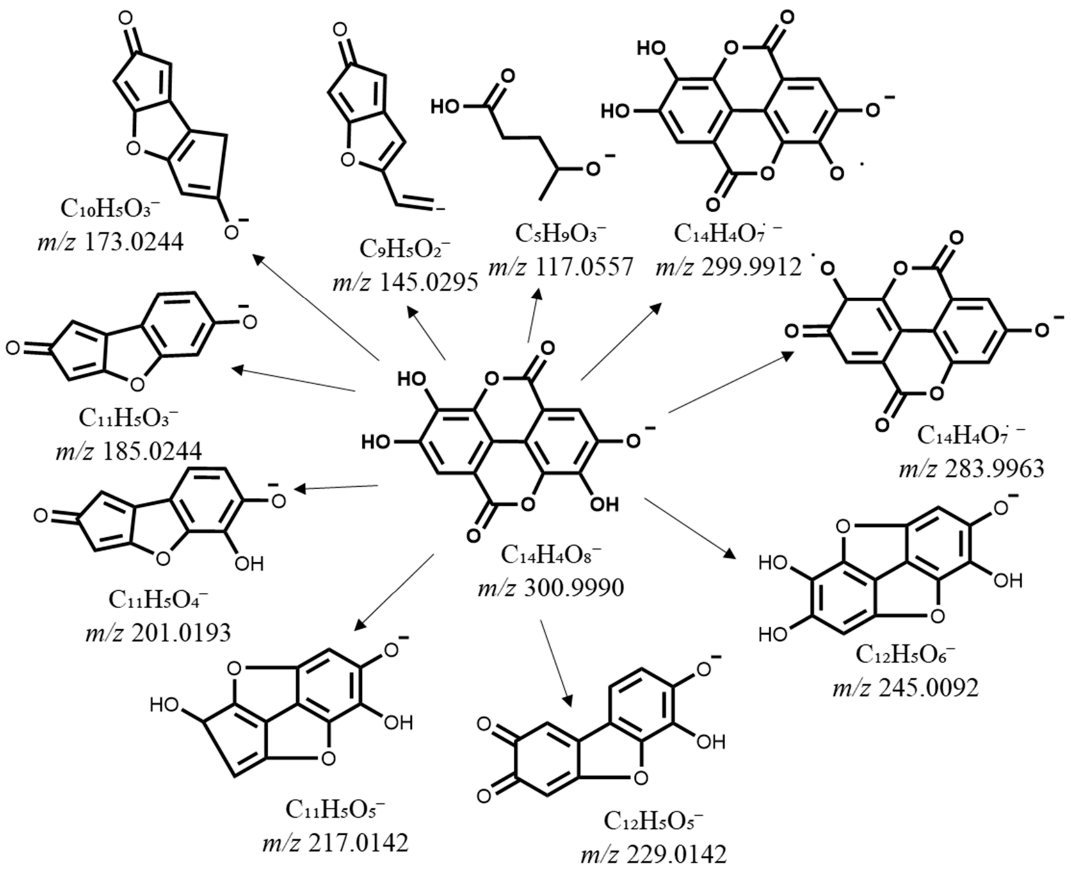

Figure S4-5. Proposed tandem mass spectrometric fragmentation patterns for the ion at *m/z* 300.99 ($[M\text{-}H]^-$, MS^2) corresponding to ellagic acid (**3**) [4].

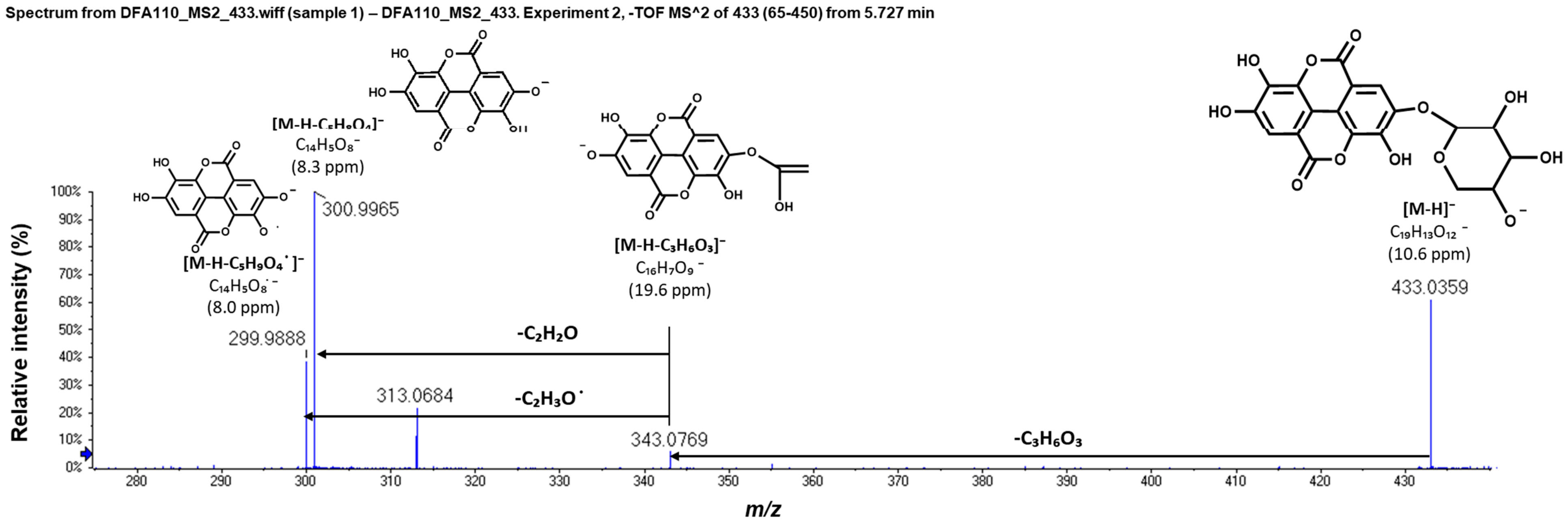

Figure S4-6. Tandem mass spectrum of *m/z* 433 at t_R 5.7 min corresponding to ellagic acid pentoside (**4**), annotated in ethyl acetate fraction of total aq. ethanolic extracts of aerial part of *G. rivale* L. The spectrum was acquired with a hybrid QqTOF mass spectrometer operated in the negative product ion mode with unit Q1 resolution (collision energy 20 eV) [3]. Structures shown are suggestions.

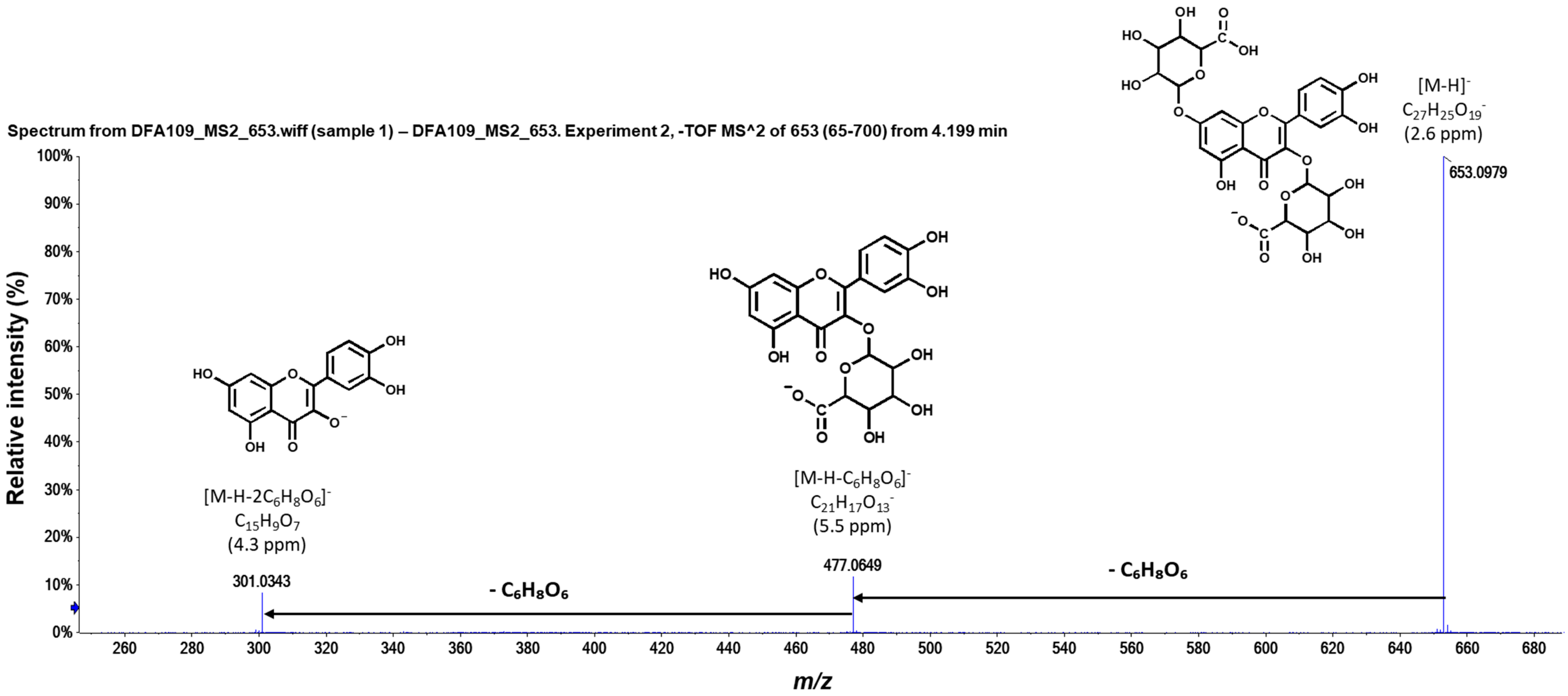

Figure S4-7. Tandem mass spectrum of *m/z* 653.1 at t_R 4.2 min corresponding to quercetin-bis-hexuronide (**5**), annotated in aqueous fraction of total aq. ethanolic extract of aerial part of *G. rivale* L. The spectrum was acquired with a hybrid QqTOF mass spectrometer operated in the negative product ion mode with unit Q1 resolution (collision energy 20 eV). Structures shown are suggestions.

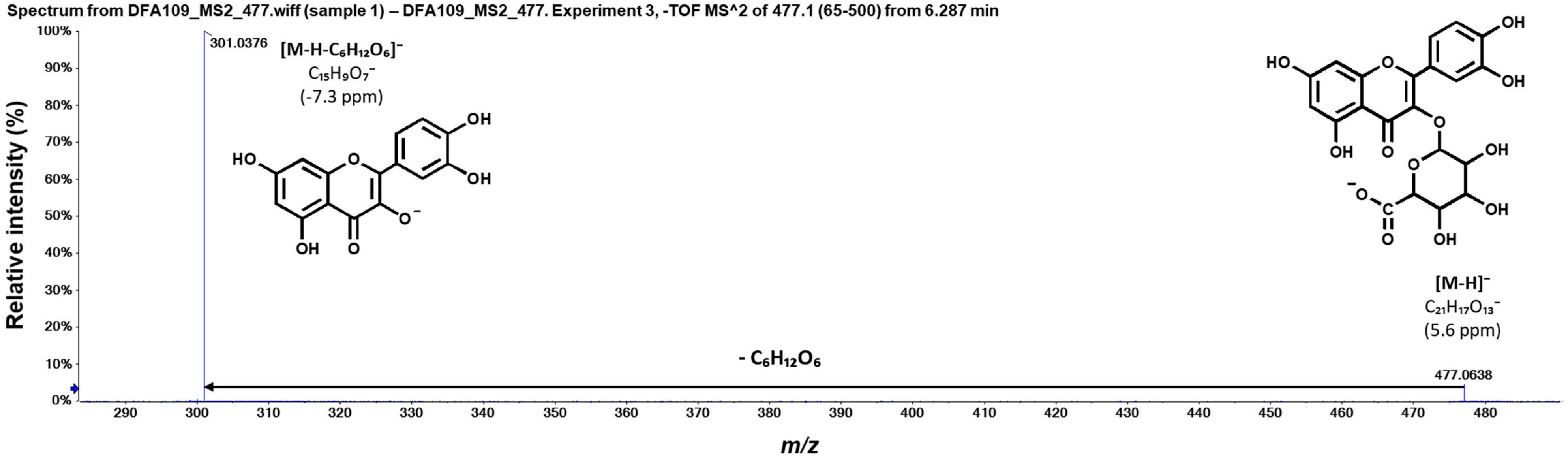

Figure S4-8. Tandem mass spectrum of *m/z* 477.1 at t_R 6.3 min corresponding to quercetin-hexuronide **(6)**, annotated in aqueous fraction of total aq. ethanolic extract of aerial part of *G. rivale* L. The spectrum was acquired with a hybrid QqTOF mass spectrometer operated in the negative product ion mode with unit Q1 resolution (collision energy 20 eV) [3]. Structures shown are suggestions.

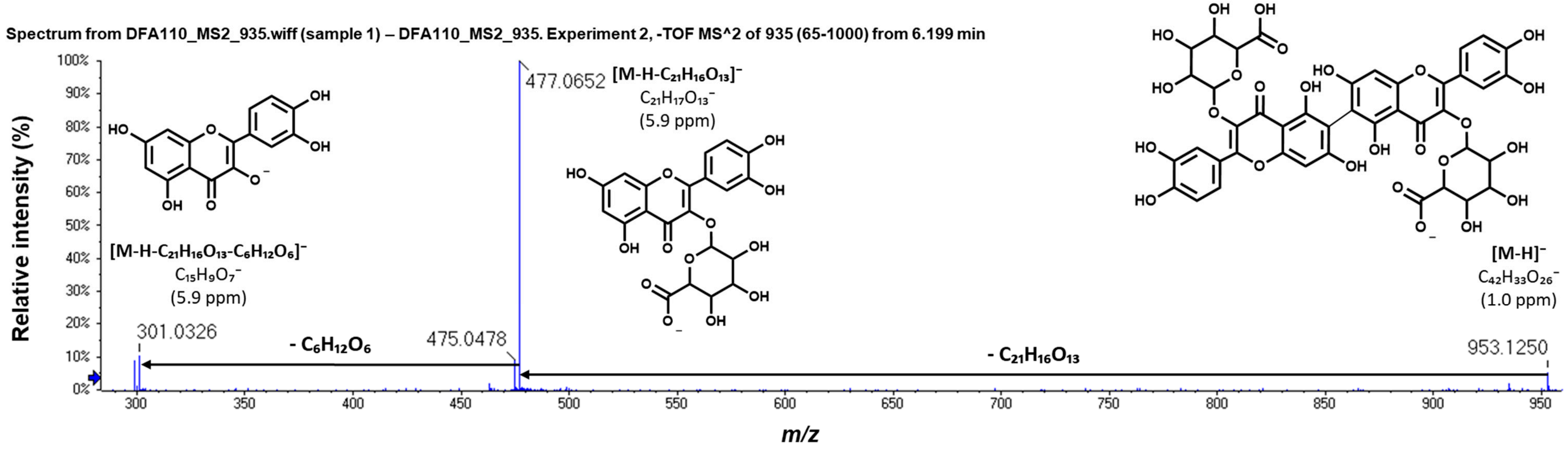

Figure S4-9. Tandem mass spectrum of *m/z* 953 at t_R 6.2 min corresponding to quercetin-hexuronide dimer (**7**), annotated in ethyl acetate fraction of total aq. ethanolic extracts of aerial part of *G. rivale* L. The spectrum was acquired with a hybrid QqTOF mass spectrometer operated in the negative product ion mode with unit Q1 resolution (collision energy 20 eV) [5]. Structures shown are suggestions.

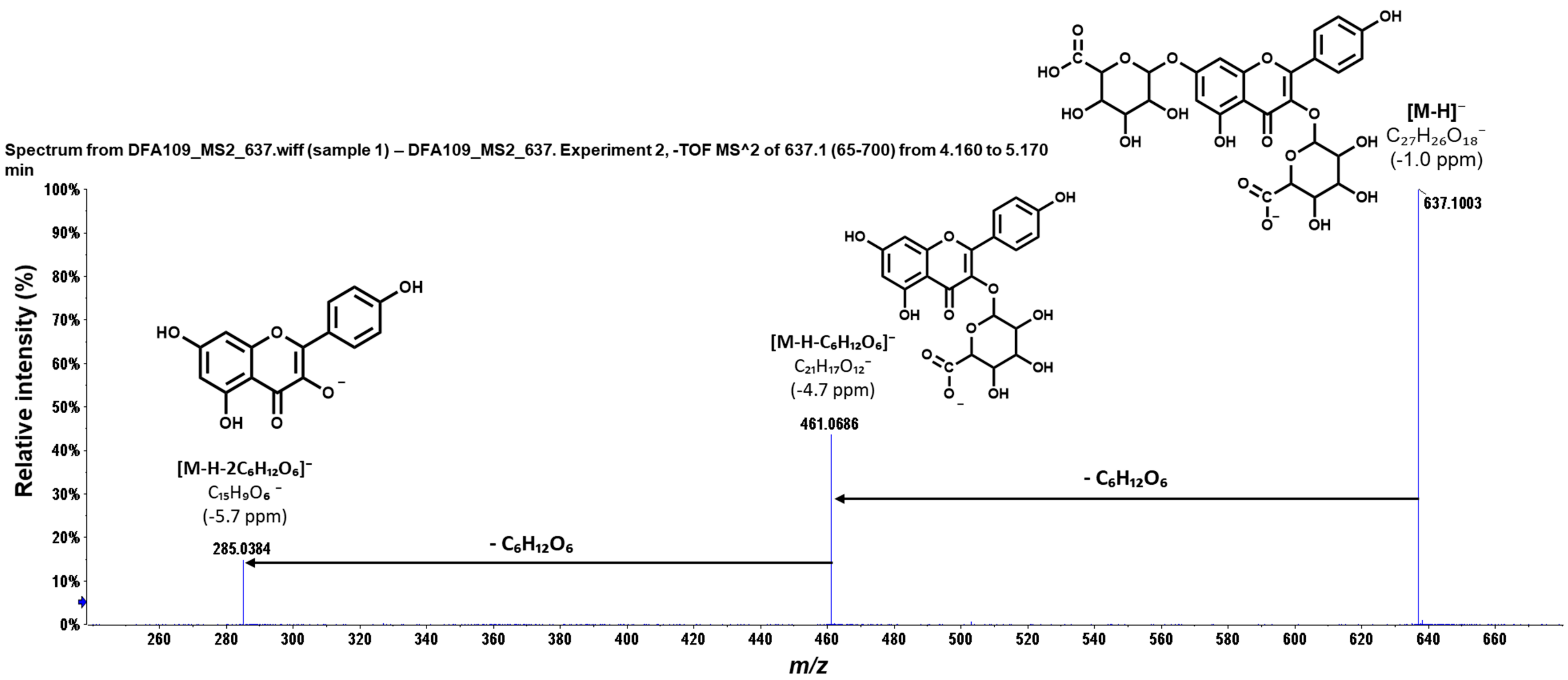

Figure S4-10. Tandem mass spectrum of *m/z* 637.1 at t_R 5.2 min corresponding to kaempferol-bis-*O*-hexuronide (**8**), annotated in aequeous fraction of total aq. ethanolic extract of aerial part of *G. rivale* L. The spectrum was acquired with a hybrid QqTOF mass spectrometer operated in the negative product ion mode with unit Q1 resolution (collision energy 20 eV). Structures shown are suggestions.

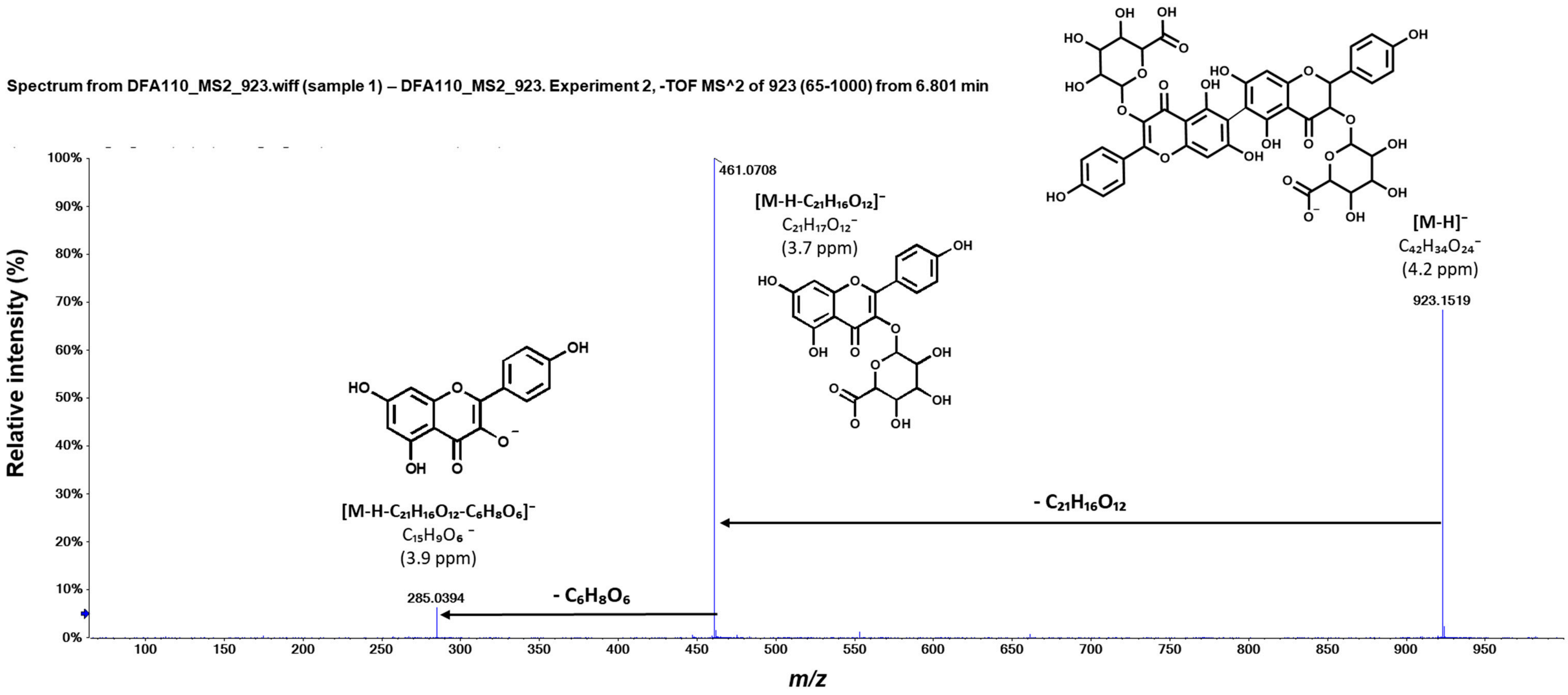

Figure S4-11. Tandem mass spectrum of *m/z* 923.2 at t_R 6.8 min corresponding to dihydrokaempferol-kaempferol-hexuronide dimer (**9**), annotated in ethyl acetate fraction of total aq. ethanolic extracts of aerial part of *G. rivale* L. The spectrum was acquired with a hybrid QqTOF mass spectrometer operated in the negative product ion mode with unit Q1 resolution (collision energy 20 eV) [5]. Structures shown are suggestions.

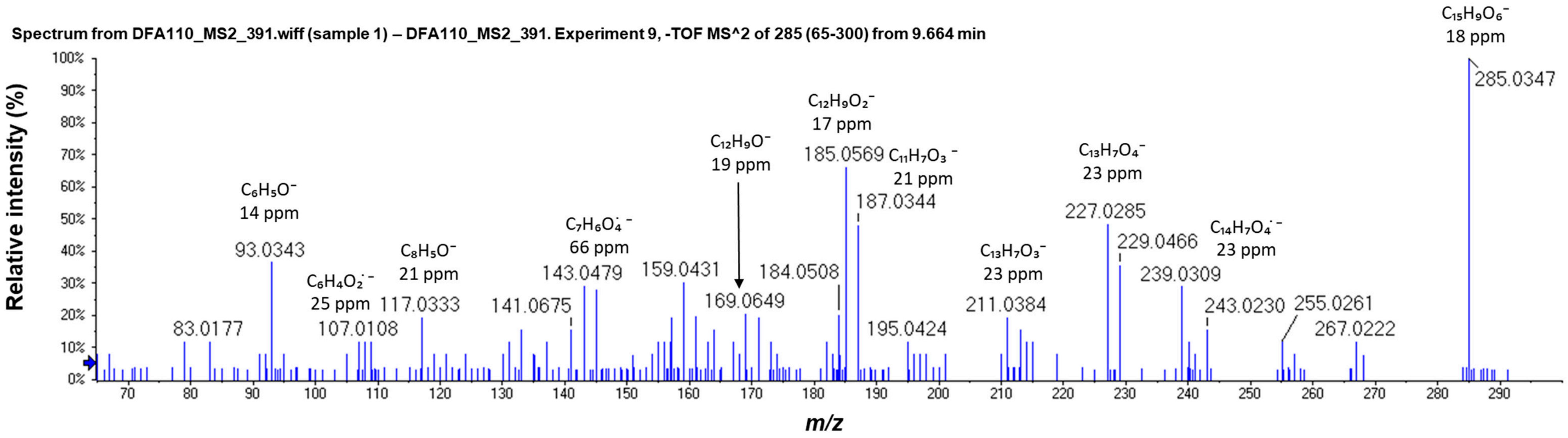

Figure S4-12. Tandem mass spectrum of *m/z* 285 at t_R 9.7 min corresponding to kaempferol (**10**), annotated in ethyl acetate fraction of total aq. ethanolicextracts of aerial part of *G. rivale* L. The spectrum was acquired with a hybrid QqTOF mass spectrometer operated in the negative product ion mode with unit Q1 resolution (collision energy 40 eV) [6].

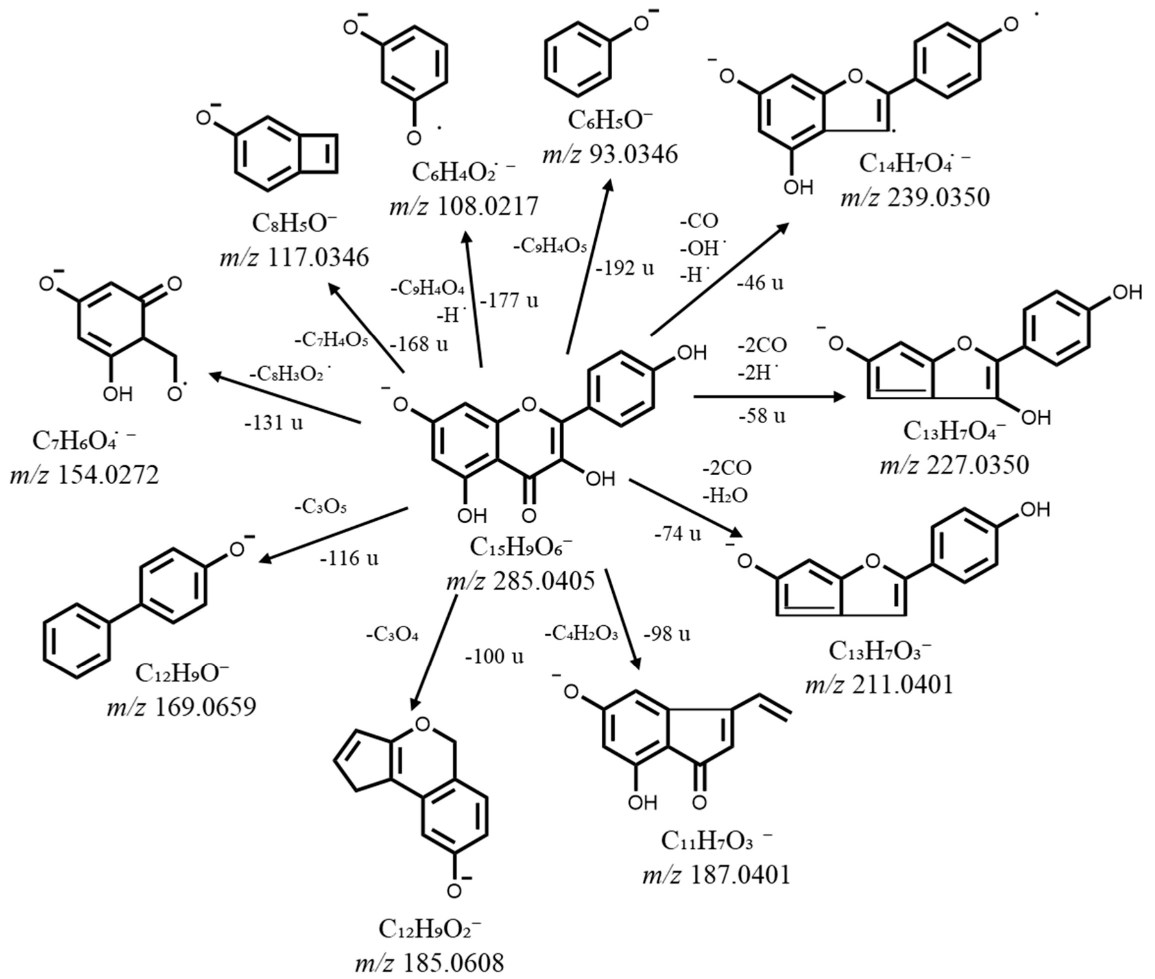

Figure S4-13. Proposed tandem mass spectrometric fragmentation patterns for the ion at *m/z* 285 ($[M\text{-}H]^-$, MS^2) corresponding to kaempferol (**10**) [6].

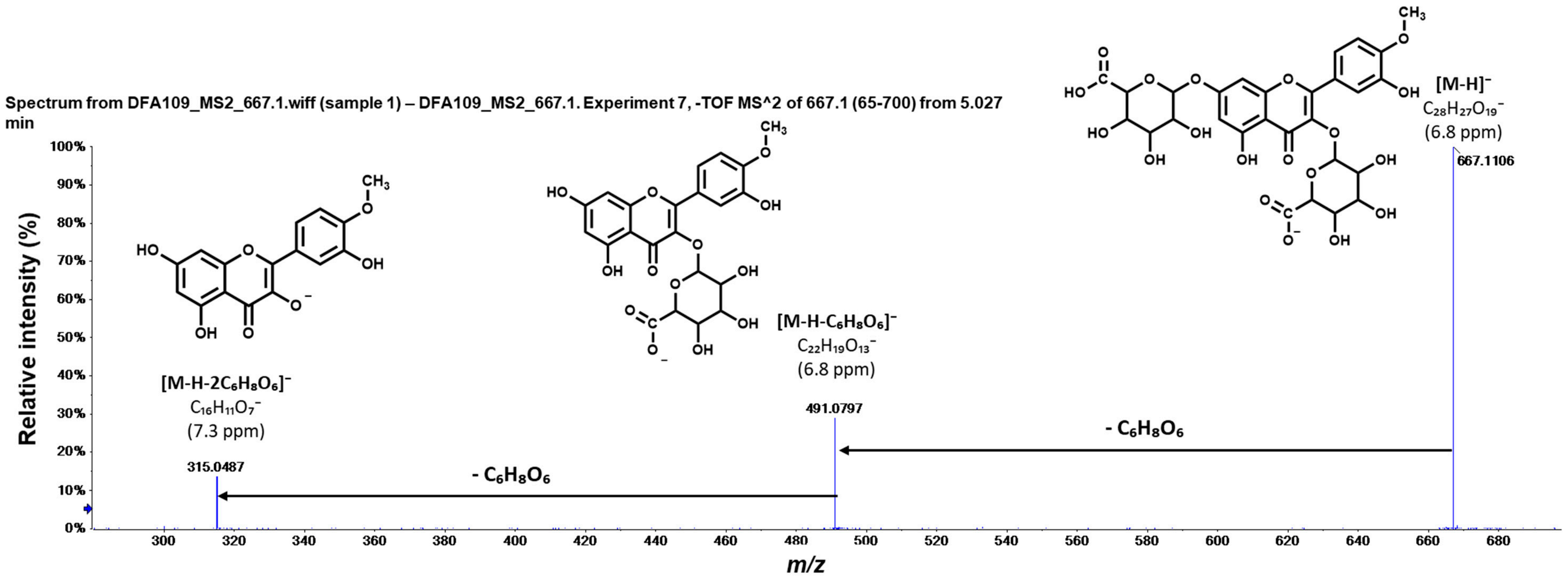

Figure S4-14. Tandem mass spectrum of *m/z* 667.1 at t_R 5.0 min corresponding to isorhamnetin-bis-glucuronide (**12**), annotated in aqueous fraction of total aq. ethanolic extract of aerial part of *G. rivale* L. The spectrum was acquired with a hybrid QqTOF mass spectrometer operated in the negative product ion mode with unit Q1 resolution (collision energy 20 eV). Structures shown are suggestions.

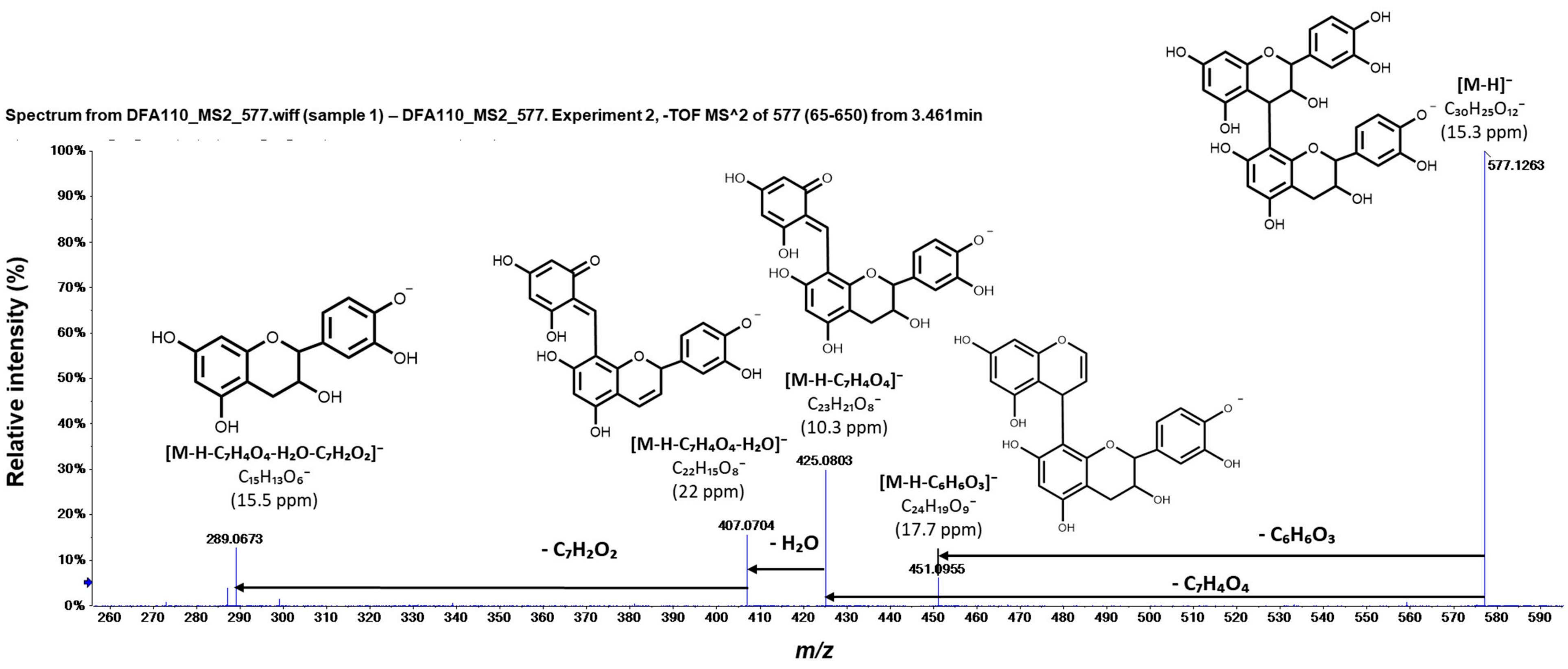

Figure S4-15. Tandem mass spectrum of *m/z* 577 at t_R 3.5 min corresponding to proanthocyanidin (**15**) annotated in ethyl acetate fraction of total aq. ethanolic extracts of aerial part of *G. rivale* L. The spectrum was acquired with a hybrid QqTOF mass spectrometer operated in the negative product ion mode with unit Q1 resolution (collision energy 20 eV) [3]. Structures shown are suggestions.

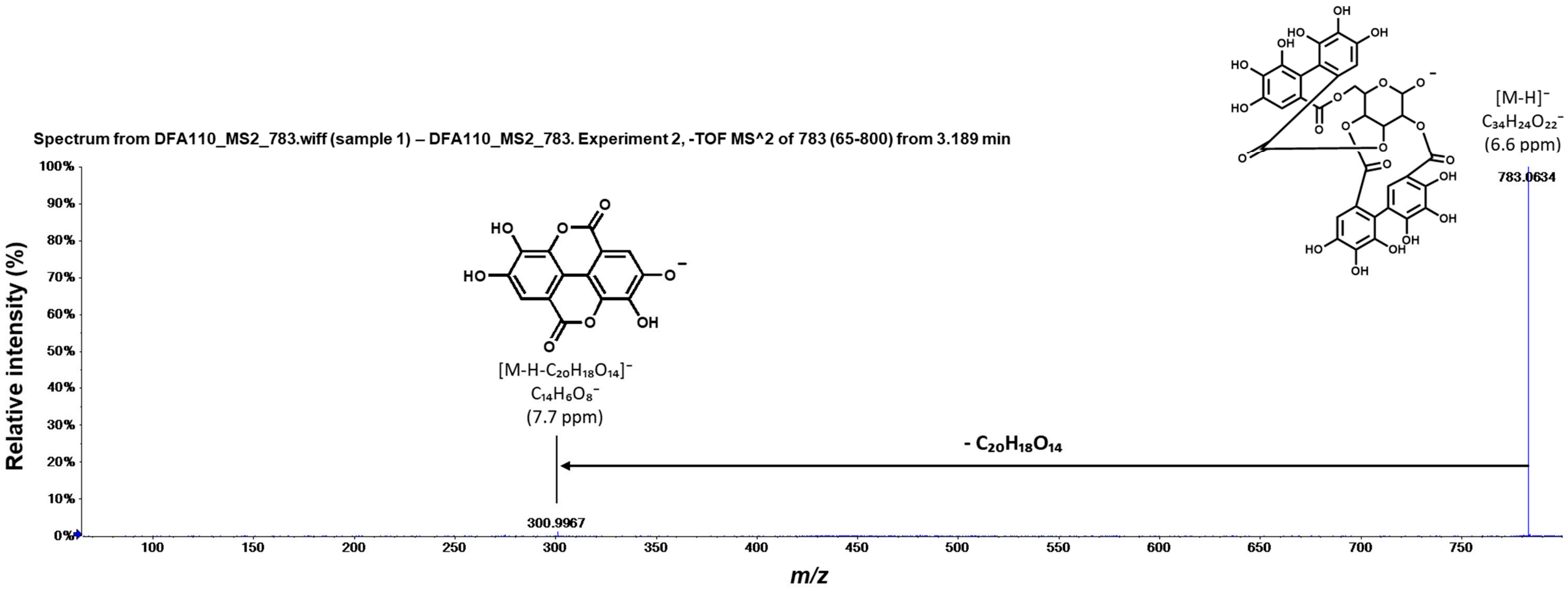

Figure S4-16. Tandem mass spectrum of *m/z* 783 at t_R 3.2 min corresponding to bis-HHDP-hexose (**16a**) in ethyl acetate fraction of aq. ethanolic extract of aerial part of *G. rivale* L. The spectrum was acquired with a hybrid QqTOF mass spectrometer operated in the negative product ion mode with unit Q1 resolution (collision energy 20 eV) [3]. Structures shown are suggestions.

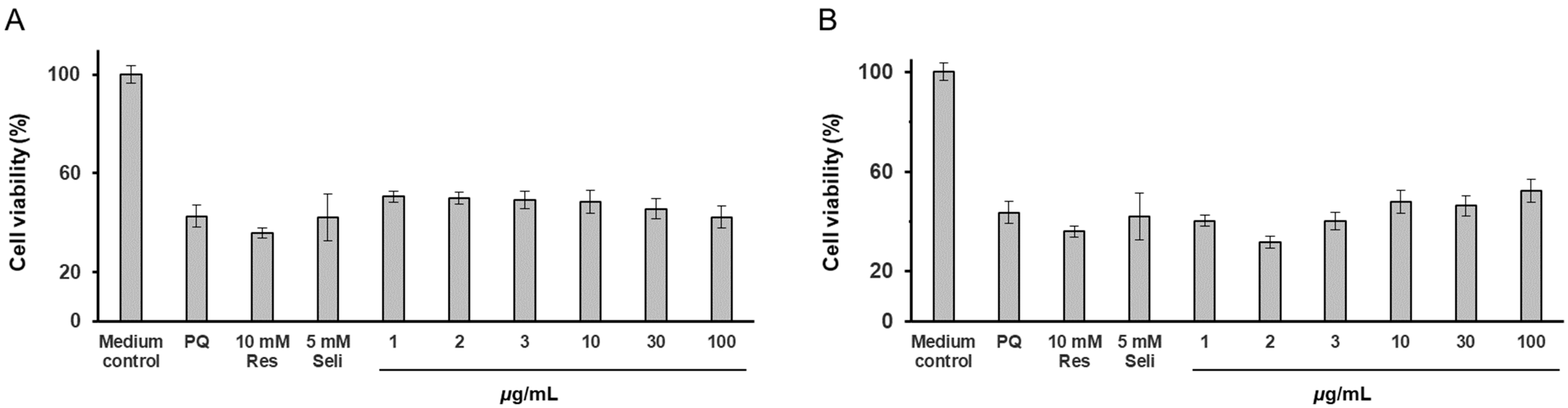

Figure S5 Results of the MTT assay addressing toxicity of the fractions of total extracts of *Geum rivale* L.: aqueous fraction of total extract (A); ethyl acetate fraction of total extract (B). n = 4 for experimental groups, n = 8 for control groups. PQ – paraquat; Res – resveratrol; Seli – seligelin.

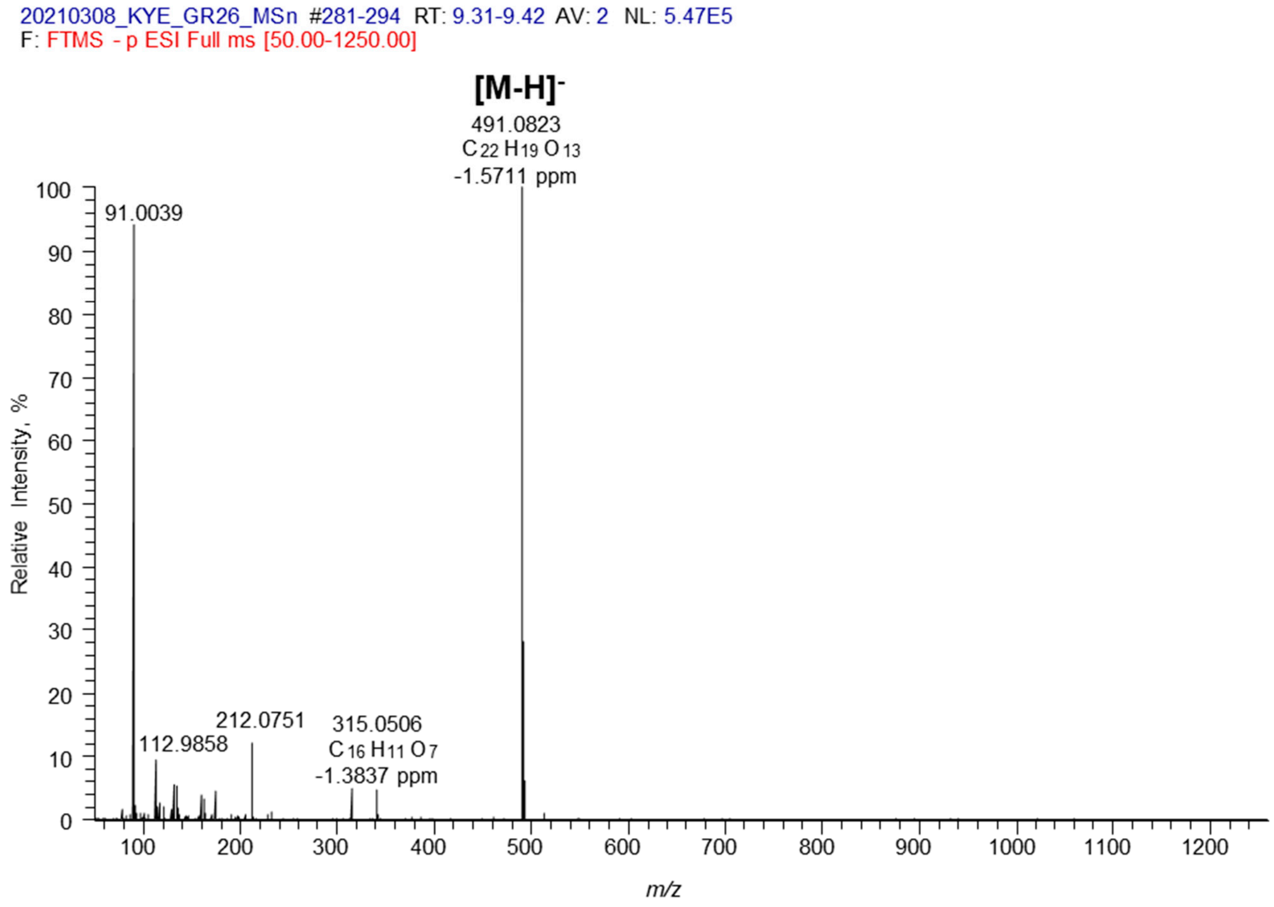

Figure S6-1 High-resolution electrospray ionization mass spectrum (HR-ESI-MS) with a signal of the $[M-H]^-$ ion at *m/z* 491 corresponding to isorhamnetin-3-*O*-β-D-glucuronide. The mass spectrum was acquired with a hybrid linear ion trap-orbital trap mass spectrometer (LIT-Orbitrap-MS) operated in negative ion mode.

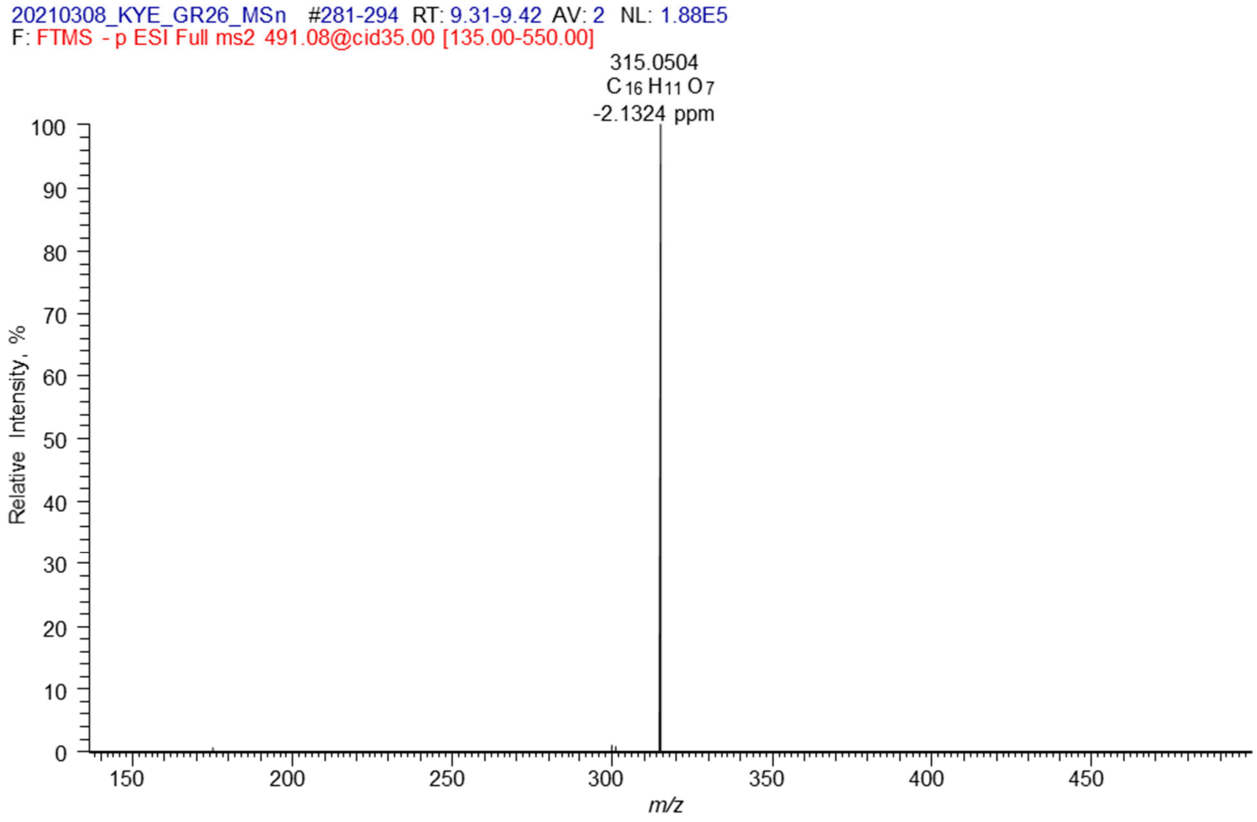

Figure S6-2 High-resolution electrospray ionization tandem mass spectrum (HR-ESI-MS^2) of the [M-H]$^-$ ion at *m/z* 491 corresponding to isorhamnetin-3-*O*-β-D-glucuronide. The mass spectrum was acquired with a hybrid linear ion trap-orbital trap mass spectrometer (LIT-Orbitrap-MS) operated in negative ion mode with collision induced dissociation (both steps - relative collision energy CE 35%, trap resonance collisional activation CID 35%).

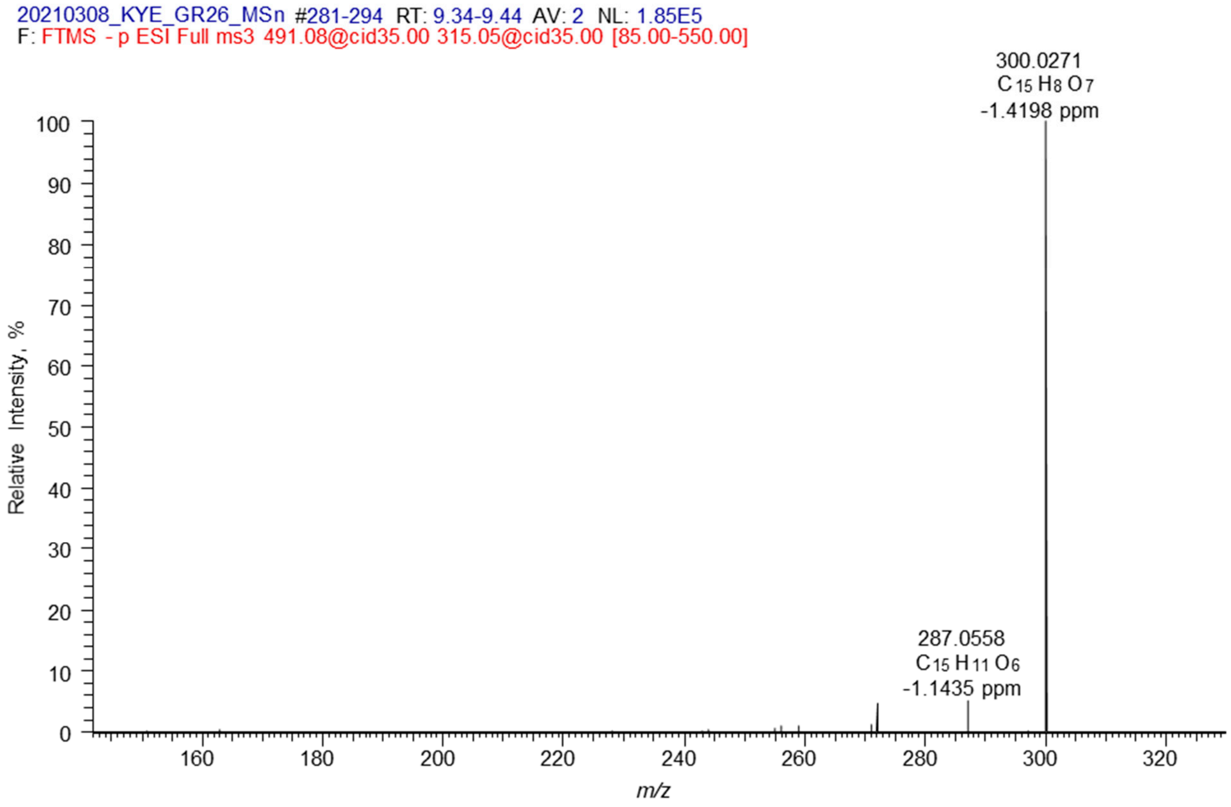

Figure S6-3. High-resolution electrospray ionization tandem mass spectrum (HR-ESI-MS^3 [491 → 315]) of the $[M-H]^-$ ion at *m/z* 491 corresponding to isorhamnetin-3-*O*-β-D-glucuronide. The mass spectrum was acquired with a hybrid linear ion trap-orbital trap mass spectrometer (LIT-Orbitrap-MS) operated in negative ion mode with collision induced dissociation (relative collision energy CE 35%, trap resonance collisional activation CID 35%).

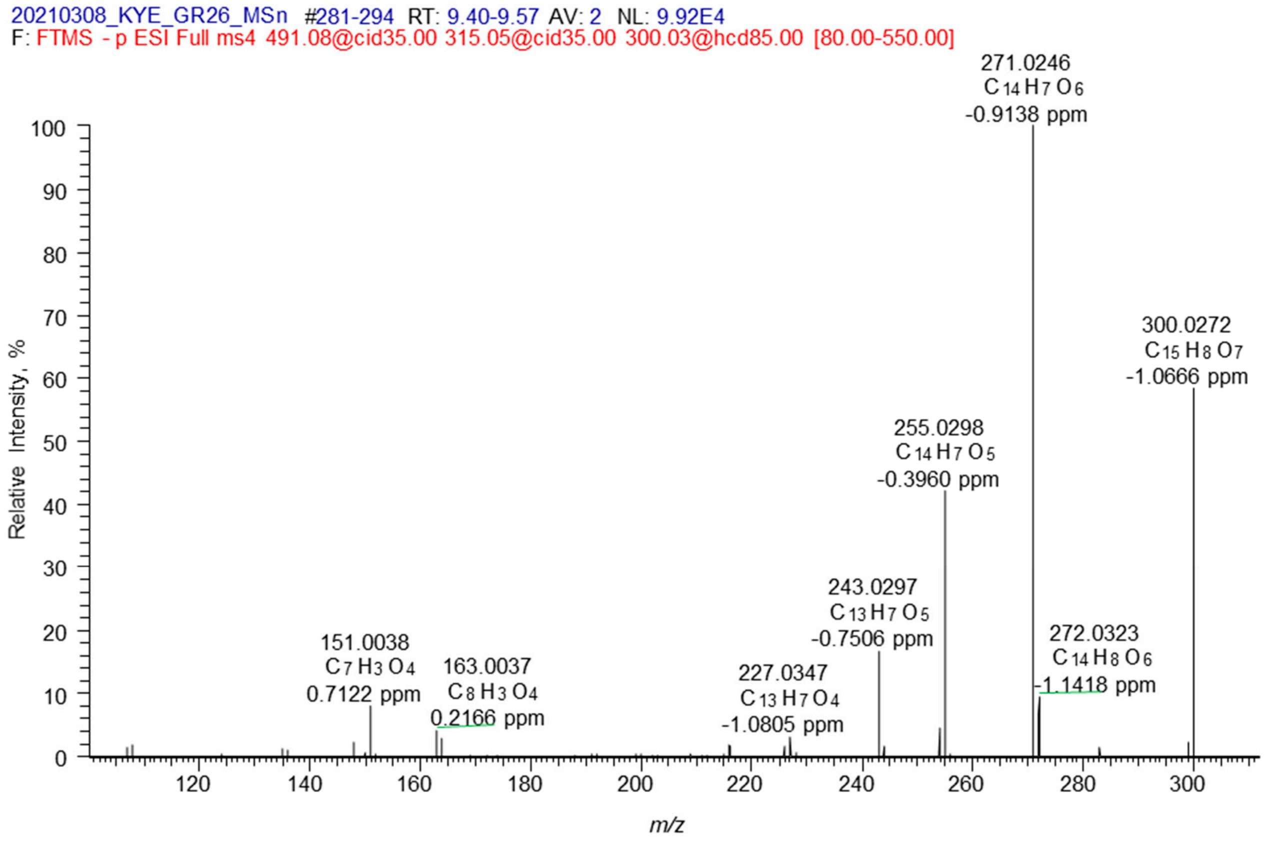

Figure S6-4. High-resolution electrospray ionization tandem mass spectrum (HR-ESI-MS^4 [491→ 315 → 300]) of the $[M-H]^-$ ion at *m/z* 491 corresponding to isorhamnetin-3-*O*-β-D-glucuronide. The mass spectrum was acquired with a hybrid linear ion trap-orbital trap mass spectrometer (LIT-Orbitrap-MS) operated in negative ion mode with higher-energy C-trap dissociation HCD 85%.

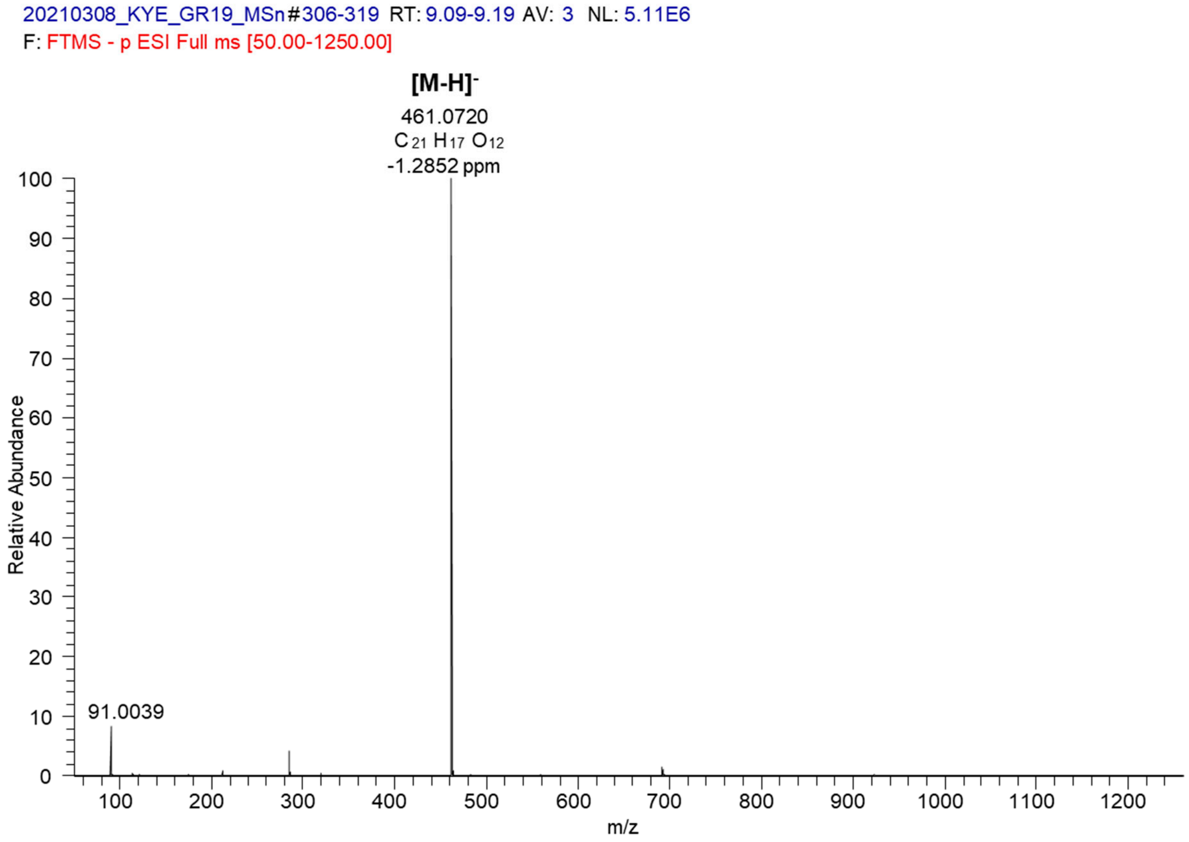

Figure S7-1. High-resolution electrospray ionization mass spectrum (HR-ESI-MS) with a signal of the $[M-H]^-$ ion at *m/z* 461 corresponding to kaempferol-3-*O*-β-D-glucuronide. The mass spectrum was acquired with a hybrid linear ion trap-orbital trap mass spectrometer (LIT-Orbitrap-MS) operated in negative ion mode.

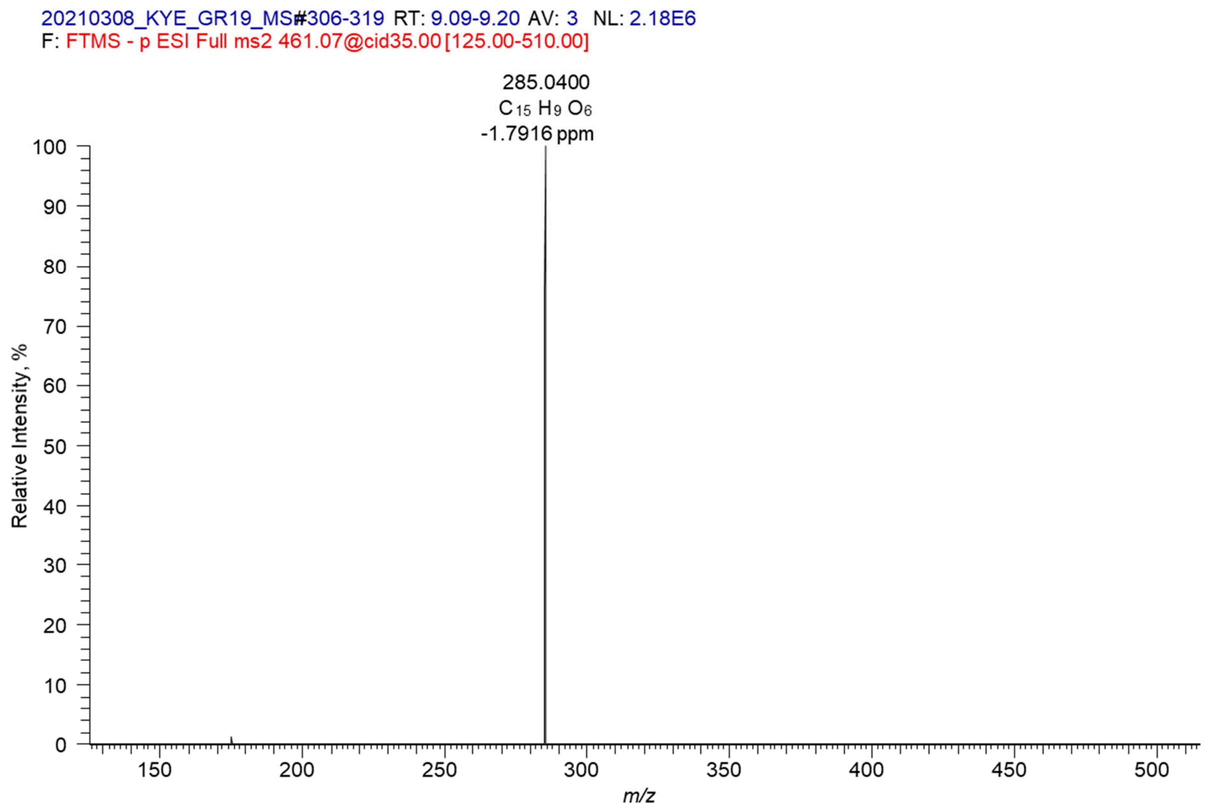

Figure S7-2. High-resolution electrospray ionization tandem mass spectrum (HR-ESI-MS^2) of the $[M\text{-}H]^-$ ion at *m/z* 461 corresponding to kaempferol-3-*O*-β-D-glucuronide. The mass spectrum was acquired with a hybrid linear ion trap-orbital trap mass spectrometer (LIT-Orbitrap-MS) operated in negative ion mode with collision induced dissociation (relative collision energy CE 35%, trap resonance collisional activation CID 35%).

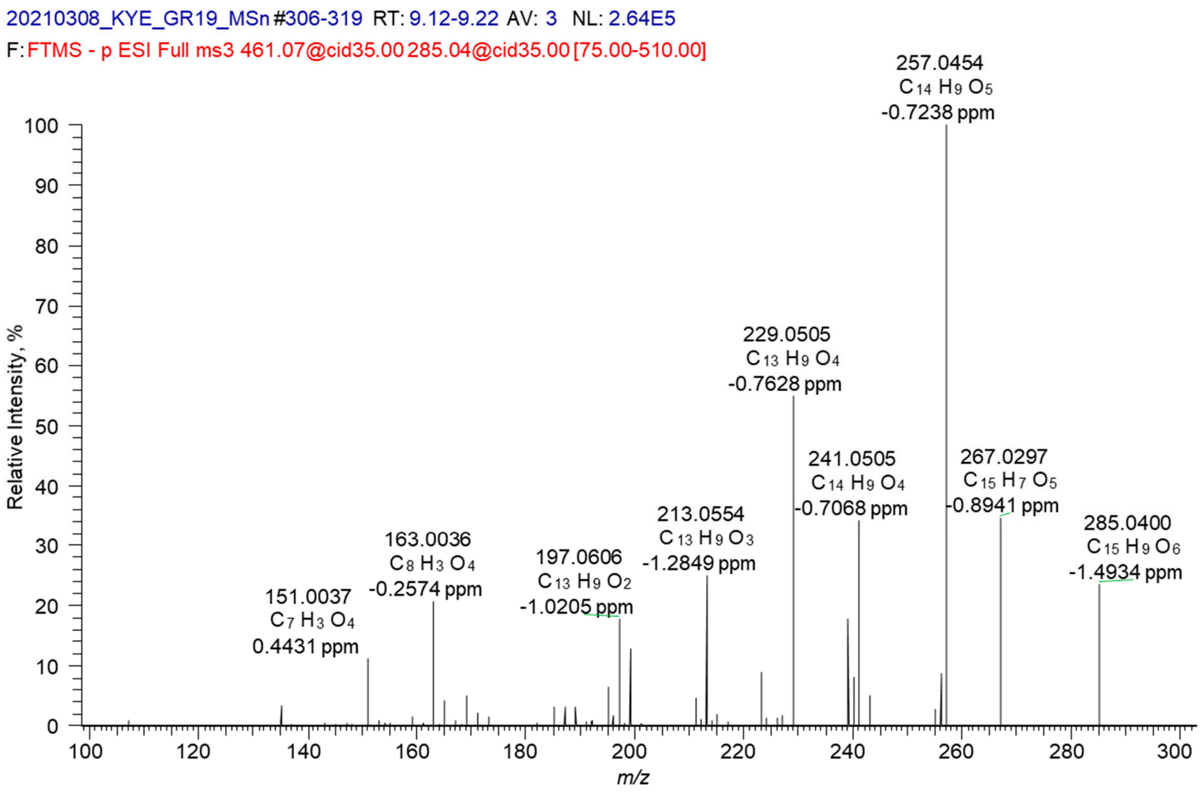

Figure S7-3. High-resolution electrospray ionization tandem mass spectrum (HR-ESI-MS^3) of the kaempferol-3-*O*-β-D-glucuronide fragment ion found at *m/z* 285 [461 → 285]. The mass spectrum was acquired with a hybrid linear ion trap-orbital trap mass spectrometer (LIT-Orbitrap-MS) operated in negative ion mode with collision induced dissociation (relative collision energy CE 35%, trap resonance collisional activation CID 35%).

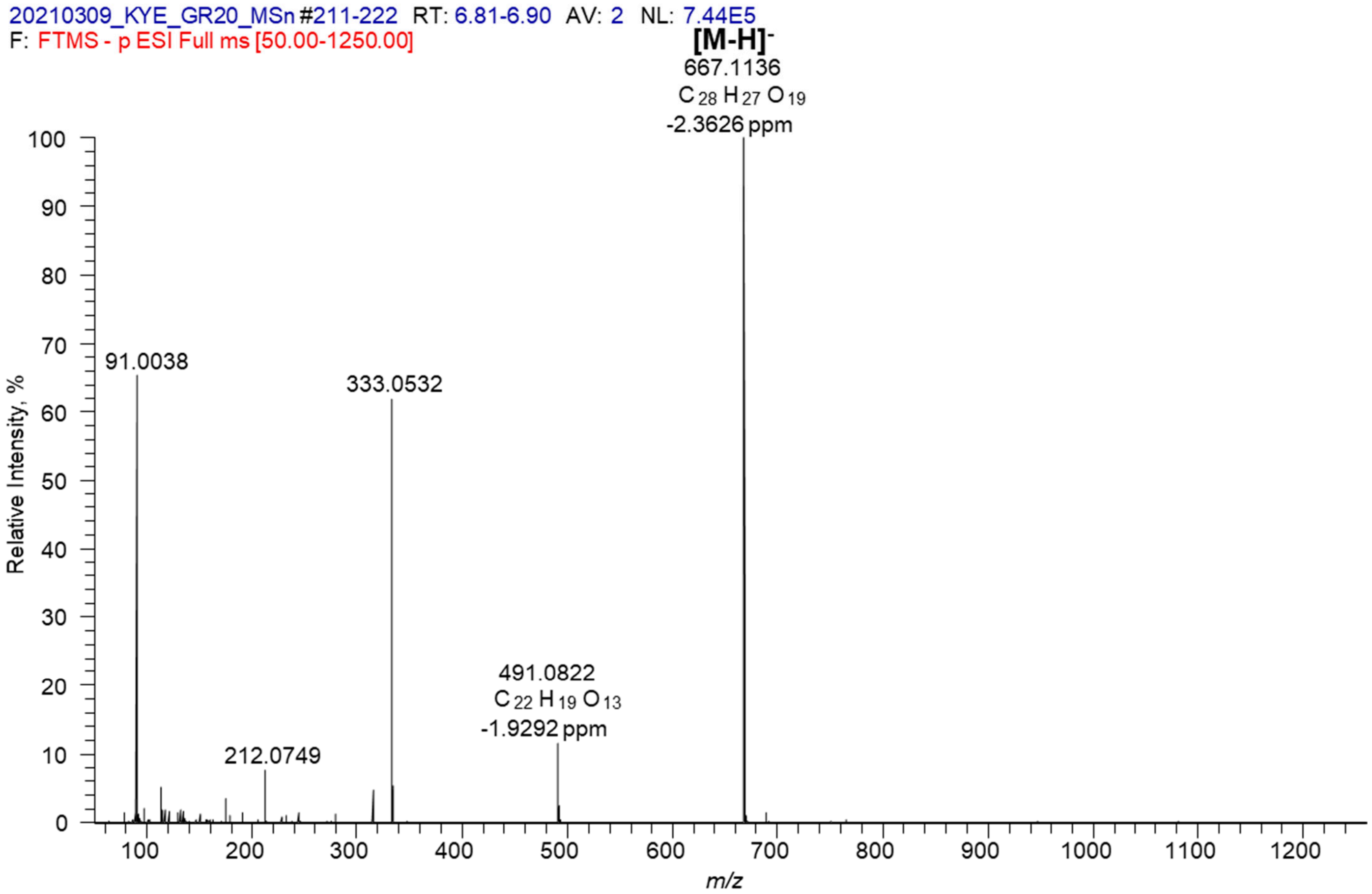

Figure S8-1. High-resolution electrospray ionization mass spectrum (HR-ESI-MS) with a signal of the [M-H]$^-$ ion at *m/z* 667 corresponding to isorhamnetin-bis-3,7-*O*-β-D-glucuronide. The mass spectrum was acquired with a hybrid linear ion trap-orbital trap mass spectrometer (LIT-Orbitrap-MS) operated in negative ion mode.

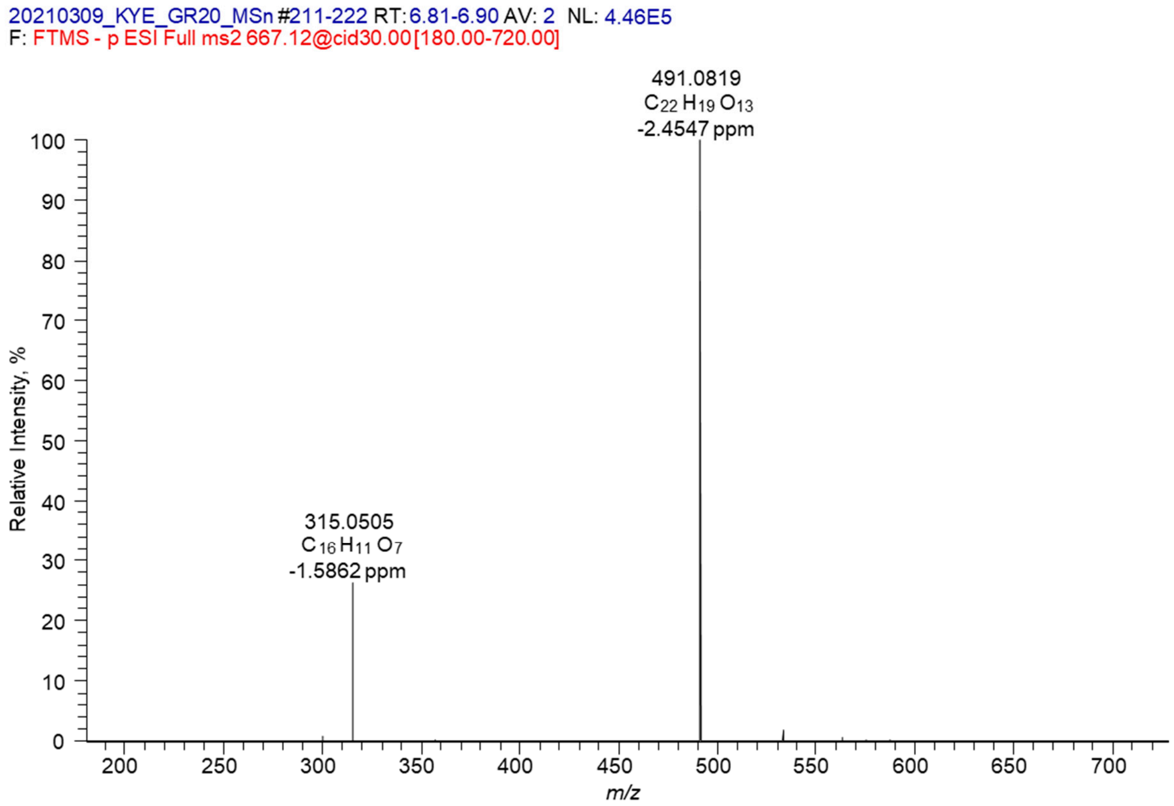

Figure S8-2. High-resolution electrospray ionization tandem mass spectrum (HR-ESI-MS^2) of the $[M\text{-}H]^-$ ion at *m/z* 667 corresponding to isorhamnetin-bis-3,7-*O*-β-D-glucuronide. The mass spectrum was acquired with a hybrid linear ion trap-orbital trap mass spectrometer (LIT-Orbitrap-MS) operated in negative ion mode with collision induced dissociation (relative collision energy CE 30%, trap resonance collisional activation CID 30%).

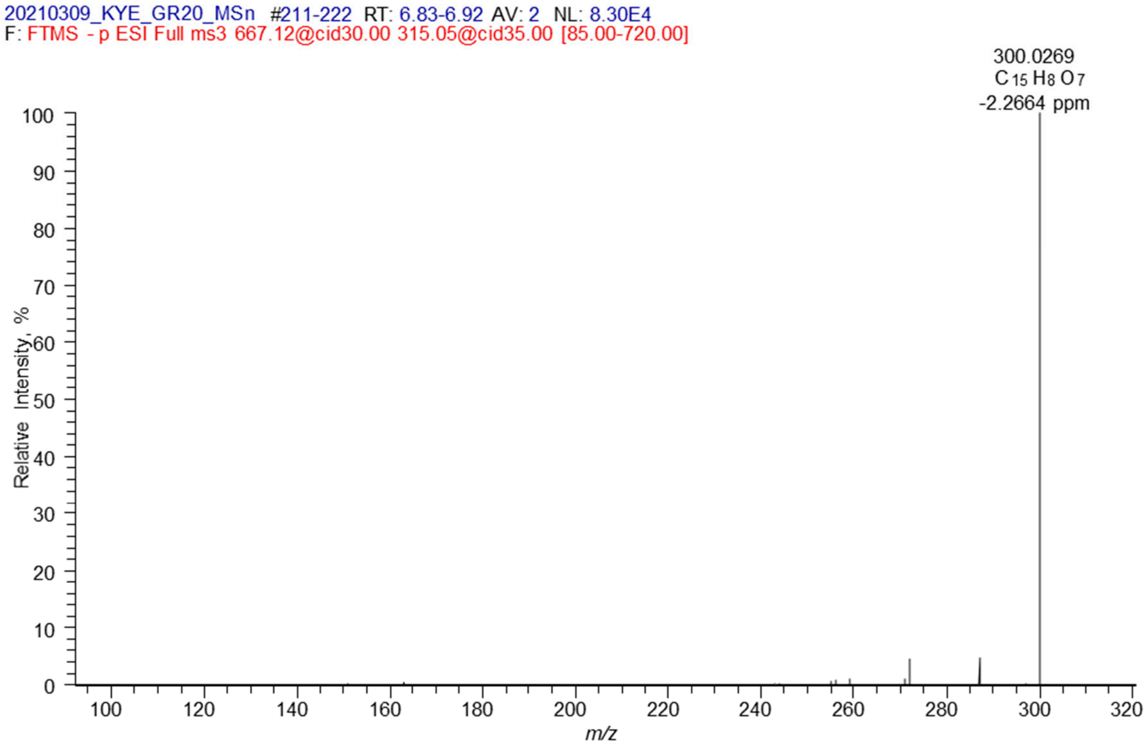

Figure S8-3. High-resolution electrospray ionization tandem mass spectrum (HR-ESI-MS^3 [667 → 315]) of the $[M-H]^-$ ion at *m/z* 667 corresponding to isorhamnetin-bis-3,7-*O*-β-D-glucuronide. The mass spectrum was acquired with a hybrid linear ion trap-orbital trap mass spectrometer (LIT-Orbitrap-MS) operated in negative ion mode with collision induced dissociation (relative collision energy CE 35%, trap resonance collisional activation CID 35%).

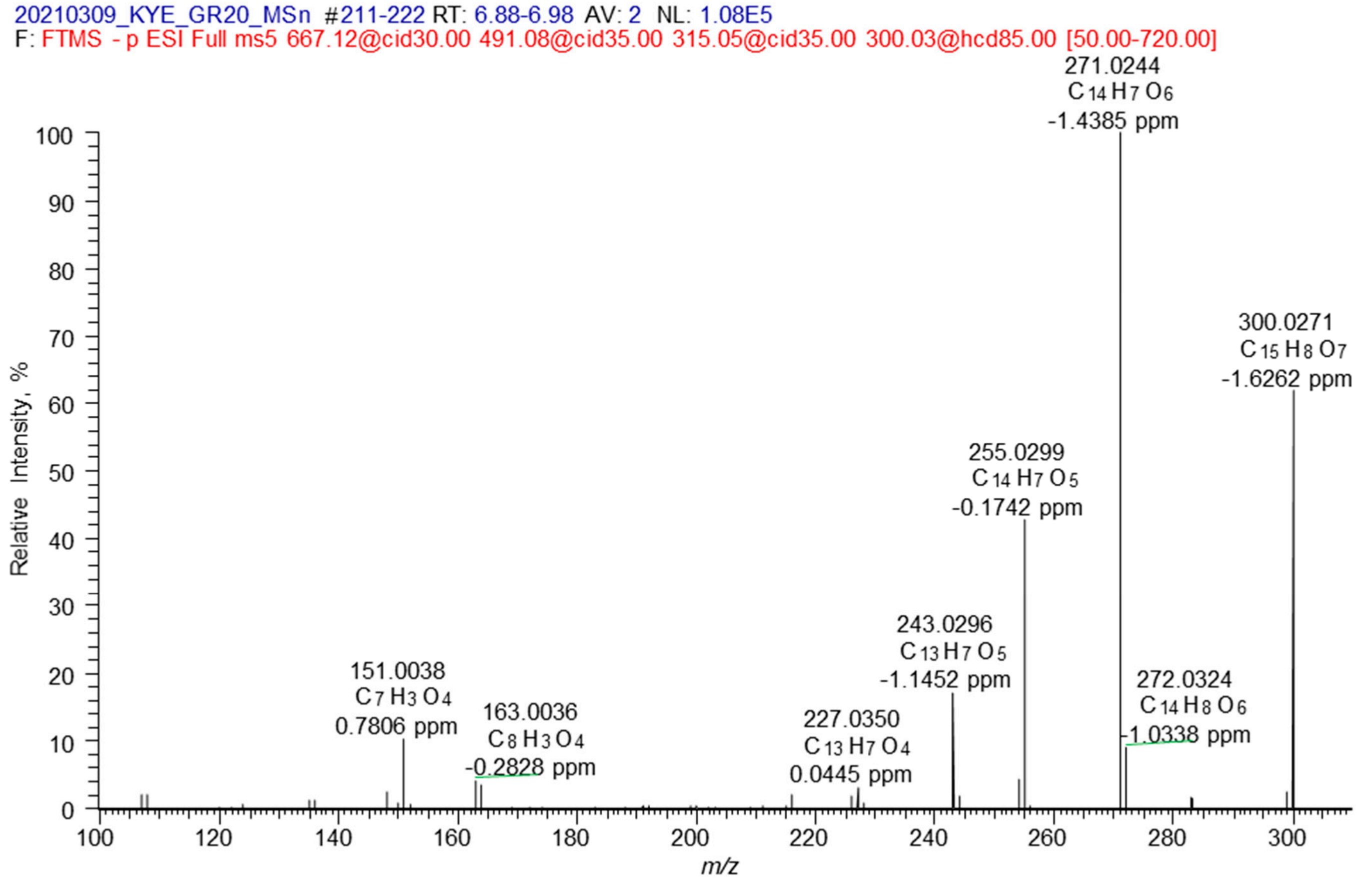

Figure S8-4. High-resolution electrospray ionization tandem mass spectrum (HR-ESI-MS^5 [667 → 491→ 315 →300]) of the [M-H]$^-$ ion at *m/z* 667 corresponding to isorhamnetin-bis-3,7-*O*-β-D-glucuronide. The mass spectrum was acquired with a hybrid linear ion trap-orbital trap mass spectrometer (LIT-Orbitrap-MS) operated in negative ion mode with higher-energy C-trap dissociation, HCD 85%.

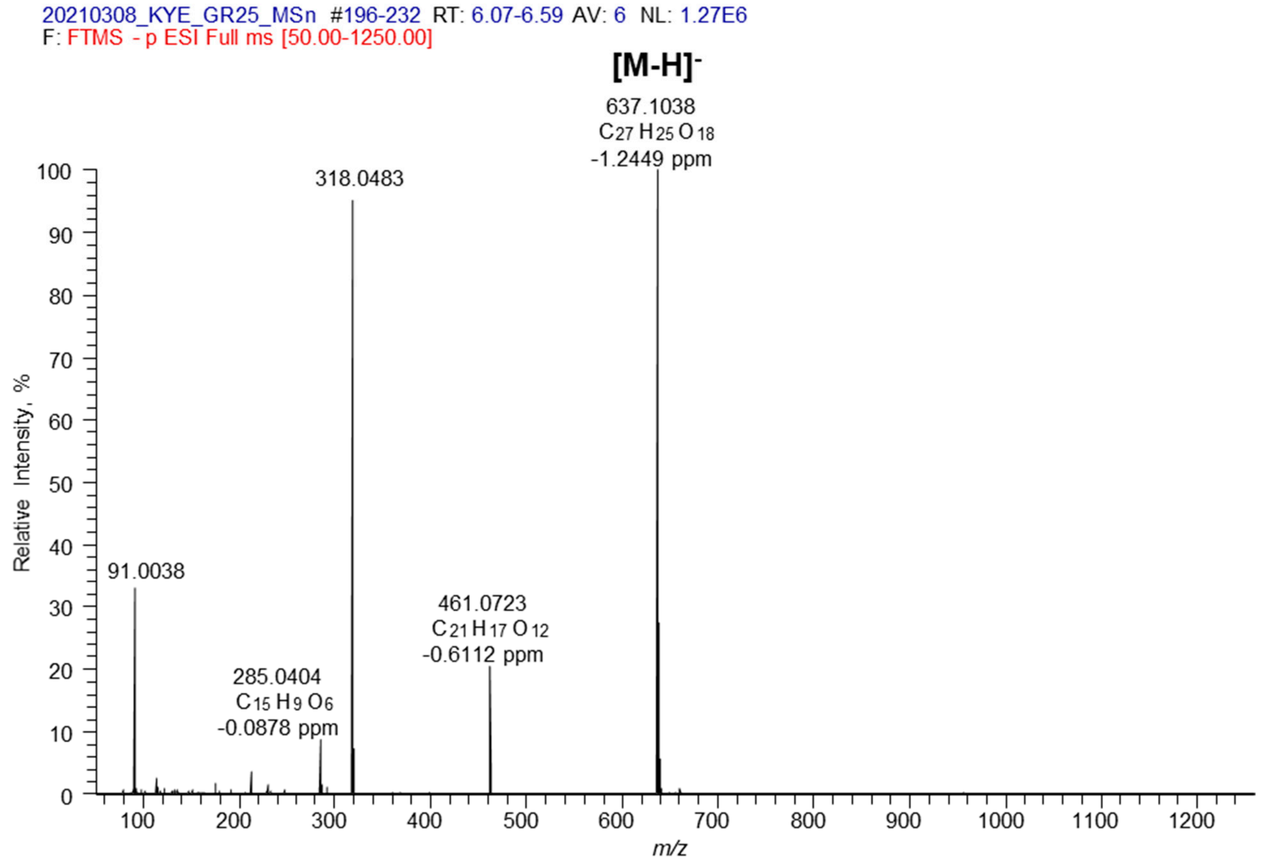

Figure S9-1. High-resolution electrospray ionization mass spectrum (HR-ESI-MS) with a signal of the $[M-H]^-$ ion at *m/z* 637 corresponding to kaempferol-bis-3,7-*O*-β-D-glucuronide. The mass spectrum was acquired with a hybrid linear ion trap-orbital trap mass spectrometer (LIT-Orbitrap-MS) operated in negative ion mode.

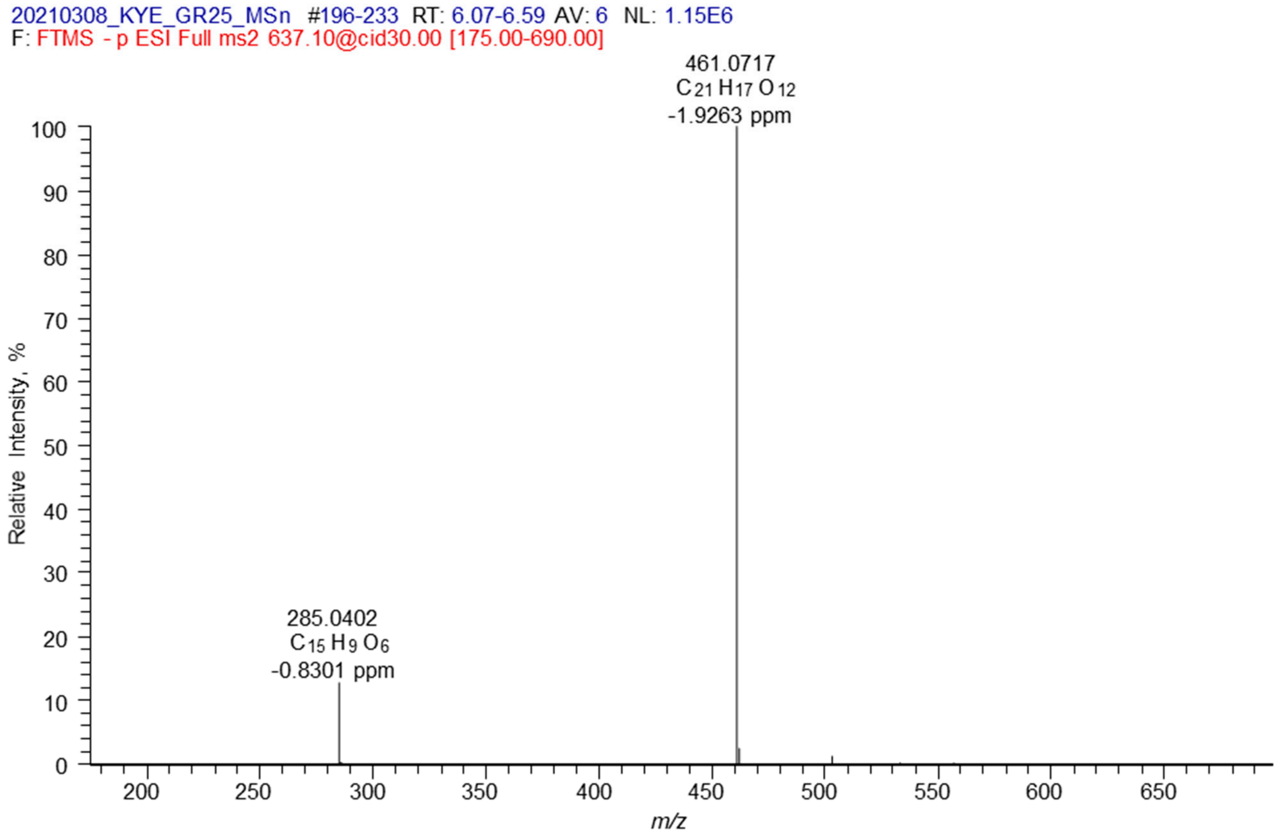

Figure S9-2. High-resolution electrospray ionization tandem mass spectrum (HR-ESI-MS^2) of the $[M-H]^-$ ion at *m/z* 637 corresponding to kaempferol-bis-3,7*O*-β-D-glucuronide. The mass spectrum was acquired with a hybrid linear ion trap-orbital trap mass spectrometer (LIT-Orbitrap-MS) operated in negative ion mode with collision induced dissociation (relative collision energy CE 30%, trap resonance collisional activation CID 30%).

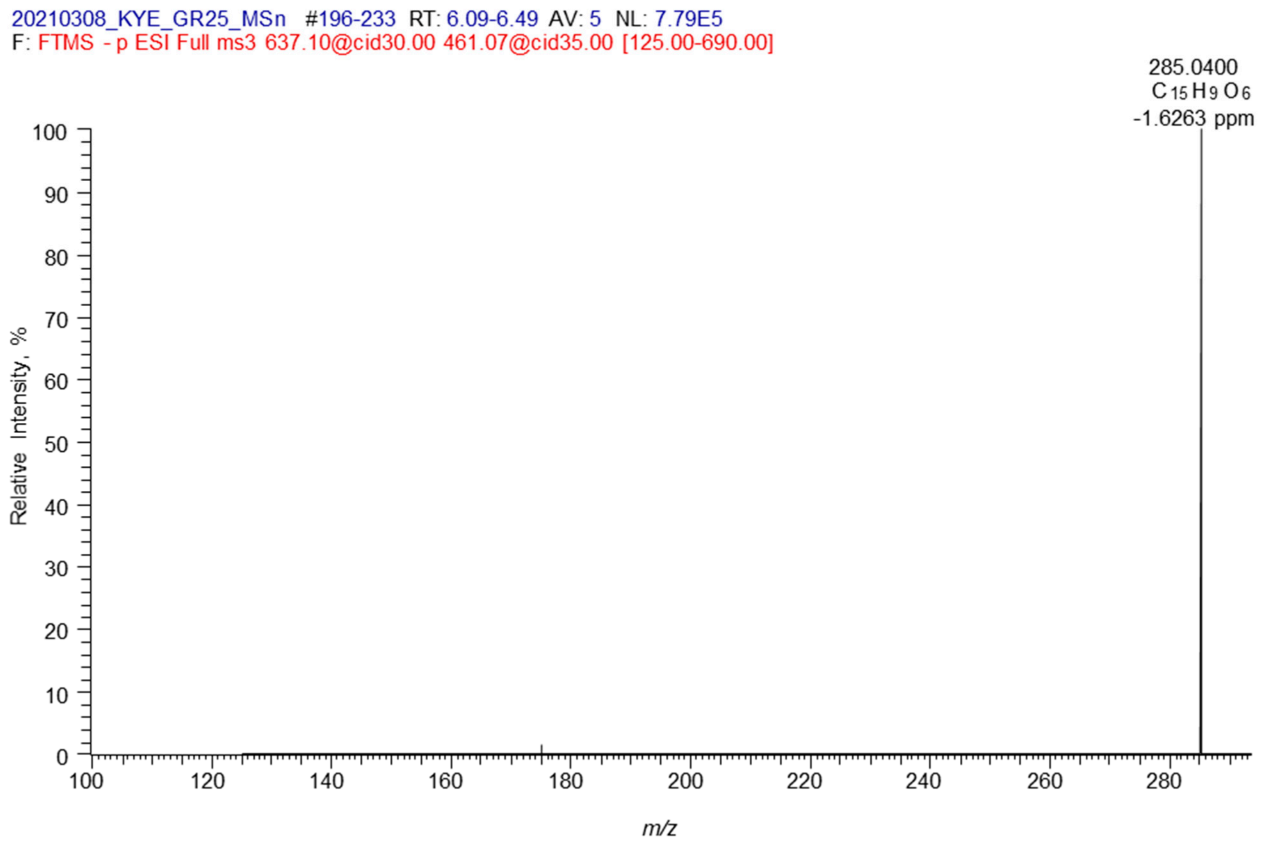

Figure S9-3. High-resolution electrospray ionization tandem mass spectrum (HR-ESI-MS^3 [637→ 461]) of the $[M-H]^-$ ion at *m/z* 637 corresponding to kaempferol-bis-3,7-*O*-β-D-glucuronide. The mass spectrum was acquired with a hybrid linear ion trap-orbital trap mass spectrometer (LIT-Orbitrap-MS) operated in negative ion mode with collision induced dissociation (relative collision energy CE 35%, trap resonance collisional activation CID 35%).

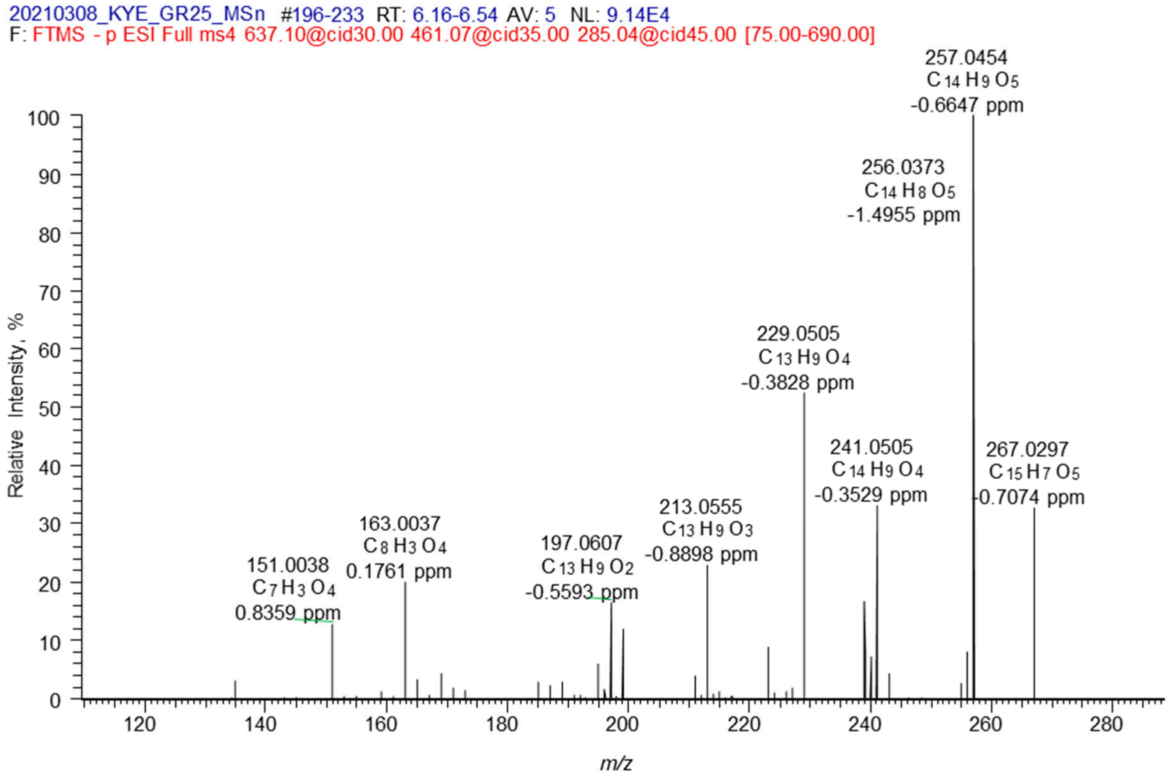

Figure S9-4. High-resolution electrospray ionization tandem mass spectrum (HR-ESI-MS^4 [637→ 461 → 285]) of the [M-H]$^-$ ion at *m/z* 637 corresponding to kaempferol-bis-3,7-β-D-glucuronide. The mass spectrum was acquired with a hybrid linear ion trap-orbital trap mass spectrometer (LIT-Orbitrap-MS) operated in negative ion mode with collision induced dissociation (relative collision energy CE 45%, trap resonance collisional activation CID 45%).

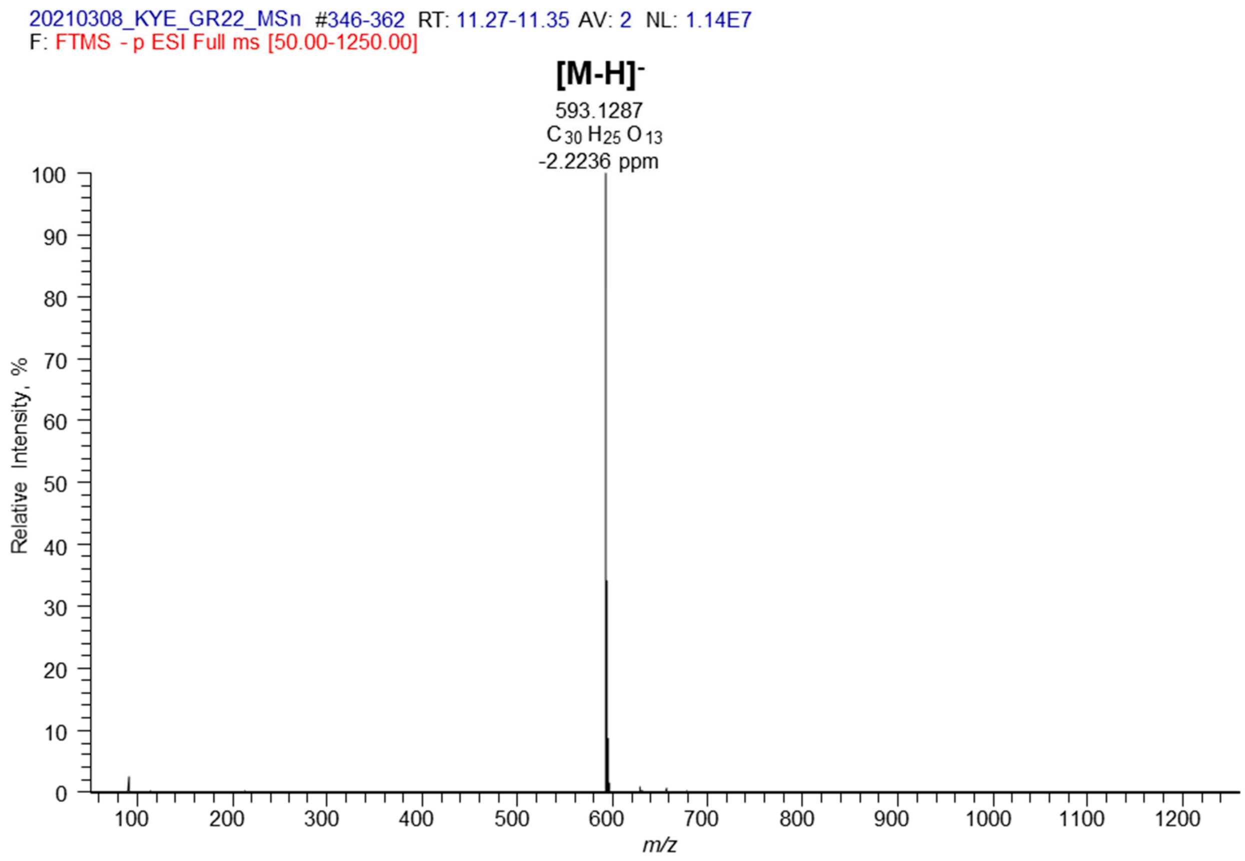

Figure S10-1. High-resolution electrospray ionization mass spectrum (HR-ESI-MS) with a signal of the [M-H]$^-$ ion at *m/z* 593 corresponding to 6''-(4-hydroxycinnamoyl)-astragalin. The mass spectrum was acquired with a hybrid linear ion trap-orbital trap mass spectrometer (LIT-Orbitrap-MS) operated in negative ion mode.

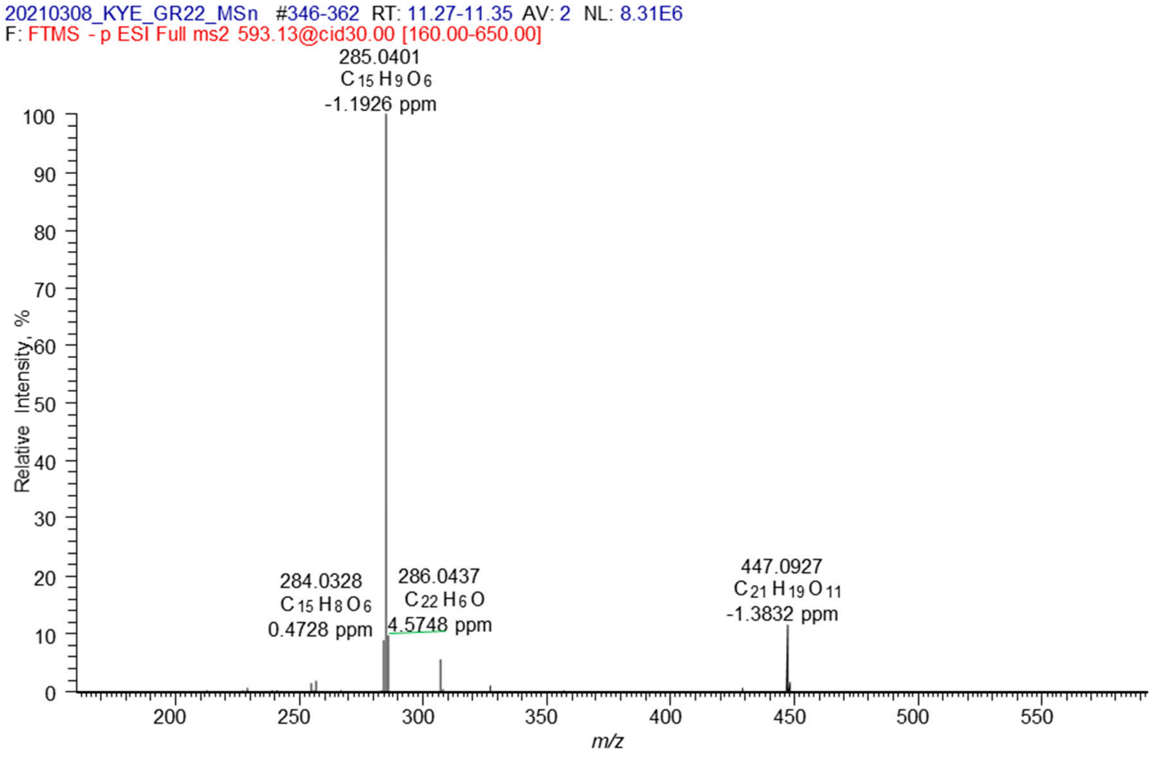

Figure S10-2. High-resolution electrospray ionization tandem mass spectrum (HR-ESI-MS^2) of the [M-H]$^-$ ion at *m/z* 593 corresponding to 6''-(4-hydroxycinnamoyl)-astragalin. The mass spectrum was acquired with a hybrid linear ion trap-orbital trap mass spectrometer (LIT-Orbitrap-MS) operated in negative ion mode with collision induced dissociation (relative collision energy CE 30%, trap resonance collisional activation CID 30%).

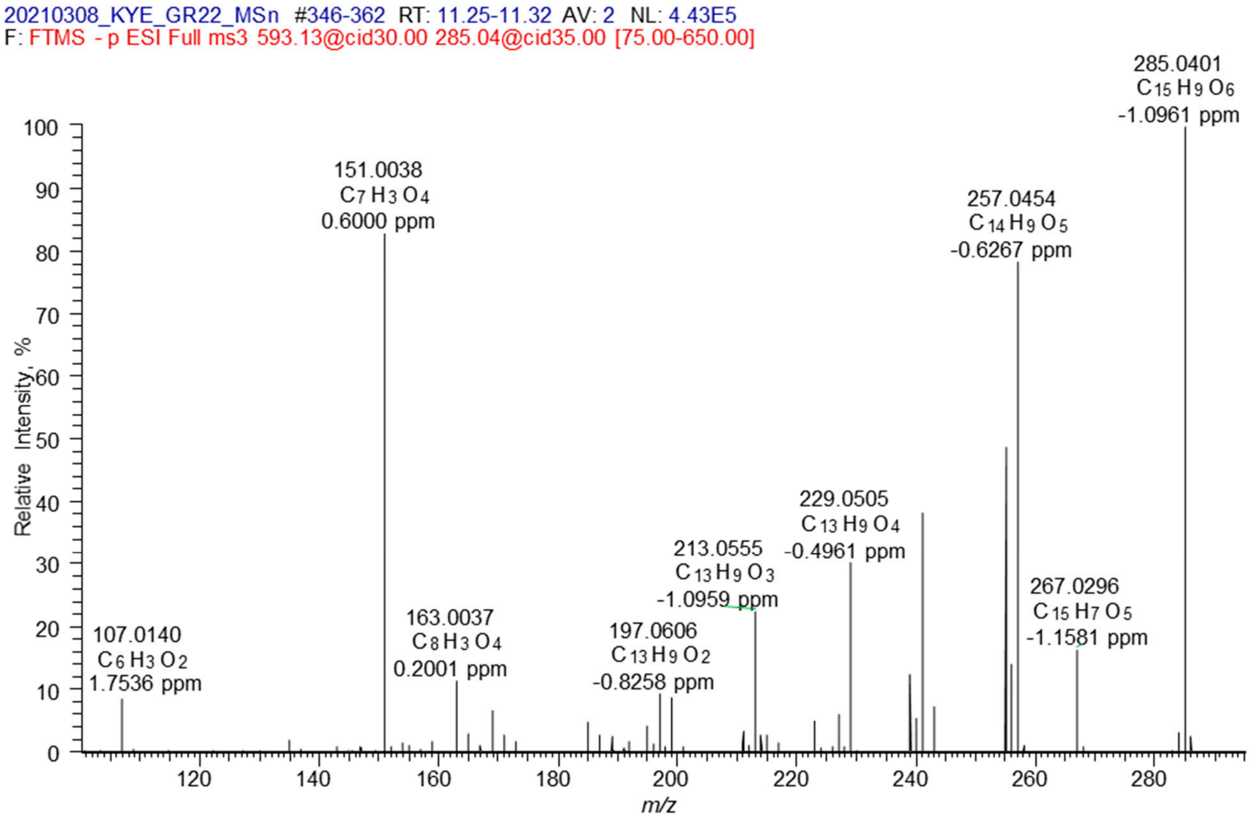

Figure S10-3. High-resolution electrospray ionization tandem mass spectrum (HR-ESI-MS^3 [593 → 285]) of the $[M-H]^-$ ion at *m/z* 593 corresponding to 6''-(4-hydroxycinnamoyl)-astragalin. The mass spectrum was acquired with a hybrid linear ion trap-orbital trap mass spectrometer (LIT-Orbitrap-MS) operated in negative ion mode with collision induced dissociation (relative collision energy CE 35%, trap resonance collisional activation CID 35%).

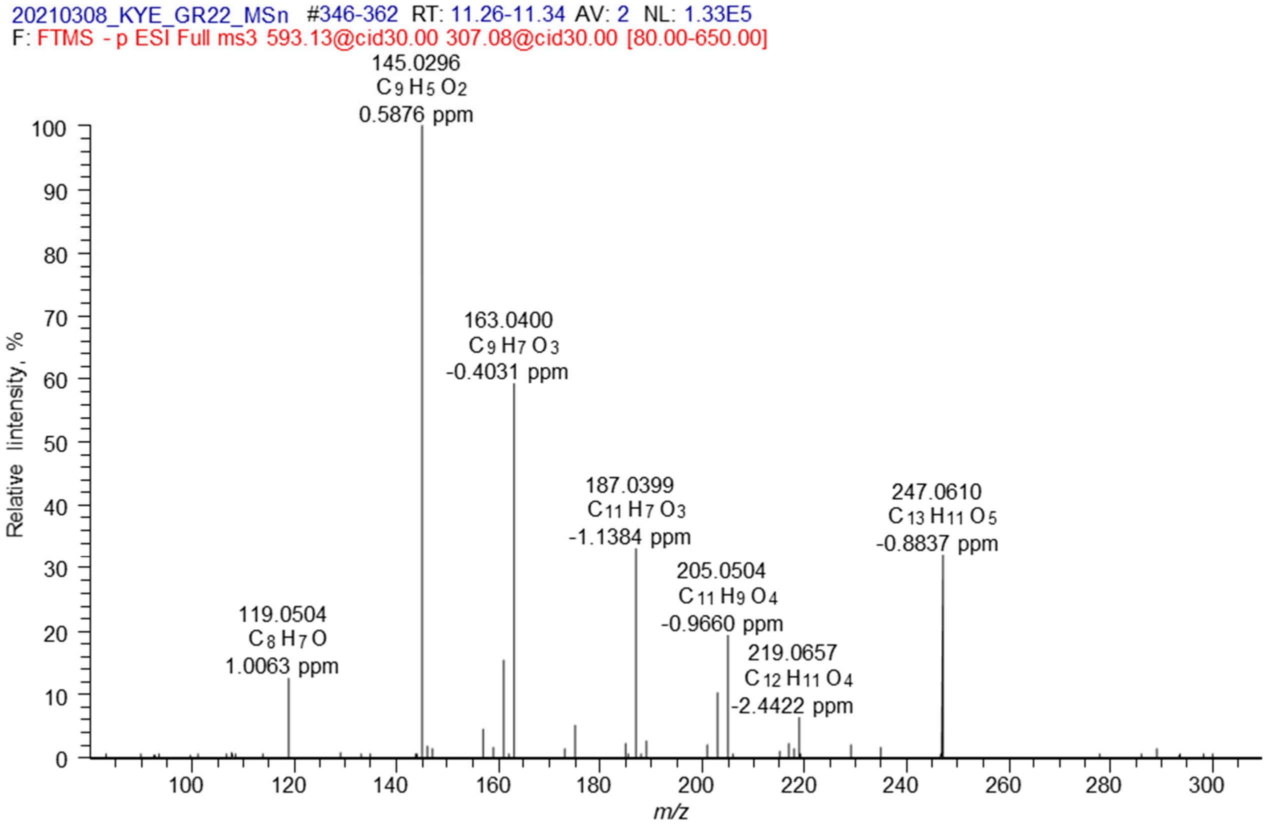

Figure S10-4. High-resolution electrospray ionization tandem mass spectrum (HR-ESI-MS^3 [593 → 307]) of the $[M-H]^-$ ion at *m/z* 593 corresponding to 6''-(4-hydroxycinnamoyl)-astragalin. The mass spectrum was acquired with a hybrid linear ion trap-orbital trap mass spectrometer (LIT-Orbitrap-MS) operated in negative ion mode with collision induced dissociation (relative collision energy CE 30%, trap resonance collisional activation CID 30%).

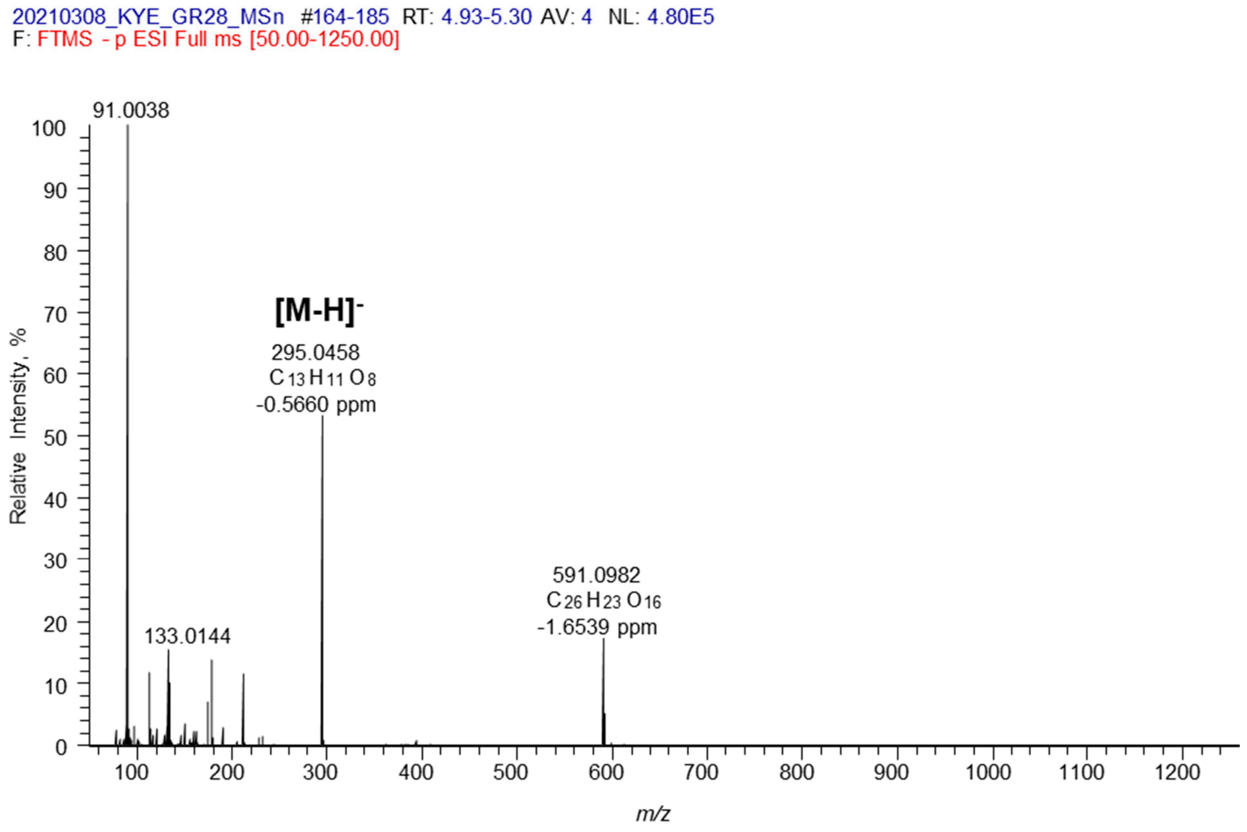

Figure S11-1. High-resolution electrospray ionization mass spectrum (HR-ESI-MS) with a signal of the $[M-H]^-$ ion at *m/z* 295 corresponding to caffeoyl malate. The mass spectrum was acquired with a hybrid linear ion trap-orbital trap mass spectrometer (LIT-Orbitrap-MS) operated in negative ion mode.

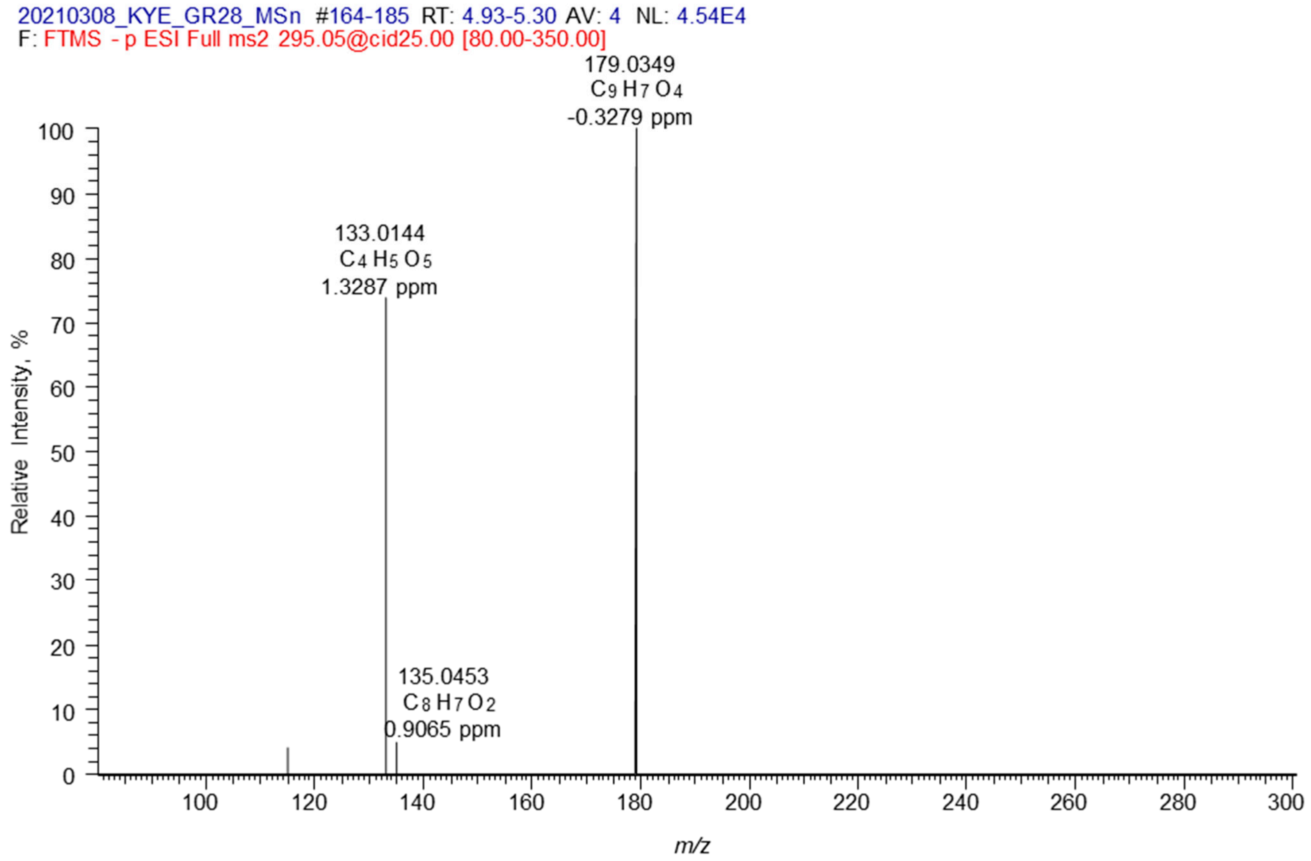

Figure S11-2. High-resolution electrospray ionization tandem mass spectrum (HR-ESI-MS^2) of the $[M-H]^-$ ion at *m/z* 295 corresponding to caffeoyl malate. The mass spectrum was acquired with a hybrid linear ion trap-orbital trap mass spectrometer (LIT-Orbitrap-MS) operated in negative ion mode with collision induced dissociation (relative collision energy CE 25%, trap resonance collisional activation CID 25%).

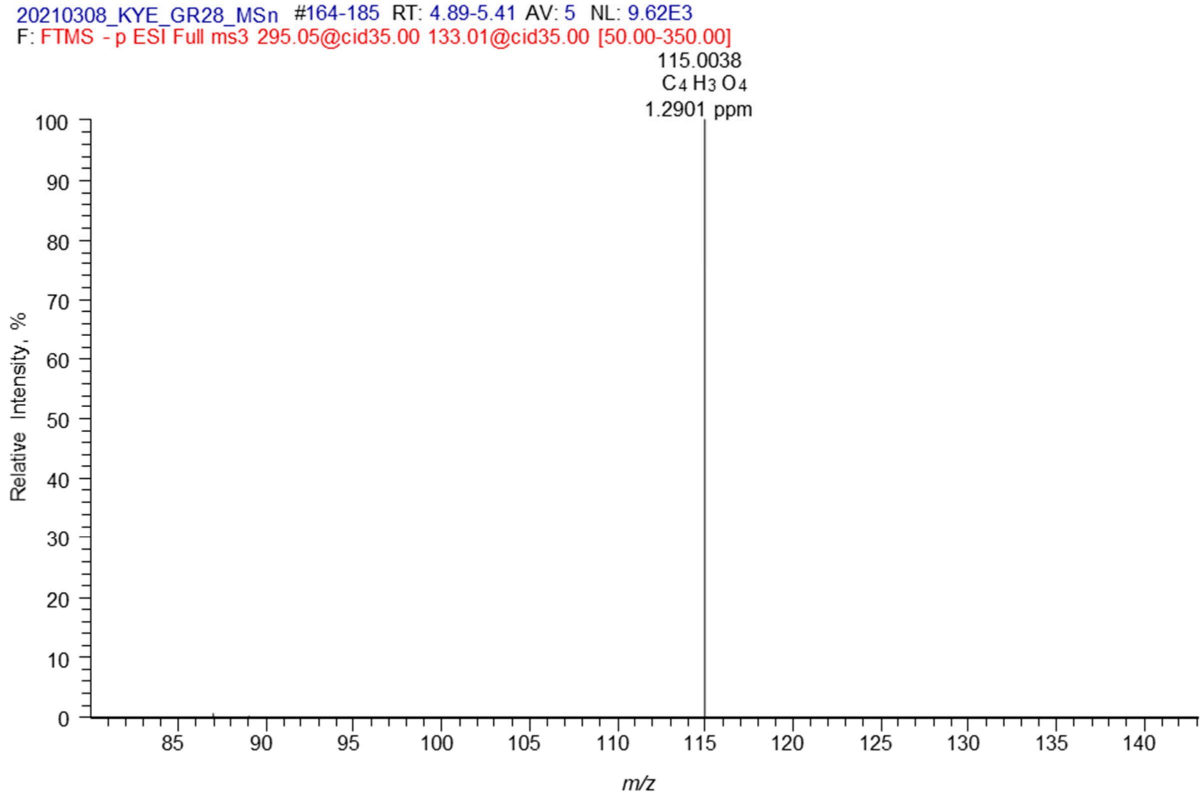

Figure S11-3. High-resolution electrospray ionization tandem mass spectrum (HR-ESI-MS^3 [295 → 133]) of the $[M-H]^-$ ion at *m/z* 295 corresponding to caffeoyl malate. The mass spectrum was acquired with a hybrid linear ion trap-orbital trap mass spectrometer (LIT-Orbitrap-MS) operated in negative ion mode with collision induced dissociation (relative collision energy CE 35%, trap resonance collisional activation CID 35%).

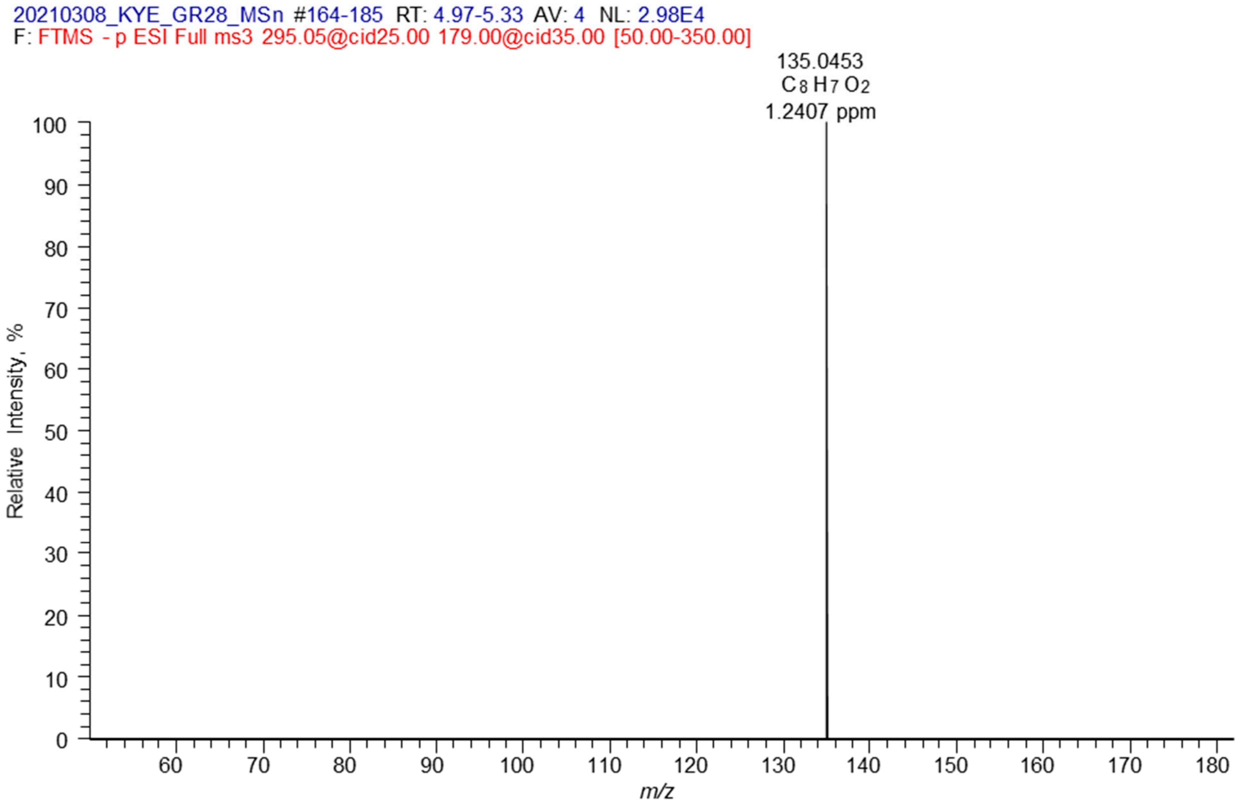

Figure S11-4. High-resolution electrospray ionization tandem mass spectrum (HR-ESI-MS^3 [295 → 179]) of the $[M-H]^-$ ion at *m/z* 295 corresponding to caffeoyl malate. The mass spectrum was acquired with a hybrid linear ion trap-orbital trap mass spectrometer (LIT-Orbitrap-MS) operated in negative ion mode with collision induced dissociation (relative collision energy CE 35%, trap resonance collisional activation CID 35%).

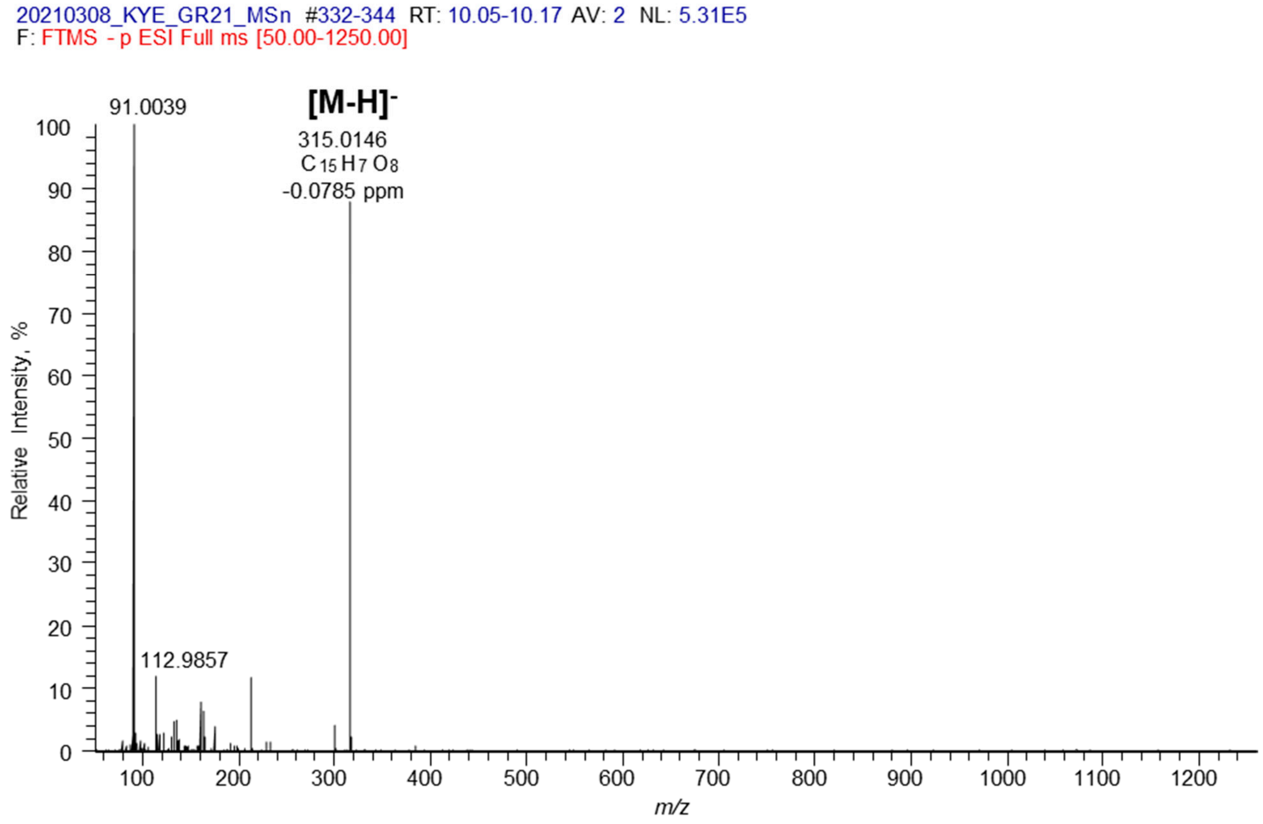

Figure S12-1. High-resolution electrospray ionization mass spectrum (HR-ESI-MS) of the $[M-H]^-$ ion at *m/z* 315 corresponding to 3-*O*-methylellagic acid. The mass spectrum was acquired with a hybrid linear ion trap-orbital trap mass spectrometer (LIT-Orbitrap-MS) operated in negative ion mode.

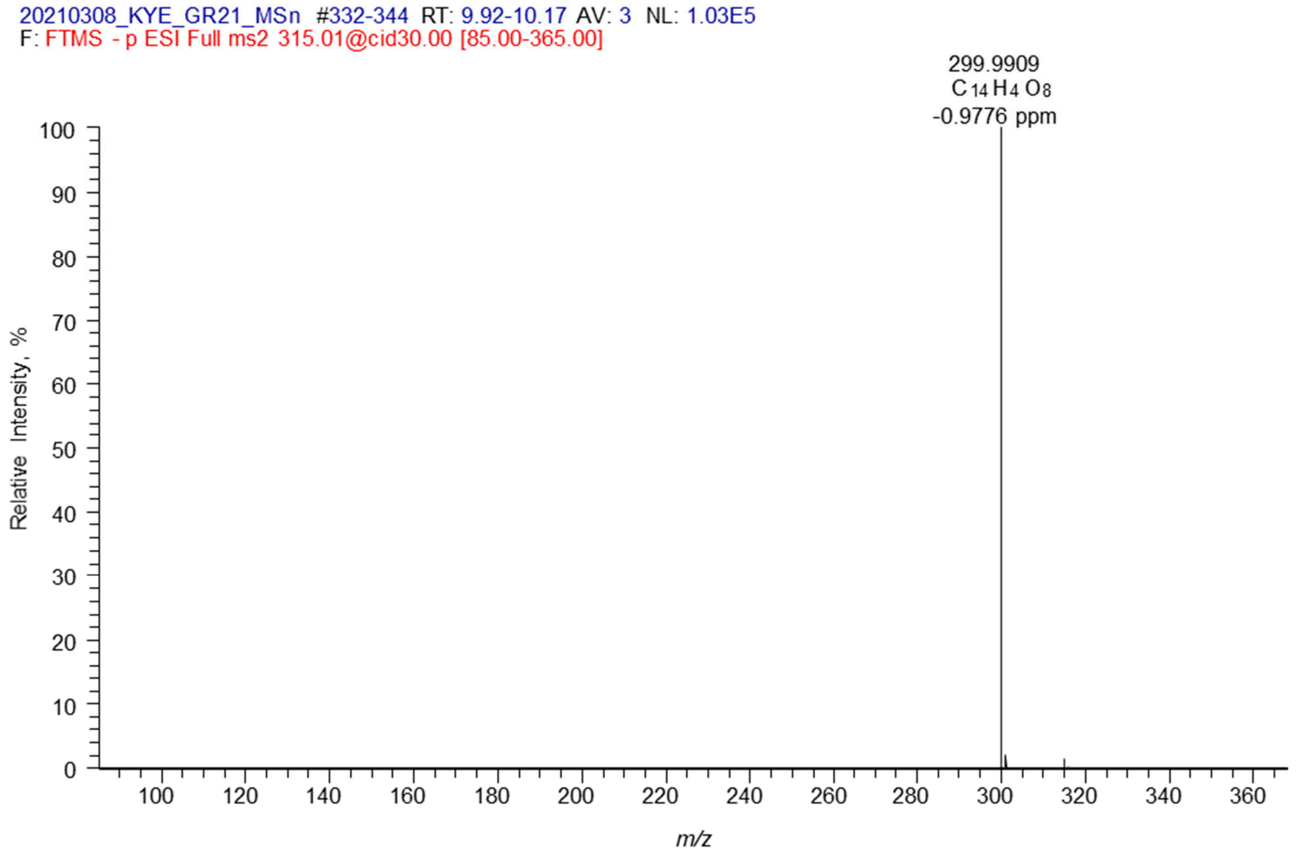

Figure S12-2. High-resolution electrospray ionization tandem mass spectrum (HR-ESI-MS^2) of the [M-H]$^-$ ion at *m/z* 315 corresponding to 3-*O*-methylellagic acid. The mass spectrum was acquired with a hybrid linear ion trap-orbital trap mass spectrometer (LIT-Orbitrap-MS) operated in negative ion mode with collision-induced dissociation (relative collision energy CE 30%, trap resonance collisional activation CID 30%).

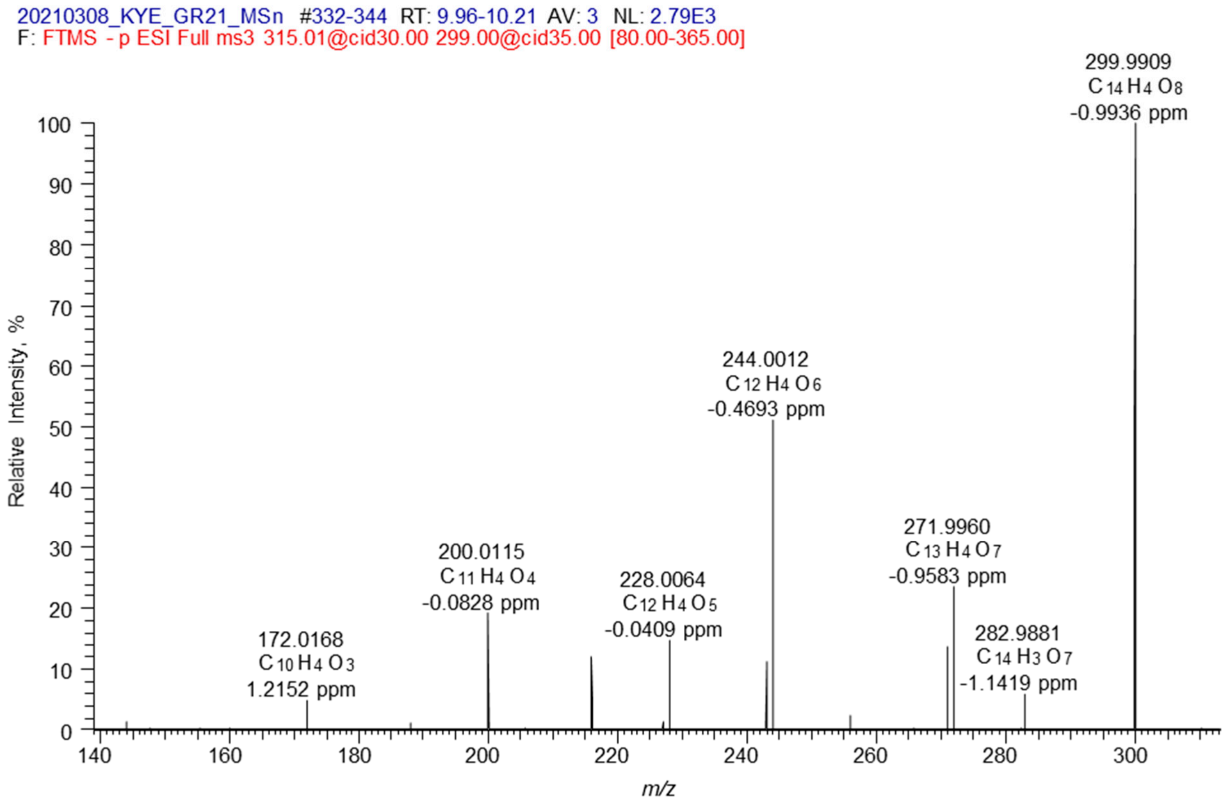

Figure S12-3. High-resolution electrospray ionization tandem mass spectrum (HR-ESI-MS^3, 315 → 299) of the $[M-H]^-$ ion at *m/z* 315 corresponding to 3-*O*-methylellagic acid. The mass spectrum was acquired with a hybrid linear ion trap-orbital trap mass spectrometer (LIT-Orbitrap-MS) operated in negative ion mode with collision-induced dissociation (relative collision energy CE 35%, trap resonance collisional activation CID 35%).

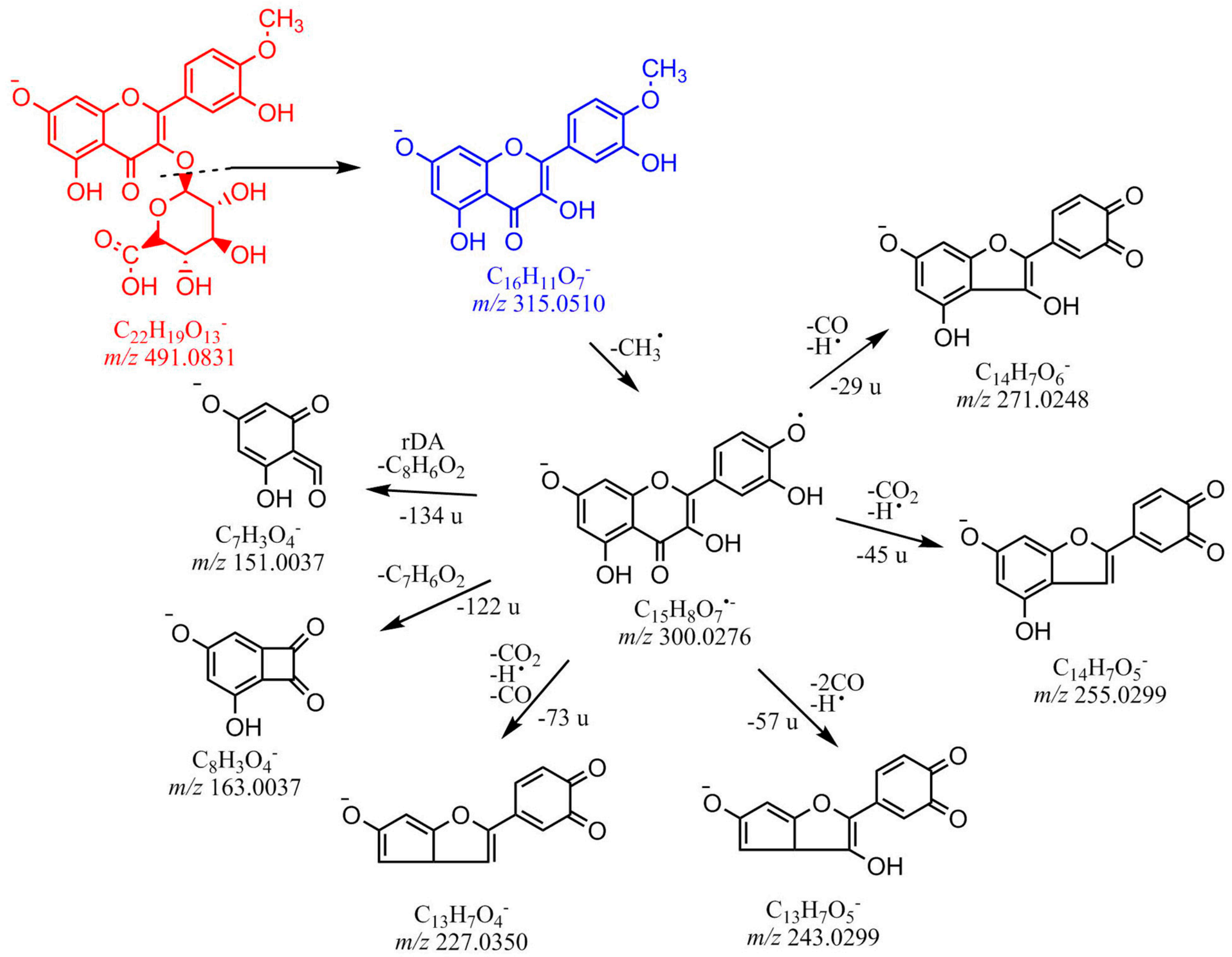

Scheme S1. Proposed tandem mass spectrometric fragmentation patterns for the ion at *m/z* 491 ($[M-H]^-$) corresponding to isorhamnetin-3-*O*-β-D-glucuronide (MS^2, red) and its fragments found at *m/z* 315 (MS^3, blue) and 300 (MS^4, black). rDA - Retro-Diels-Alder reaction

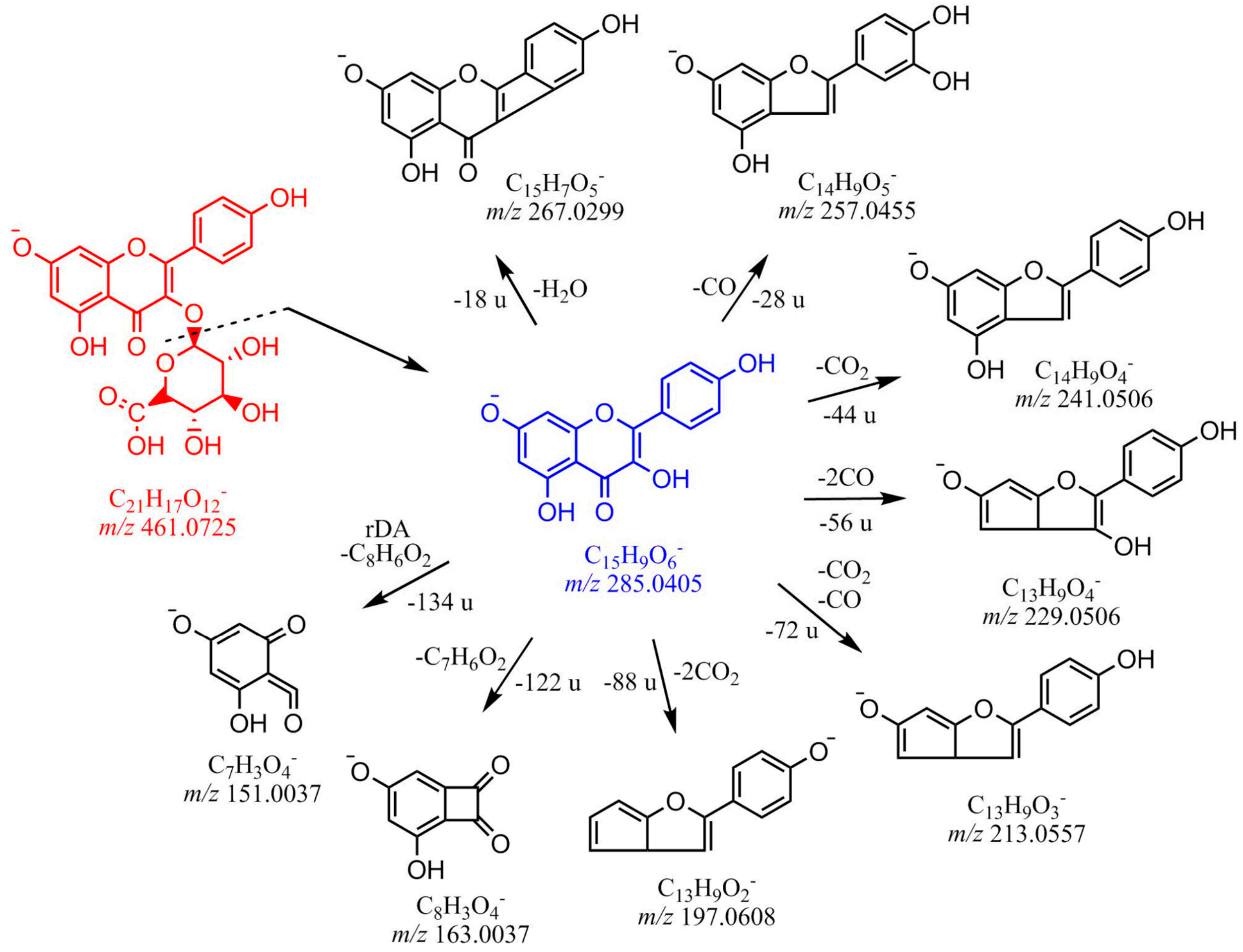

Scheme S2. Proposed tandem mass spectrometric fragmentation patterns for *m/z* 461($[M-H]^-$) corresponding to kaempferol-3-*O*-β-D-glucuronide (MS, red) and its fragments (MS^2 – blue, MS^3 – black). rDA - Retro-Diels-Alder reaction

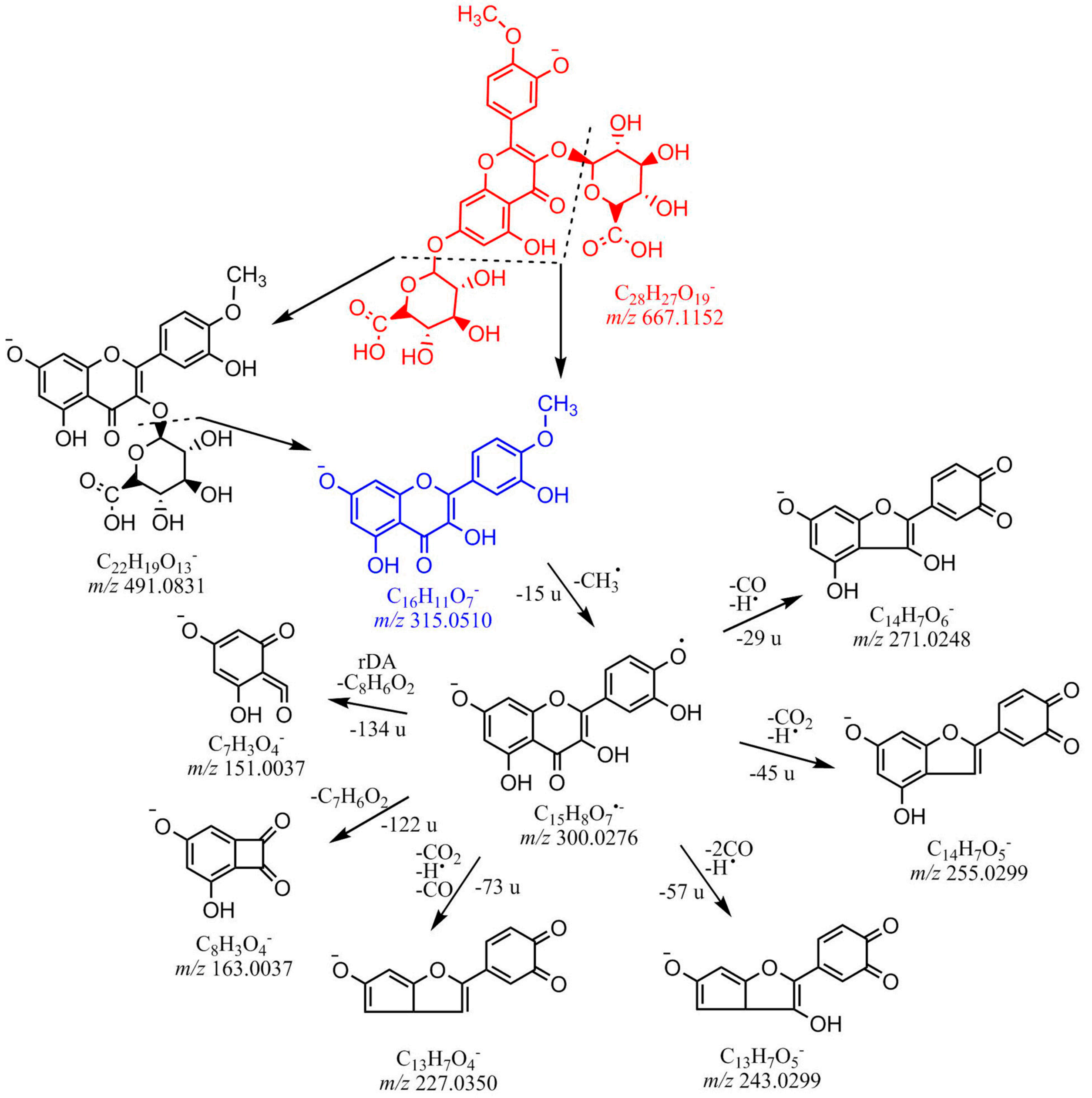

Scheme S3. Proposed tandem mass spectrometric fragmentation patterns for the ion at *m/z* 667 ($[M\text{-}H]^-$, MS^2, red) corresponding to isorhamnetin-bis-3,7-*O*-β-D-glucuronide and its fragments found at *m/z* 491 (MS^3, black), 315 (MS^4, blue) and 300 (MS^5, black). rDA - Retro-Diels-Alder reaction

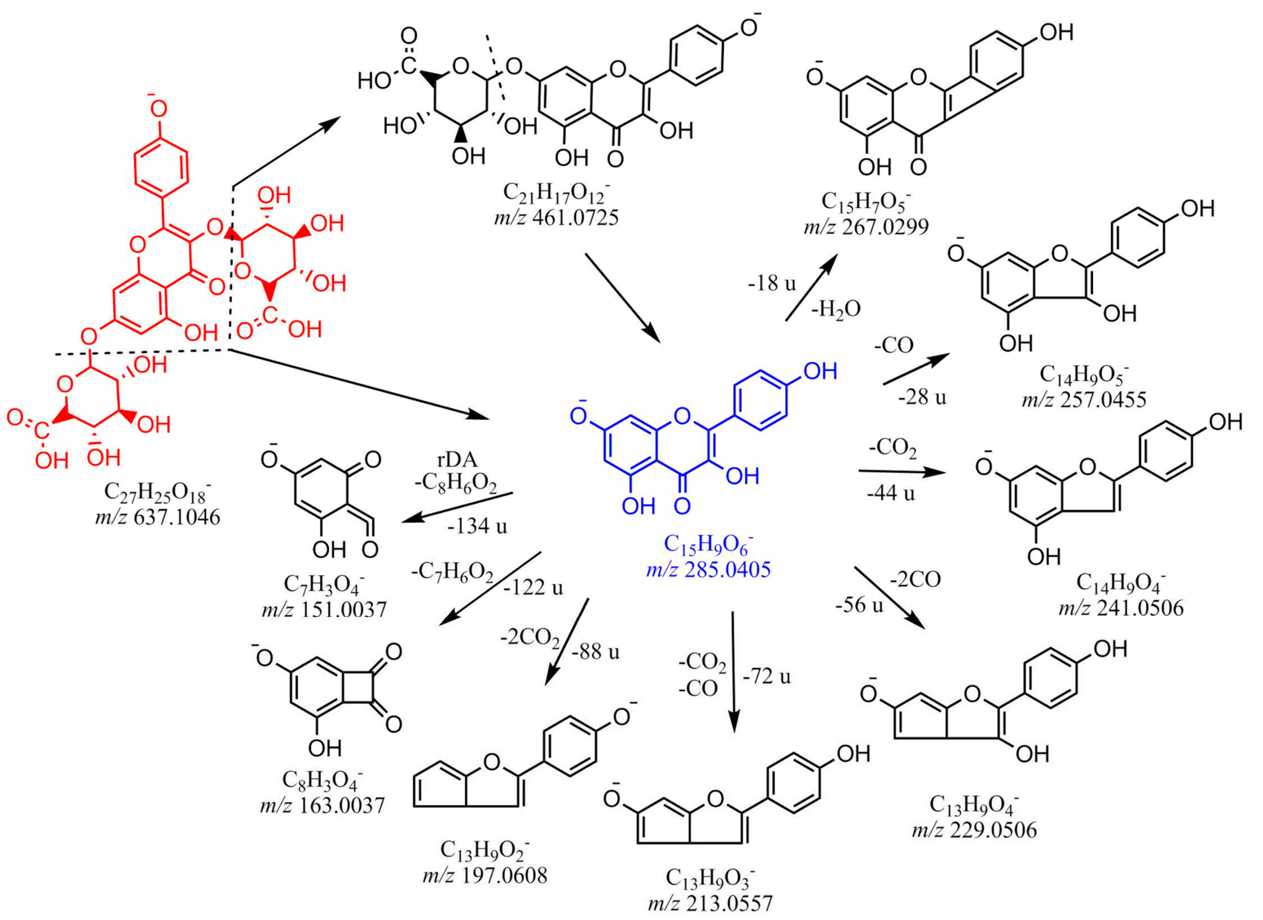

Scheme S4. Proposed tandem mass spectrometric fragmentation patterns for the ion at *m/z* 637 ([M-H]$^-$) corresponding to kaempferol-bis-3,7-*O*-β-D-glucuronide (MS^2, red) and its fragment found at *m/z* 461 (MS^3, blue) and 285 (MS^4, black). rDA - Retro-Diels-Alder reaction

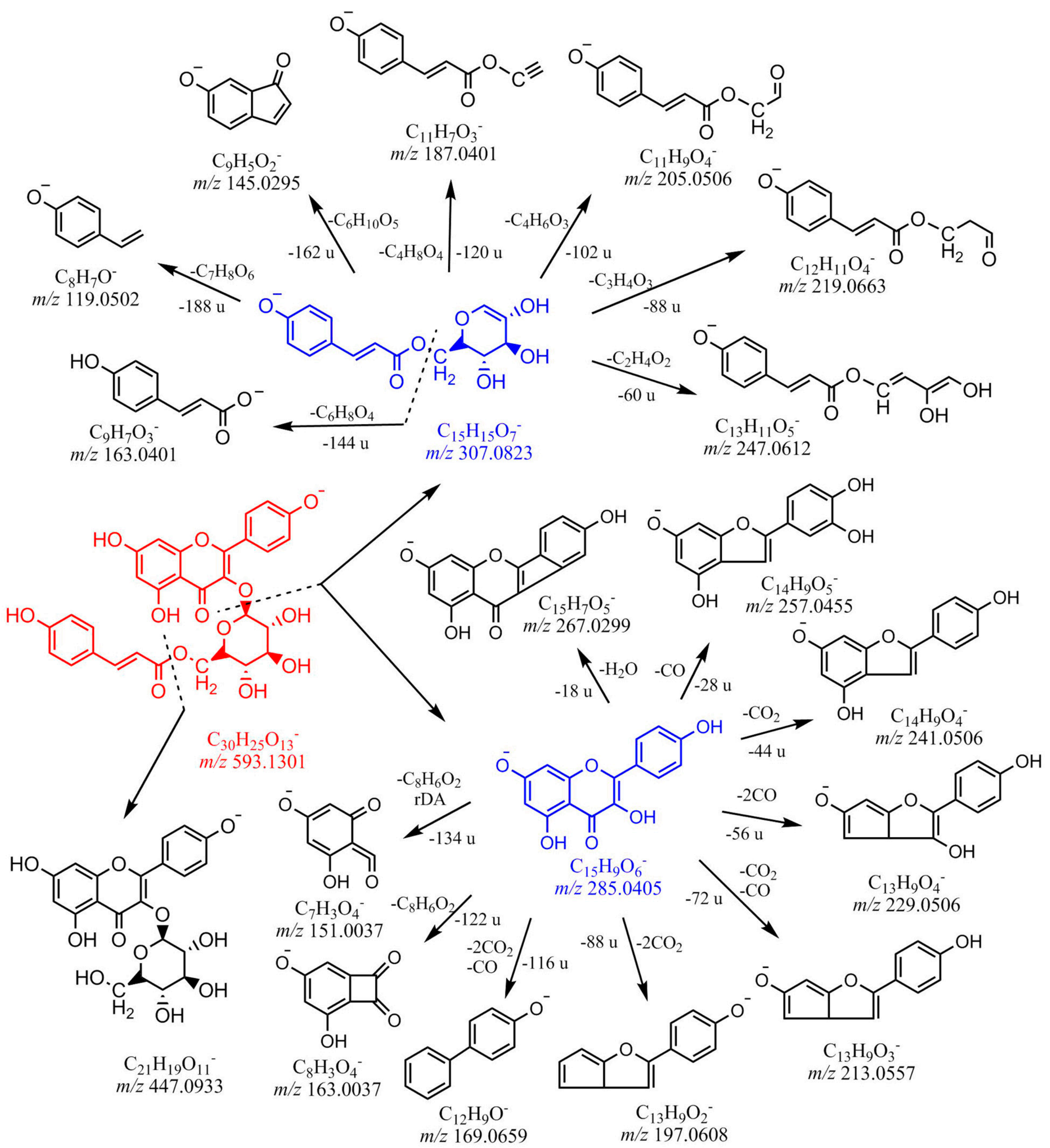

Scheme S5. Proposed tandem mass spectrometric fragmentation patterns for the ion at *m/z* 593 ($[M-H]^-$, MS^2, red) corresponding to 6"-(4-hydroxycinnamoyl)-astragalin and its fragments found at *m/z* 307 (MS^3, blue) and 285 (MS^3, blue). rDA - Retro-Diels-Alder reaction

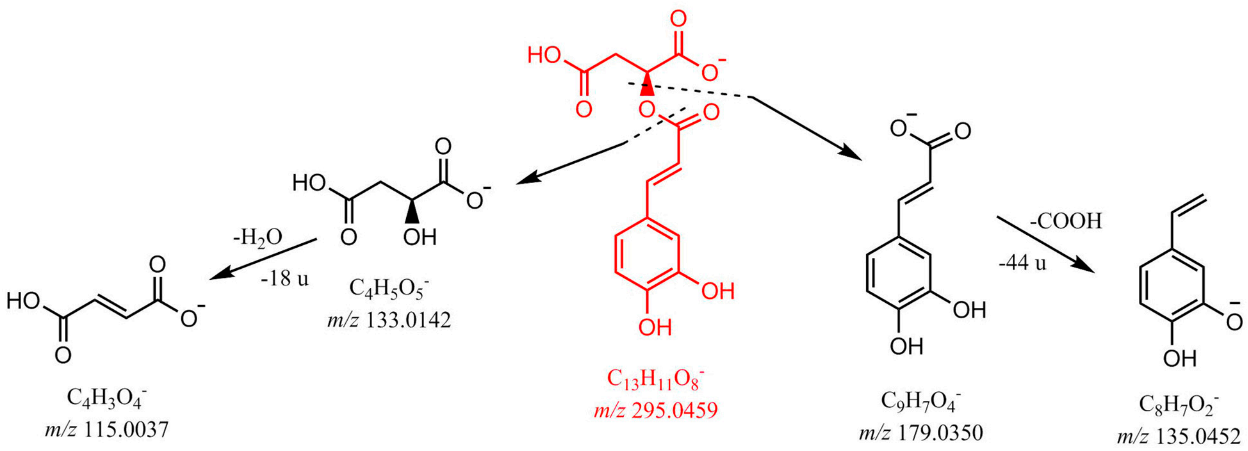

Scheme S6. Proposed tandem mass spectrometric fragmentation patterns for the ion at *m/z* 295 ($[M-H]^-$) corresponding to caffeoyl malate (MS^2, red) and its fragments found at *m/z* 179 (MS^3, black) and 133 (MS^3, black)

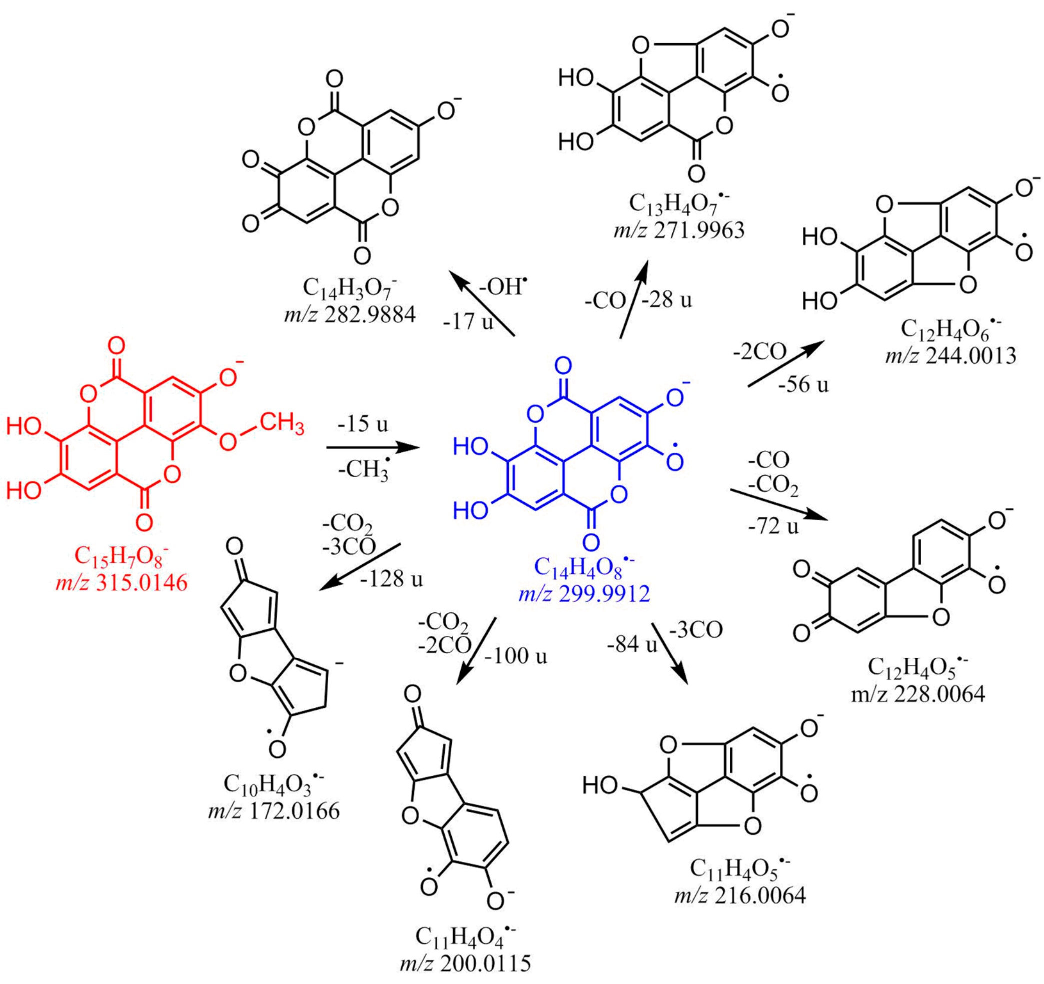

Scheme S7. Proposed tandem mass spectrometric fragmentation patterns for the ion at *m/z* 315 ($[M\text{-}H]^-$, MS^2, red) corresponding to 3-*O*-methylellagic acid and its fragment found at *m/z* 299 (MS^3, blue).

References

1. Montero, L.; Meckelmann, S.W.; Kim, H.; Ayala-Cabrera, J. F.; Schmitz, O. J. Differentiation of industrial hemp strains by their cannabinoid and phenolic compounds using LC × LC-HRMS. *Anal Bioanal Chem* **2022,** *414*, 5445–5459. doi: 10.1007/s00216-022-03925-8

2. Faugno, S.; Piccolella, S.; Sannino, M.; Principio, L.; Crescente, G.; Baldi, G. M.; Fiorentino, N.; Pacifico, S. Can agronomic practices and cold-pressing extraction parameters affect phenols and polyphenols content in hempseed oils? *Ind. Crops and Prod.* **2019**, *130,* 511–519. doi:10.1016/j.indcrop.2018.12.084

3. Spínola, V.; Pinto, J.; Llorent-Martínez, E. J.; Tomás, H.; Castilho, P. C. Evaluation of Rubus grandifolius L. (wild blackberries) activities targeting management of type-2 diabetes and obesity using in vitro models. *Food Chem. Toxicol.* **2019**, *123*, 443-452. doi:10.1016/j.fct.2018.11.006

4. Manurung, J.; Kappen, J.; Schnitzler, J.; Frolov, A.; Wessjohann, L.A.; Agusta, A.; Muellner-Riehl, A.N.; Franke, K. Analysis of unusual sulfated constituents and anti-infective properties of two Indonesian mangroves, *Lumnitzera littorea* and *Lumnitzera racemosa* (*Combretaceae*). *Separations* **2021**, *8*, 82. https://doi.org/10.3390/ separations8060082

5. Al-Wahaibi, L; H., Al-Saleem, M. S. M.; Basudan, O. A.; Abdel-Mageed, W. M. Flavonoid dimers from the aerial parts of *Conyza stricta. Biochem. Syst. Ecol,* **2019***, 87,* 103959. doi:10.1016/j.bse.2019.103959

6. March, R. E.; Miao, X.-S. A fragmentation study of kaempferol using electrospray quadrupole time-of-flight mass spectrometry at high mass resolution. *Int. J. Mass Spectrom.,* **2004***, 231(2-3),* 157–167. doi:10.1016/j.ijms.2003.10.008
